# Crystal structures of six complexes of phosphane chalcogenides *R*^1^*R*^2^*R*^3^P*E* (*R* = *tert*-butyl or isopropyl, *E* = S or Se) with the metal halides *MX*_2_ (*M* = Pd or Pt, *X* = Cl or Br), two halochalcogenyl­phospho­nium derivatives (^*t*^Bu_2_^i^PrP*E*Br)_2_[Pd_2_Br_6_] and one hydrolysis product

**DOI:** 10.1107/S2056989025000805

**Published:** 2025-02-04

**Authors:** Daniel Upmann, Peter G. Jones

**Affiliations:** a Institut für Anorganische und Analytische Chemie, Technische Universität Braunschweig, Hagenring 30, D-38106 Braunschweig, Germany; Universität Greifswald, Germany

**Keywords:** crystal structure, palladium, platinum, phosphane chalcogenides, secondary inter­actions

## Abstract

The *L*_2_*MX*_2_ complexes **1**–**5** (**1**: *L* = ^*t*^Bu^i^Pr_2_PSe, *M* = Pd, *X* = Cl; **2**: *L* = ^*t*^Bu_2_^i^PrPSe, *M* = Pd, *X* = Cl; **3**: *L* = ^*t*^Bu_2_^i^PrPSe, *M* = Pd, *X* = Br; **4**: *L* = ^*t*^Bu_2_^i^PrPS, *M* = Pd, *X* = Br; **5**: *L* = ^*t*^Bu_2_^i^PrPS, *M* = Pt, *X* = Cl) {systematic names: (*tert*-butyl­diiso­propyl­phosphine selenide-κ*Se*)di­chlorido­palladium(II), [PdCl_2_(C_10_H_23_PSe)_2_] (**1**), (di-*tert*-butyl­iso­propyl­phosphine selenide-κ*Se*)di­chloridopalladium(II), [PdCl_2_(C_11_H_25_PSe)_2_] (**2**), di­bromido­(di-*tert*-butyl­iso­propyl­phosphine selenide-κ*Se*)palladium(II), [PdBr_2_(C_11_H_25_PSe)_2_] (**3**), di­bromido­(di-*tert*-butyl­iso­propyl­phosphine sulfide-κ*S*)palladium(II), [PdBr_2_(C_11_H_25_PS)_2_] (**4**), di­chlorido­(di-*tert*-butyl­iso­propyl­phosphine sulfide-κ*S*)palladium(II), [PdCl_2_(C_11_H_25_PS)_2_] (**5**)} all display a *trans* configuration with square-planar geometry at the metal atom. Compounds **2** and **3** are isotypic. The mol­ecules of **1** and **4** display crystallographic inversion symmetry; compound **5** involves two independent mol­ecules, each with inversion symmetry but with differing orientations of the tri­alkyl­phosphane groups. Chemically equivalent bond lengths all lie in narrow ranges, whereby the values for palladium and platinum compounds scarcely differ. Compound **6**, (^*t*^Bu^i^Pr_2_PS)_2_Pd_2_Cl_4_ {systematic name: di-μ-chlorido-bis­[(*tert*-butyldiiso­propyl­phosphine sulfide-κ*S*)chlorido­palladium(II)], [PdCl_2_(C_10_H_23_PS)_2_]}, is dinuclear with a central Pd_2_Cl_2_ ring, and displays crystallographic inversion symmetry. The bonds to the bridging are longer than those to the terminal chlorine atoms; the Pd—S bond is shorter than the *M*—S bonds in **4** and **5**, reflecting the weaker *trans* influence of (bridging) chlorine compared to sulfur. Compounds **7** and **8**, 2(^*t*^Bu_2_^i^PrP*E*Br)^+^ [Pd_2_Br_6_]^2−^ with *E* = S for **7** and Se for **8** {systematic names: (bromo­sulfan­yl)di-*tert*-butyl­iso­propyl­phosphanium di-μ-bromido-bis­[di­bromido­palladium(II)], (C_11_H_25_BrPS)_2_[Pd_2_Br_6_] (**7**) and (bromo­selan­yl)di-*tert*-butyl­iso­propyl­phosphanium di-μ-bromido-bis­[di­bromido­palladium(II)], (C_11_H_25_BrPS_2_)_2_[Pd_2_Br_6_], (**8**)}, were obtained by oxidizing the appropriate Pd^II^ precursors with elemental bromine; they are not isotypic. The ions are connected by very short halogen bonds Br⋯Br. For both compounds, two *E*⋯Br contacts further link the cations and anions to form ribbons. Compound **9** {systematic name: bis­[dimeth­yl(sulfanyl­idene)phosphin­ito-κ*Se*]bis­(hy­droxy­diiso­propyl­phosphine selenide-κ*Se*)palladium(II), [Pd(C_6_H_14_OP)_2_(C_6_H_15_OP)_2_], {(^i^Pr_2_PSeO)_2_H}_2_Pd, is a hydrolysis product with inversion symmetry and contains an intra­molecular P—O⋯H—O—P group with a disordered hydrogen atom. Compounds **1**–**6** and **9** show few, if any, short inter­molecular contacts, although some H⋯*M* contacts are observed. A problem with atom-type assignment for structure refinement is discussed.

## Abstract 2

Various compounds involving tri­alkyl­phosphane chalcogenides (or their derivatives) and palladium or platinum dihalides were structurally characterized and their intra- and inter­molecular inter­actions and metrical parameters were analysed. General comments on the SFAC command are also included.

## 1. Chemical context

We are inter­ested in metal complexes of tri­alkyl­phosphane chalcogenide ligands *R*^1^*R*^2^*R*^3^P*E* (*R* = *tert*-butyl or isopropyl, *E* = S or Se; here we use the general abbreviation *L* for these ligands). In a recent series of papers in this journal (Upmann *et al.*, 2024*a*–*e* ▸ ▸ ▸ ▸ ▸; much introductory material is given in the first of these publications) we have reported on the gold(I) complexes *L*Au*X* (*X* = Cl or Br) and their oxidation with elemental bromine or the chlorine equivalent iodo­benzene dichloride, PhICl_2_. The two main series of products were the simple gold(III) complexes *L*Au*X*_3_ and the doubly oxidized halochalcogenyl­phospho­nium derivatives (*R*^1^*R*^2^*R*^3^P*EX*)[Au*X*_4_], corresponding to the addition of two or four halogen atom equivalents, respectively, per metal atom.
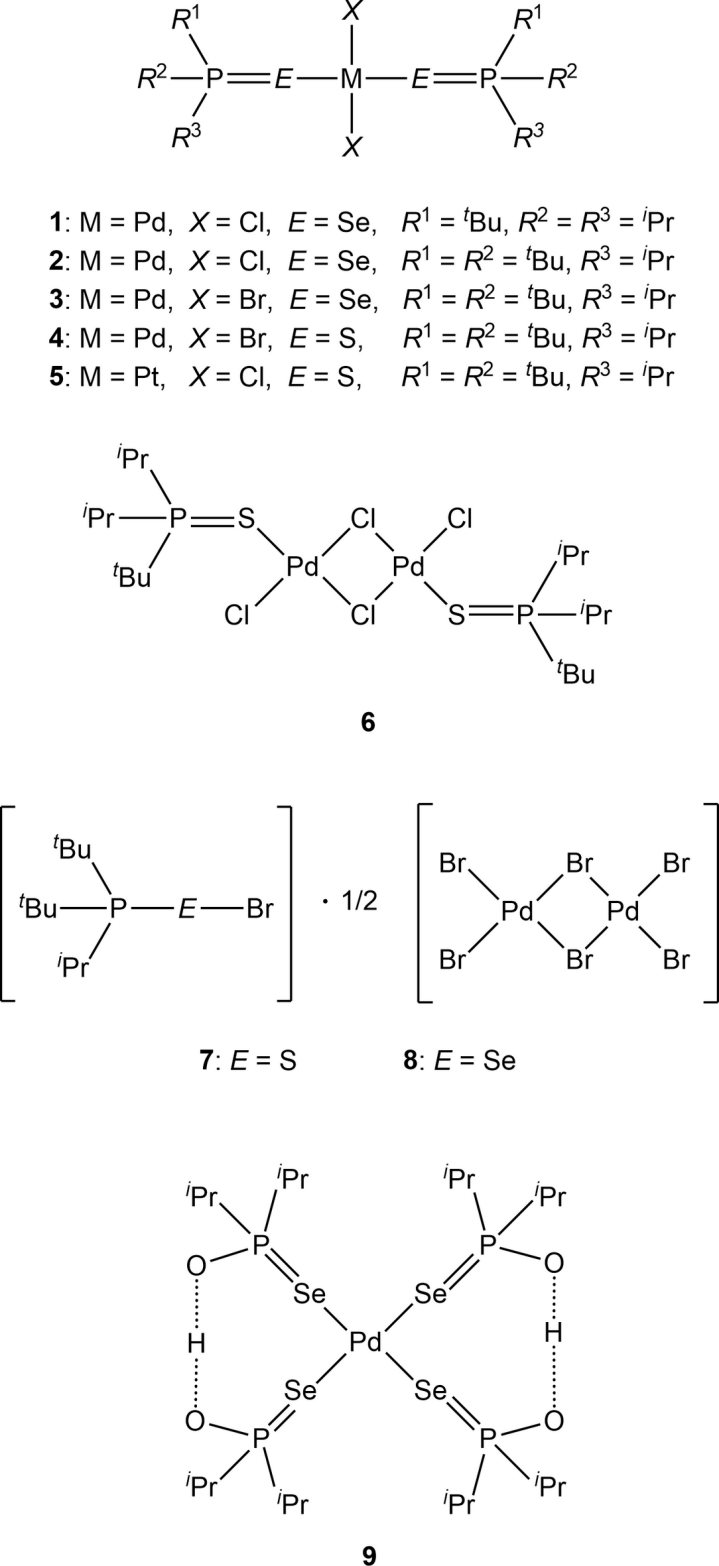


We decided to extend our studies to palladium(II) or platinum(II) complexes *L*_2_*MX*_2_ (*M* = Pd or Pt) in the hope that these could be similarly oxidized to give *M*(IV) derivatives. Although the *M*^II^ precursors *L*_2_*MX*_2_ proved to be generally accessible, all attempts to oxidize *L*_2_*M*Cl_2_ using iodo­benzene dichloride led to immediate decomposition (with formation of a black precipitate), whereas bromine was too weak an oxidizing agent to convert *M*^II^ to *M*^IV^, leading instead to only two isolable complexes (^*t*^Bu_2_^i^PrP*E*Br)_2_(Pd_2_Br_6_), whereby the ratio of ligands *L* to palladium atoms thus changes from 2 to 1. The investigations were therefore not continued. Here we present the structures of four complexes *L*_2_Pd*X*_2_ (**1**–**4**), one complex *L*_2_PtCl_2_ (**5**), one dinuclear complex (^*t*^Bu^i^Pr_2_PS)_2_Pd_2_Cl_4_ (**6**), the two bromo­chalcogenyl­phospho­nium derivatives (^*t*^Bu_2_^i^PrP*E*Br)_2_(Pd_2_Br_6_) (**7**, *E* = S; **8**, *E* = Se) and one hydrolysis product {(^i^Pr_2_PSeO)_2_H}_2_Pd (**9**).

## 2. Structural commentary

**Figure 1:**
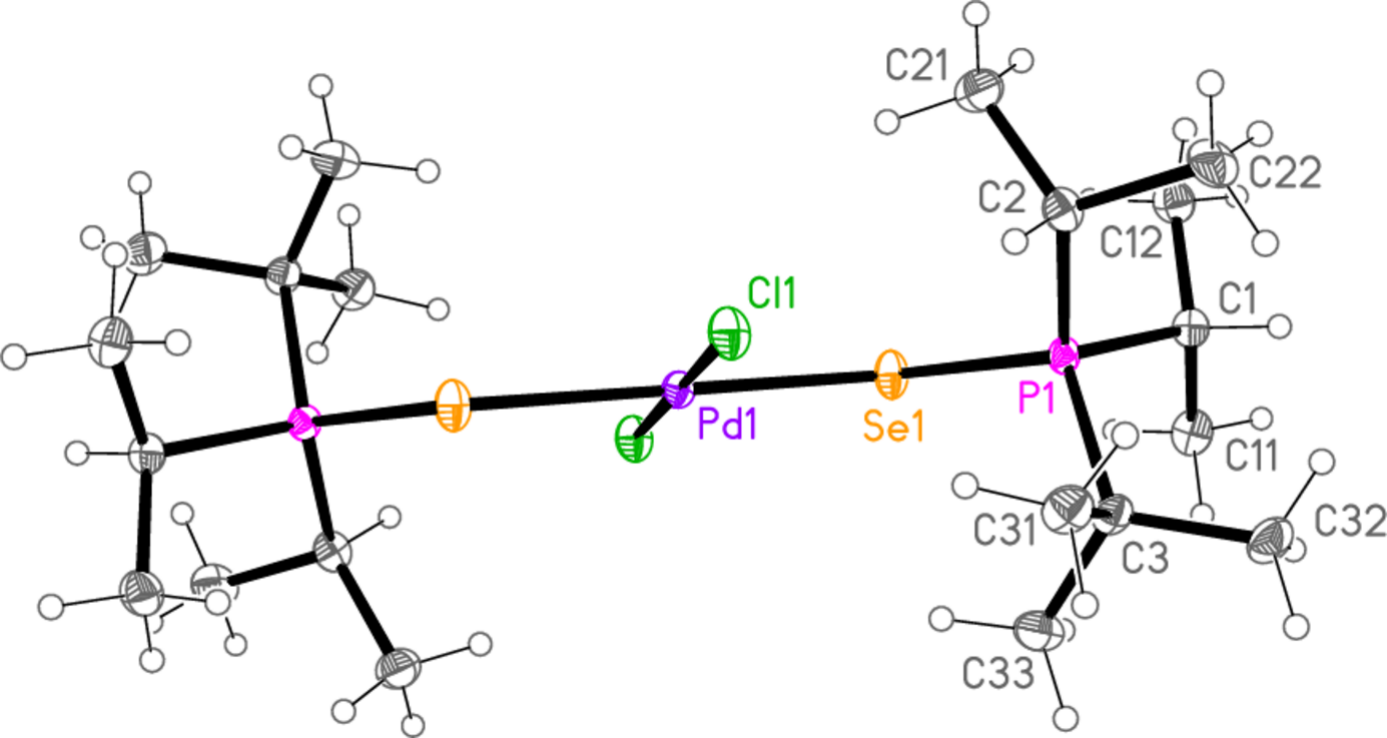
The mol­ecule of compound **1** in the crystal. Only the asymmetric unit is labelled.

All compounds crystallized solvent-free. Selected mol­ecular dimensions are given in Tables 1 ▸–9 ▸ ▸ ▸ ▸ ▸ ▸ ▸ ▸. The structures are shown in Figs. 1 ▸–9 ▸ ▸ ▸ ▸ ▸ ▸ ▸ ▸, with ellipsoids at the 50% level. The dashed bonds in Figs. 7 ▸ and 8 ▸ correspond to short inter­ionic contacts that are discussed in *Supra­molecular features*. For simplicity we write the P—*E* bonds in the text as single bonds, although they are often written as double bonds P=*E* in older literature (and indeed in the scheme). Primes (’) are used to denote generalized or previously defined symmetry operators.

The simple *L*_2_*MX*_2_ complexes **1**–**5** (Figs. 1 ▸–5 ▸ ▸ ▸ ▸) all show the expected square-planar geometry at the metal atom. Despite the planarity, two of the four Se—Pd—*X* angles in **2** and in **3** differ by *ca*. 10° from the ideal 90°. The largest deviation from planarity for the metal atom and its four immediate neighbours *E* and *X* is observed for **3**, with a mean deviation of 0.052 Å. The ligands adopt a *trans* configuration. Compounds **2** and **3** are isotypic. The mol­ecules of **1** and **4** display crystallographic inversion symmetry; compound **5** involves two independent mol­ecules, each with inversion symmetry. With one exception, one of the three torsion angles *E*⋯*E*—P—C*n* (omitting the metal atom as the central atom of a linear group), which all differ by ca. 120°, is close to ±180°, and this atom, belonging to a *tert*-butyl group, is given the lowest numbering (C1 or C4). The exception is the second independent mol­ecule of compound **5**, for which all the torsion angles of this type are some 30° greater than for the first mol­ecule; the isopropyl groups are at C3 and C5 and thus formally change places in the rotational sequence (see Fig. 10 ▸). Chemically equivalent bond lengths all lie in narrow ranges, whereby the values for palladium (**1**–**4**) and platinum (**5**) compounds scarcely differ. Average bond lengths for the ligands are P—S = 2.028 and P—Se = 2.190 Å; these are closely similar to those in the related gold(I) derivatives *L*Au*X* (2.037, 2.194 Å; Upmann *et al.*, 2024*a* ▸) and [*L*_2_Au][Au*X*_4_] (2.032, 2.193 Å; Upmann *et al.*, 2024*d* ▸) but slightly shorter than the values of 2.060, 2.218 Å for the gold(III) series *L*Au*X*_3_ (Upmann *et al.*, 2024*b* ▸), probably reflecting a somewhat greater contribution of the resonance form with a purely single P—*E* bond in the latter. The bond angles P—*E*—*M* were found to vary appreciably for the three series of gold compounds, with ranges of around 5° (and one outlier for ^*t*^Bu_3_PSAuCl_3_, attributed tentatively to steric effects), although the average values were consistently smaller for Se than for S derivatives. For compounds **1**–**5**, the average P—*E*—*M* angles are 114.8° and 114.2° for S and Se, respectively, several degrees higher than those for the gold compounds. Furthermore, the Se values range from 107–119° and differ by over 10° for the two independent P—Se—Pd bond angles of **2** and **3**; perhaps this is in some way connected with the irregular Se—Pd—*X* angles in these compounds, but we can think of no simple reason for this. The *M*—*X* bond lengths are closely similar, with averages of 2.311 Å for *X* = Cl (with no significant difference for the Pd and Pt derivatives) and 2.447 Å for *X* = Br; the same applies to the *E*—*X* bond lengths, with averages of 2.332 Å for *E* = S and 2.447 Å for *E* = Se.

**Figure 6:**
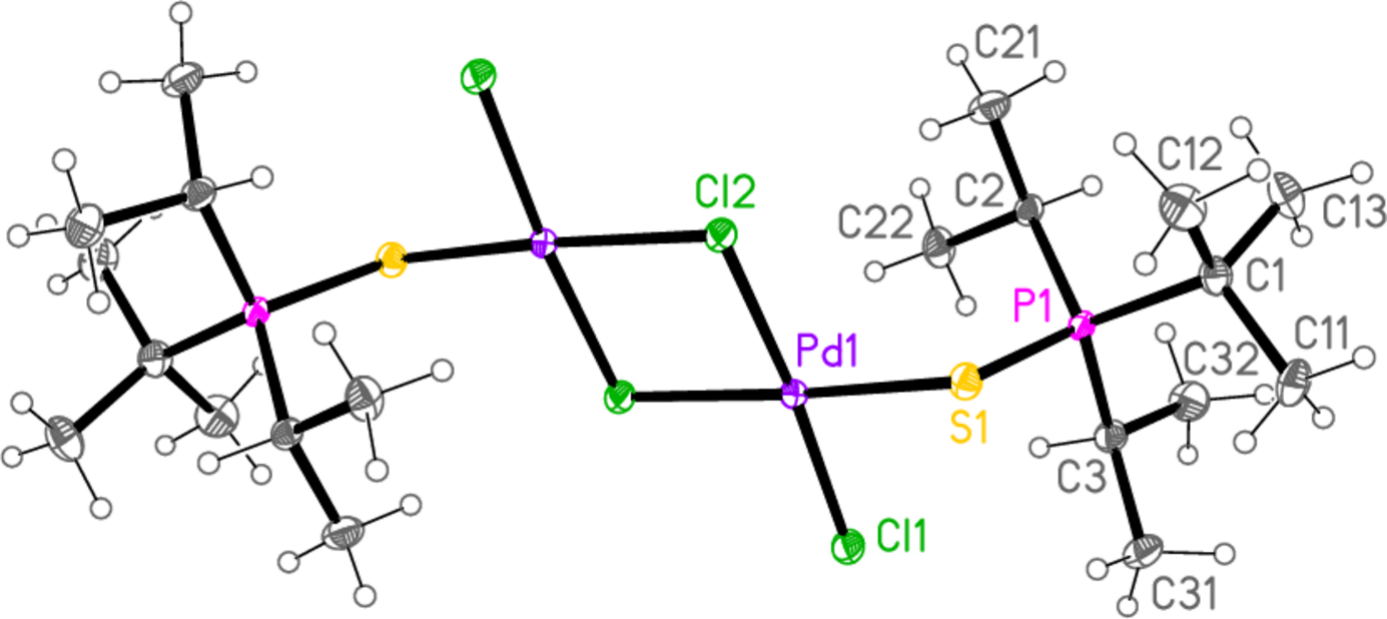
The mol­ecule of compound **6** in the crystal. Only the asymmetric unit is labelled.

The dinuclear complex **6** (Fig. 6 ▸) has the composition *L*_2_Pd_2_Cl_4_ rather than the expected *L*_2_PdCl_2_ and displays crystallographic inversion symmetry (with the inversion centre at the centre of the four-membered ring). The bonds to the bridging chlorine atom Cl2 in the central Pd_2_Cl_2_ ring are, as expected, longer than those to the terminal atom Cl1, with lengths of 2.3623 (6) and 2.3349 (5) Å for the former and 2.2799 (6) Å for the latter. The Pd1—S1 bond is, at 2.2882 (6) Å, shorter than the *M*—S bonds in **4** and **5**, reflecting the weaker *trans* influence of (bridging) chlorine compared to sulfur. The P1—S1—Pd1 angle of 107.34 (3)°, several degrees narrower than for **4** and **5**, underlines the highly variable nature of the P—E—M angles in these compounds. This compound shows the shortest intra­molecular H⋯M contact, namely H22*A*⋯Pd1 = 2.52 Å.

**Figure 7:**
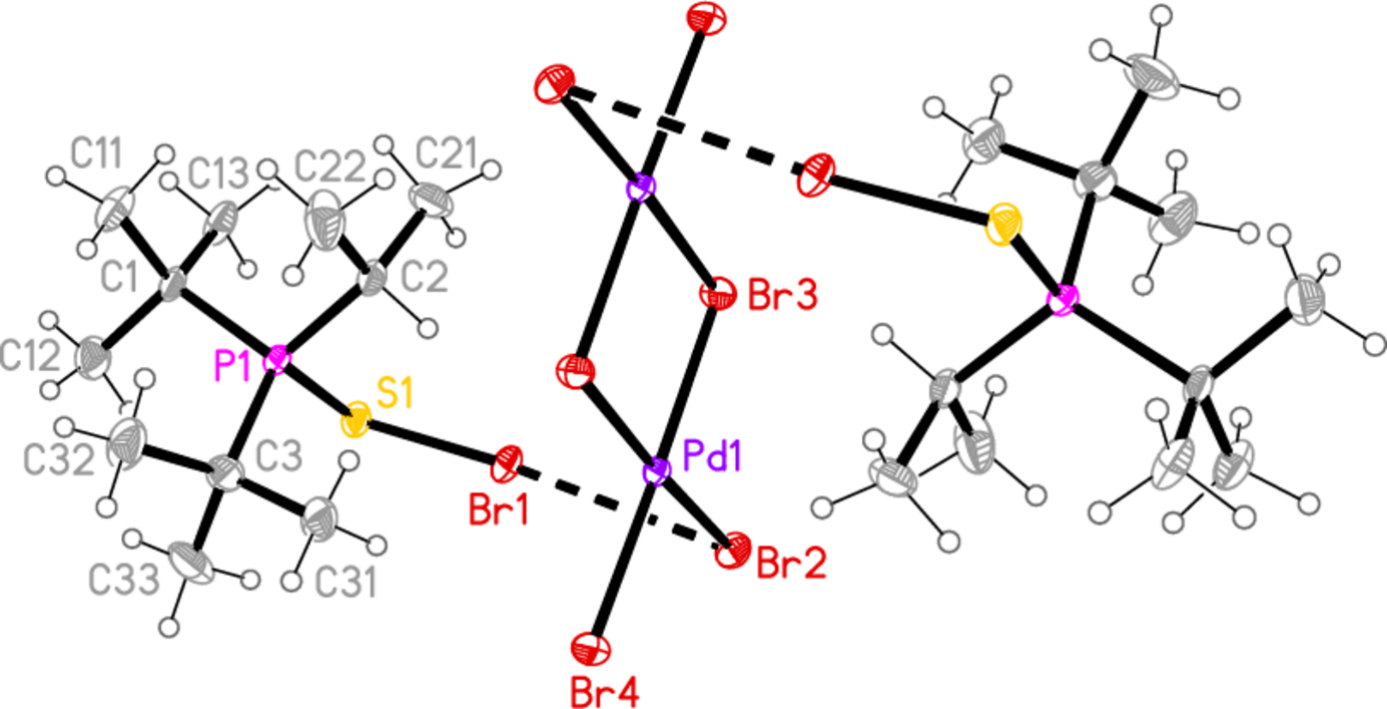
The structure of compound **7** in the crystal. Only the asymmetric unit is labelled. Only the major sites of the disordered methyl groups are shown. The dashed lines indicate short Br⋯Br contacts.

**Figure 8:**
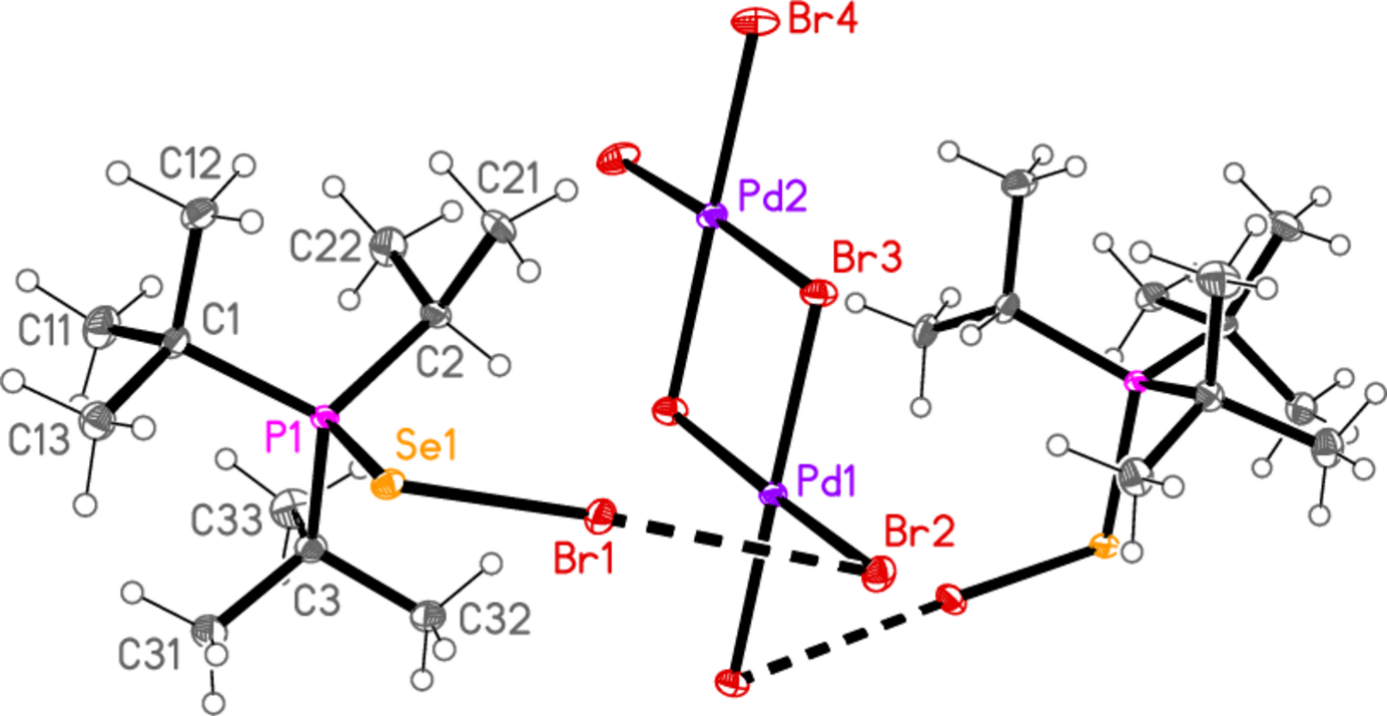
The structure of compound **8** in the crystal. Only the asymmetric unit is labelled. The dashed lines indicate short Br⋯Br contacts.

Compounds **7** and **8**, although differing only in the atom *E*, are not isotypic. The [Pd_2_Br_6_]^2−^ dianion of compound **7** (Fig. 7 ▸) displays crystallographic inversion symmetry, with the inversion centre at the centre of the four-membered ring, whereas the corresponding dianion of compound **8** (Fig. 8 ▸) shows crystallographic twofold symmetry, with both Pd atoms lying on the twofold axis at 0.5, *y*, 0.25. Both anions are essentially planar, with mean deviations of 0.006 and 0.032 Å, respectively. Again, the bonds to the bridging bromine atoms in the central ring are longer than those to the terminal atoms, with average lengths of 2.448 and 2.417 Å, respectively. The halochalcogenyltri­alkyl­phospho­nium cations have P—*E* and *E*—Br bond lengths [**7**: P1—S1 = 2.0941 (7), S1—Br1 = 2.2027 (14); **8**: P1—Se1 = 2.2505 (8), Se1—Br1 = 2.3310 (4) Å] that are closely similar to the average values (P—S = 2.095, P—Se = 2.248, S—Br = 2.200, Se—Br = 2.322 Å) for the same cations in the tetra­halogenidoaurate(III) salts, reported in Part 8 (Upmann *et al.*, 2024*c* ▸). The more variable P—*E*—Br angles, which correlated well with increasing steric bulk for the much more numerous gold derivatives, can best be compared to the derivatives with the same (^*t*^Bu_2_^i^PrP*E*Br)^+^ cations; P1—S1—Br1 in **7** = 103.52 (7), P1—Se1—Br1 in **8** = 100.30 (2)°, compared to their [AuBr_4_]^−^ salts with 104.48 (6) and 101.91 (6)°, respectively.

**Figure 9:**
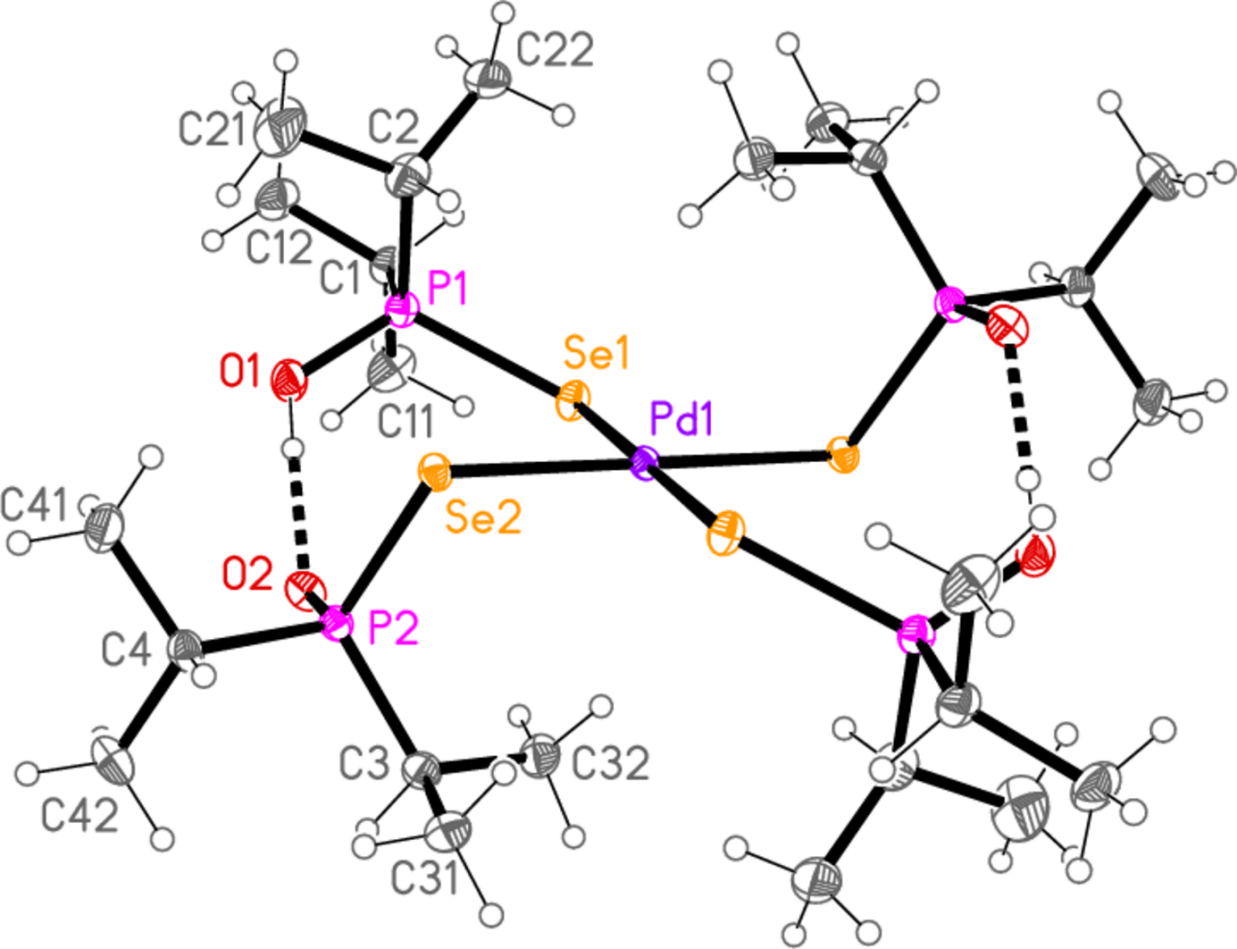
The mol­ecule of compound **9** in the crystal. Only one position of the disordered bridging hydrogen atom is shown. Dashed lines indicate hydrogen bonds.

**Figure 10:**
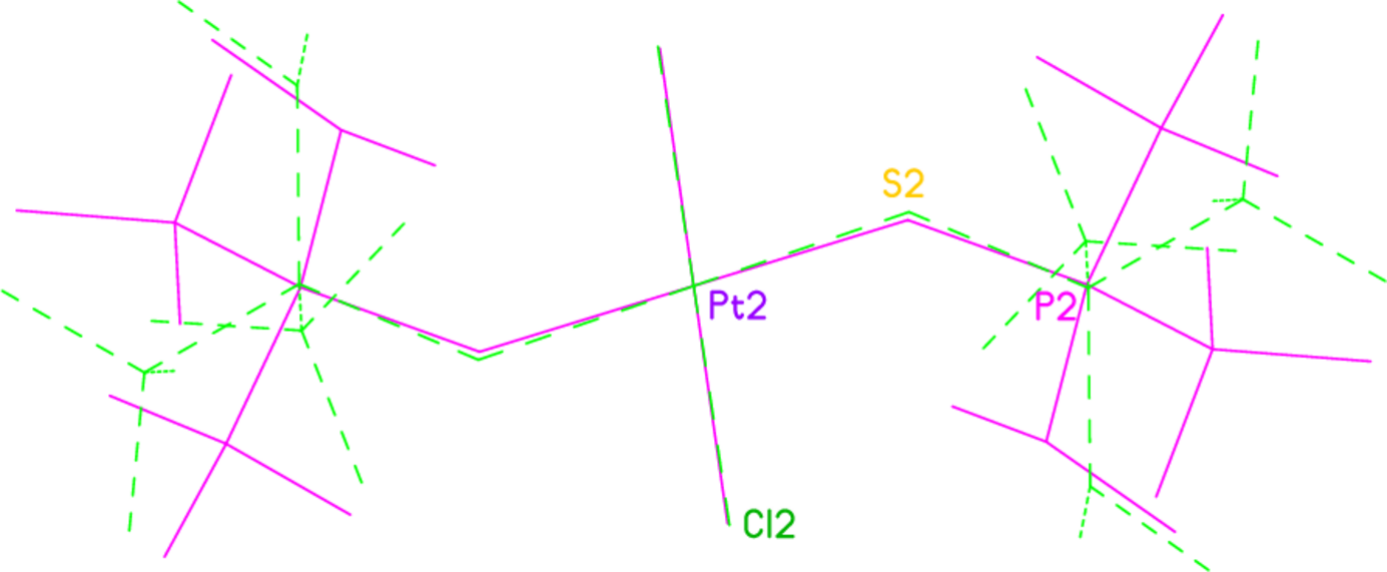
A least-squares fit of the two independent mol­ecules of compound **5**, showing the mutual rotation of the alkyl groups. Mol­ecule 1 is green with dashed bonds; mol­ecule 2 is violet. Fitted atoms are labelled; their symmetry-equivalent atoms were also fitted. Hydrogen atoms are omitted.

The mol­ecule of compound **9** (Fig. 9 ▸), which presumably arose under the influence of adventitious water, displays crystallographic inversion symmetry. As observed for **2** and **3** (see above), the two independent P—Se—Pd angles differ appreciably. The ‘half’ hydrogen atoms at O1 and O2 (see *Refinement*) are disordered; the oxygen atoms show no signs of disorder and the P—O bond lengths are effectively equal. The phospho­rus atoms are displaced to opposite sides of the coordination plane (defined by the atoms Pd1, Se1 and Se2), P1 by 0.8076 (5) and P2 by 1.9183 (5) Å. The torsion angle P1—Se1⋯Se2—P2 (omitting the Pd atom) is 83.83 (2)°. The short intra­molecular contact H32*A*⋯Pd1, 2.69 Å, is noteworthy.

## 3. Supra­molecular features

Tables 10 ▸–18 ▸ ▸ ▸ ▸ ▸ ▸ ▸ ▸ list short contacts that might be inter­preted as ‘weak’ hydrogen bonds; these include some borderline cases that are not further discussed, together with short intra­molecular contacts, which may be regarded as a result of the steric crowding, and include contacts of the type H⋯*M* that are as short as 2.52 Å. In the packing diagrams, the labelling denotes atoms of the asymmetric unit.

**Figure 11:**
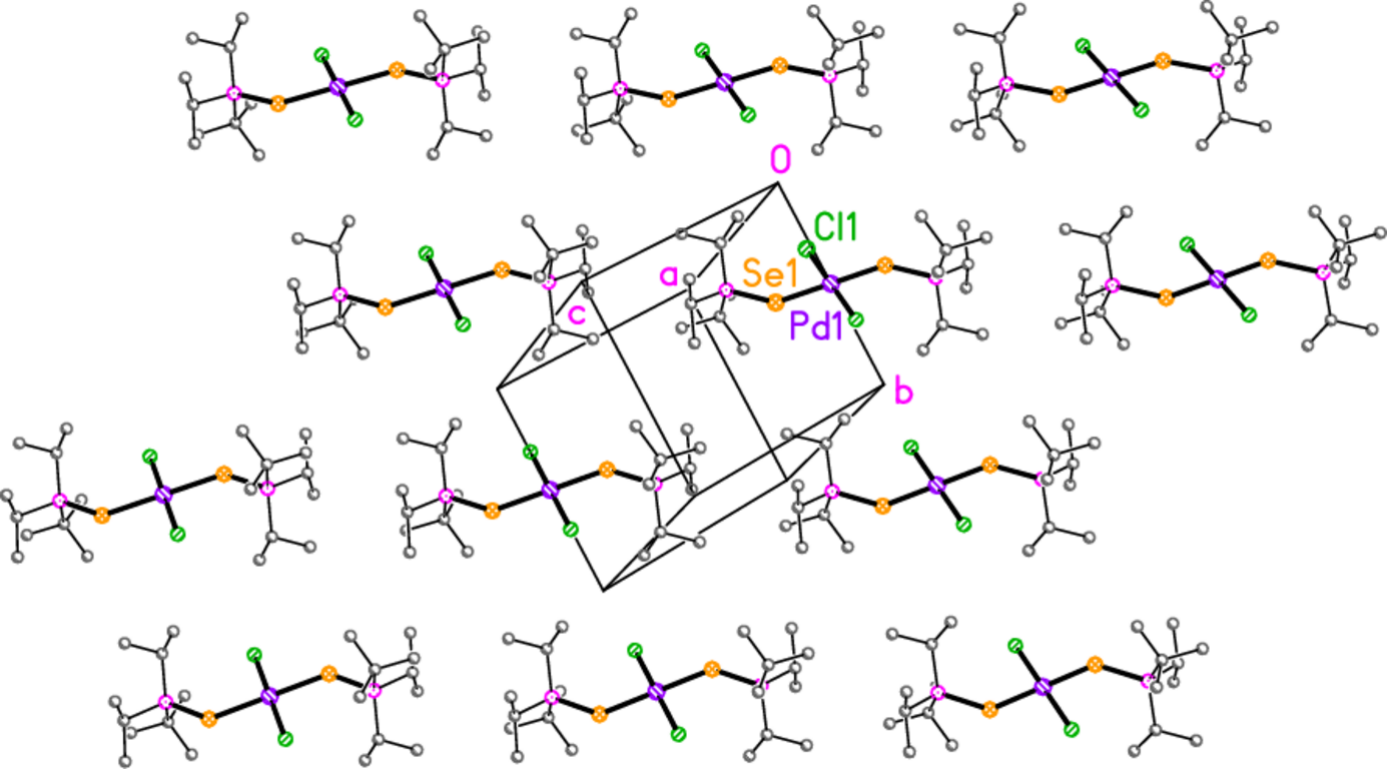
The packing of compound **1**, tentatively inter­preted as a layer of mol­ecules parallel to (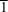
01). The view direction is perpendicular to the layer. All hydrogen atoms are omitted.

In marked contrast to the various series of gold compounds, structures **1**–**6** and **9** show few short inter­molecular contacts (*e.g.* H⋯*X* or *E*⋯*X*), presumably because of the increased steric effects of having two bulky ligands per mol­ecule rather than one. For compound **1**, for instance, the methine hydrogen atoms, which were generally prolific in forming short contacts in the gold complexes, only form one such contact, which is intra­molecular (H2⋯Cl1= 2.85 Å), and this is also true for several of the other structures. The intra­molecular contact H31⋯Cl1′ is shorter, at 2.70 Å. The program *XP* (Bruker, 1998 ▸) found no inter­molecular contacts shorter than (sum of atomic radii + 1.7 Å), using the ‘PACK 1.7’ command, which deliberately ignores H⋯H contacts; it is probable that the packing is determined by a large number of weak van der Waals contacts such as H⋯H. Fig. 11 ▸ shows a layer of mol­ecules parallel to (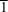
01), which can be inter­preted as consisting of chains of mol­ecules parallel to [1
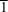
1].

**Figure 12:**
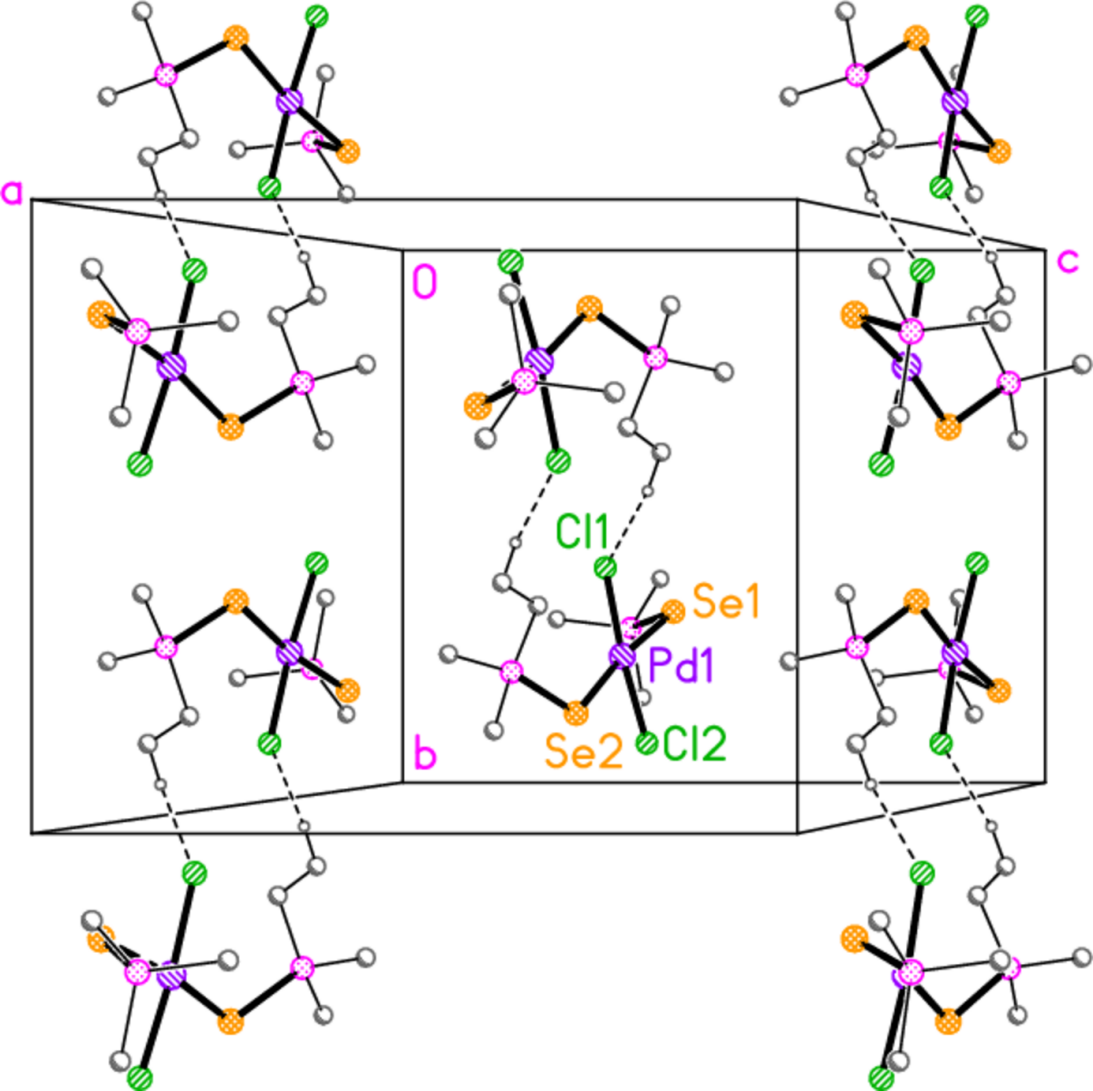
The packing of compound **2**, showing the formation of inversion-symmetric dimers *via* H⋯Cl contacts (dashed lines). The view direction is perpendicular to the *bc* plane.

**Figure 13:**
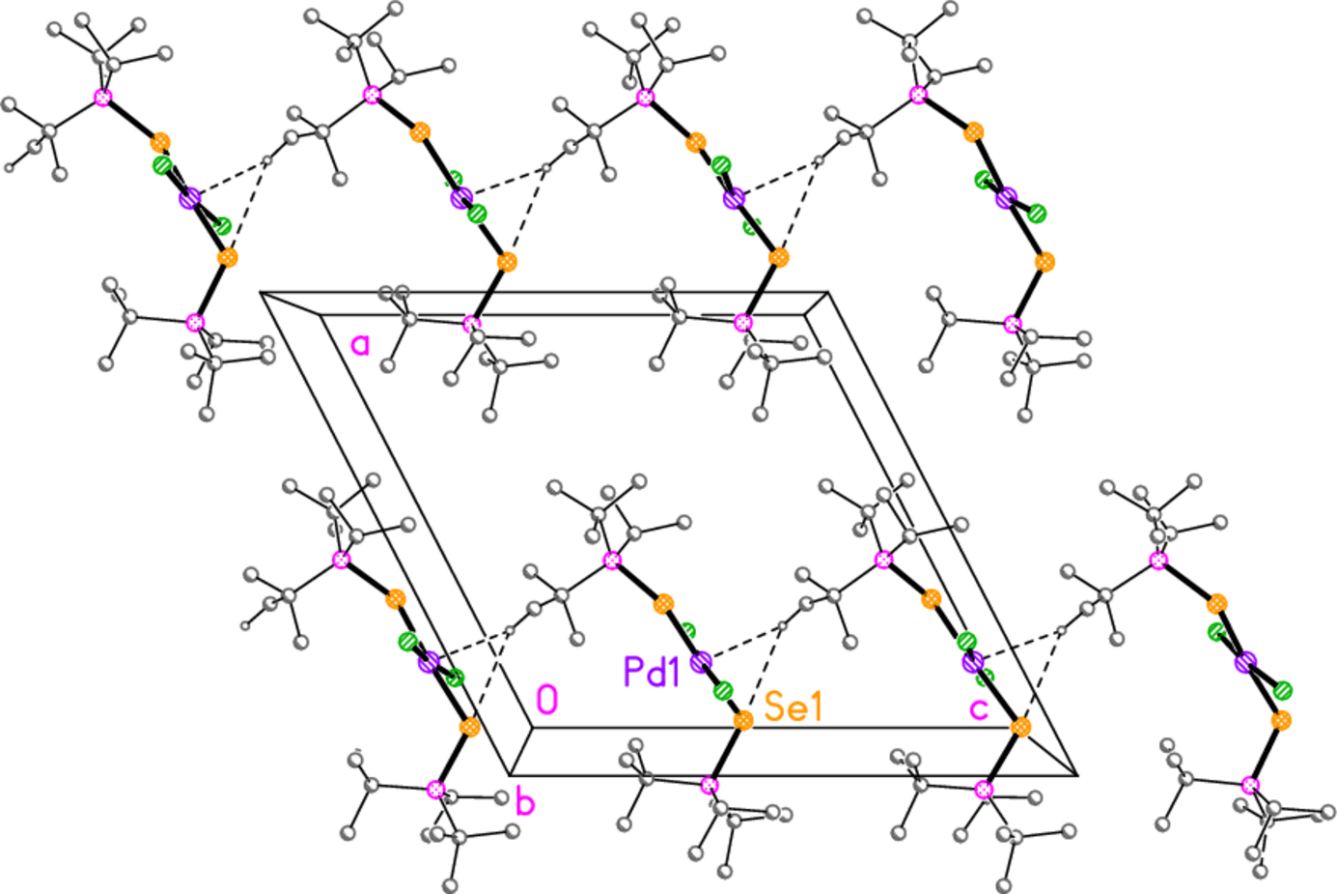
The packing of compound **2**, showing the short three-centre contacts H63*A*⋯(Pd1, Se1) (dashed lines). The view direction is parallel to the *ac* plane, and the region *y* ≃ 0.25 is depicted. Note that neighbouring mol­ecules are connected by the *c* glide operator and *not* by translation, despite their very similar orientation.

In compound **2**, the hydrogen bond H51*C*⋯Cl1(1 − *x*, 1 − *y*, 1 − *z*) links the mol­ecules to form inversion-symmetric dimers (Fig. 12 ▸). A further packing motif, the connection of mol­ecules by the three-centre contacts H63*A*⋯(Se1, Pd1) (*x*, 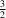 − *y*, −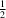 + *z*), leads to ribbons of mol­ecules parallel to the *c* axis (Fig. 13 ▸). The concept of hydrogen bonds H⋯*M*, where *M* is a noble metal, is well-established for *M* = Au (Schmidbaur *et al.*, 2014 ▸; Schmidbaur, 2019 ▸), but we are not aware of any systematic survey for *M* = Pd or Pt. Compound **3** is isotypic to **2** and the packing motifs are therefore analogous.

**Figure 14:**
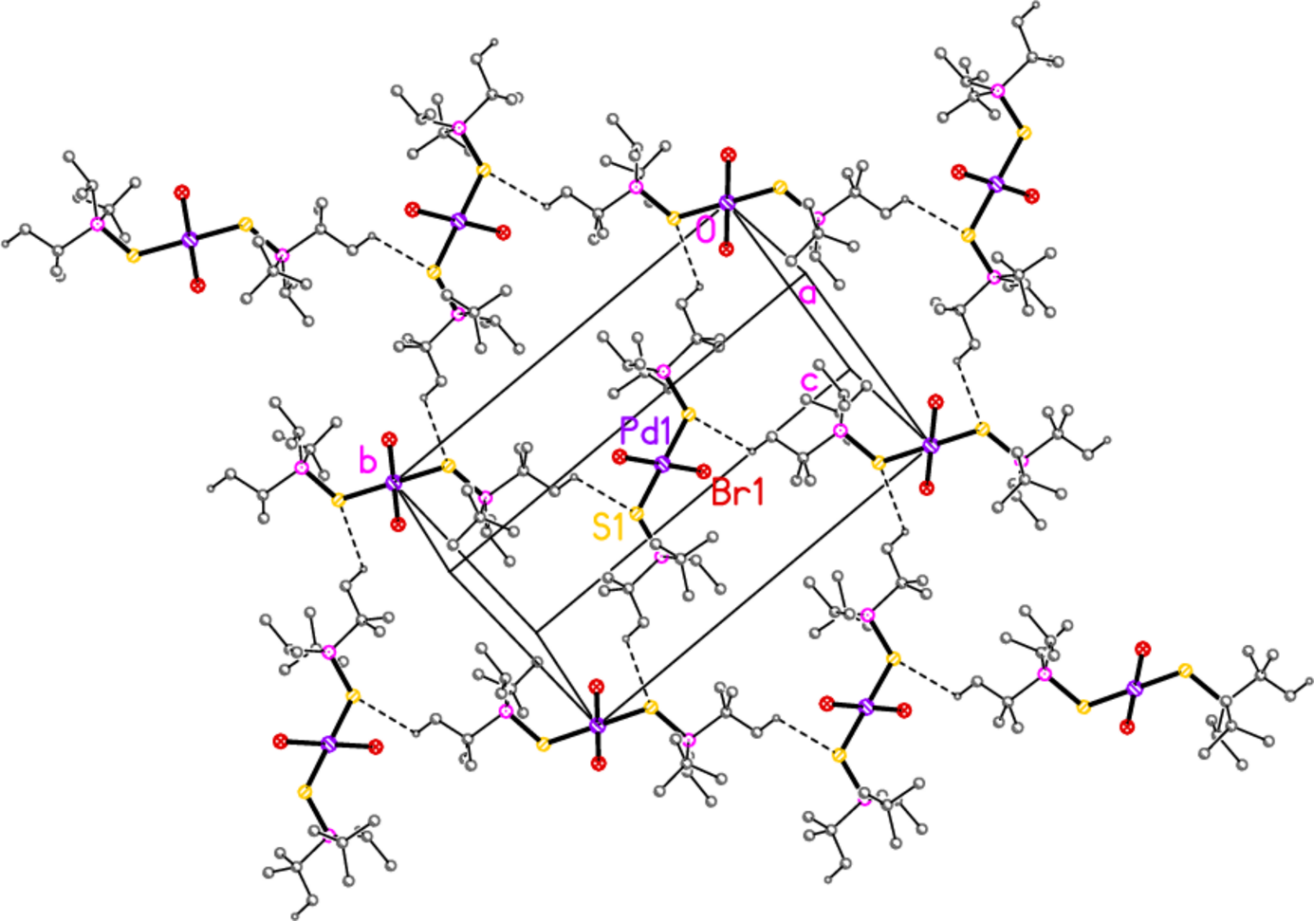
The packing of compound **4**, inter­preted as a layer structure parallel to (10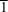) involving H⋯S inter­actions (dashed lines). The view direction is perpendicular to the layer.

For compound **4**, the packing involves no markedly short contacts, but may be inter­preted as a layer structure parallel to (10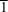) (Fig. 14 ▸) in which mol­ecules are connected by the borderline inter­action H12*A*⋯S1(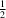 + *x*, 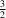 − *y*, 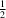 + *z*). The latter contact should perhaps be regarded as an aid to inter­pretation of the pattern rather than a definite inter­action, a caveat that applies to several of the structures described here.

**Figure 15:**
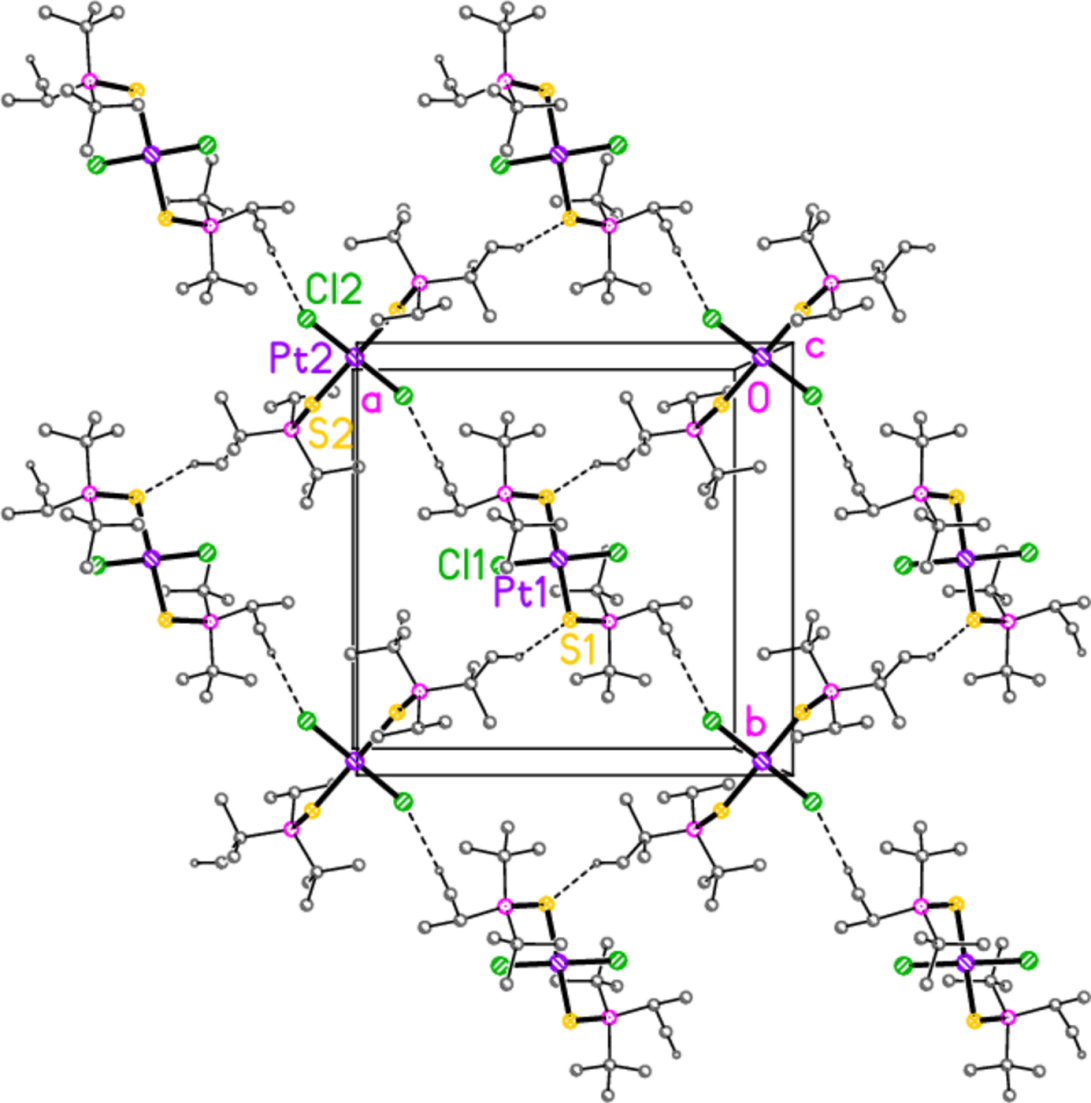
The packing of compound **5**, inter­preted as a layer structure parallel to the *ab* plane in the region *z* ≃ 0.5. Dashed lines indicate H⋯Cl and H⋯S contacts. The view direction is perpendicular to the layer.

For compound **5**, the contacts H32*A*⋯Cl2′(−1 + *x*, 1 + *y*, *z*) and the borderline H43*A*⋯S1(2 − *x*, 1 − *y*, 1 − *z*) combine to form layers parallel to the *ab* plane (Fig. 15 ▸).

**Figure 16:**
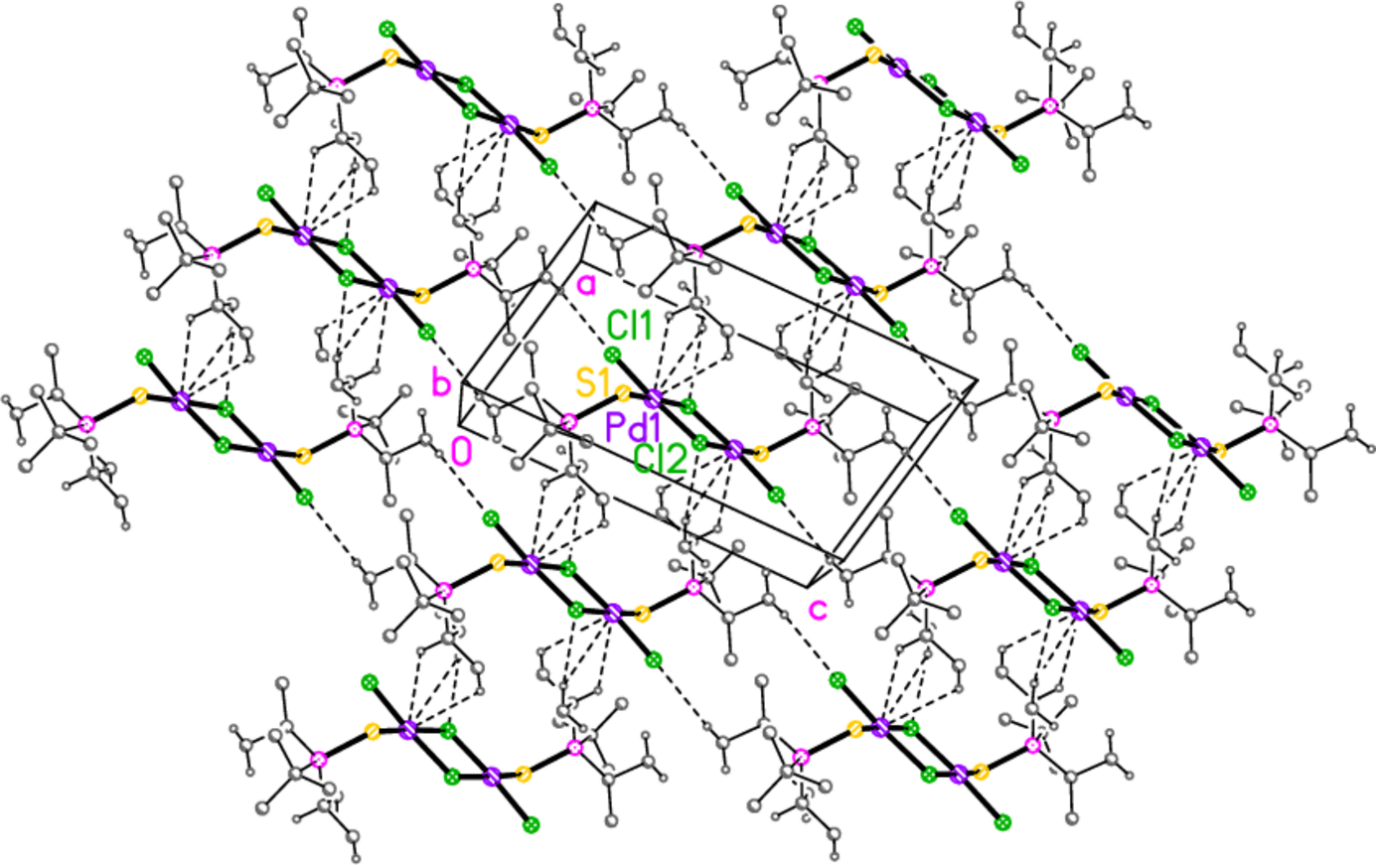
The packing of compound **6**, inter­preted as a layer structure parallel to the *ac* plane in the region *y* ≃ 0. Dashed lines indicate H⋯Cl and H⋯Pd contacts. The view direction is perpendicular to the layer.

Compound **6** may be inter­preted as a layer structure parallel to the *ac* plane (Fig. 16 ▸) involving the borderline contacts H32*C*⋯Cl1(1 − *x*, −*y*, −*z*) and H22*C*⋯Cl2(−1 + *x*, 1 + *y*, *z*) together with the trio of contacts (H2, H22*C*, H21*A*)⋯Pd1(−1 + *x*, *y*, *z*).

**Figure 17:**
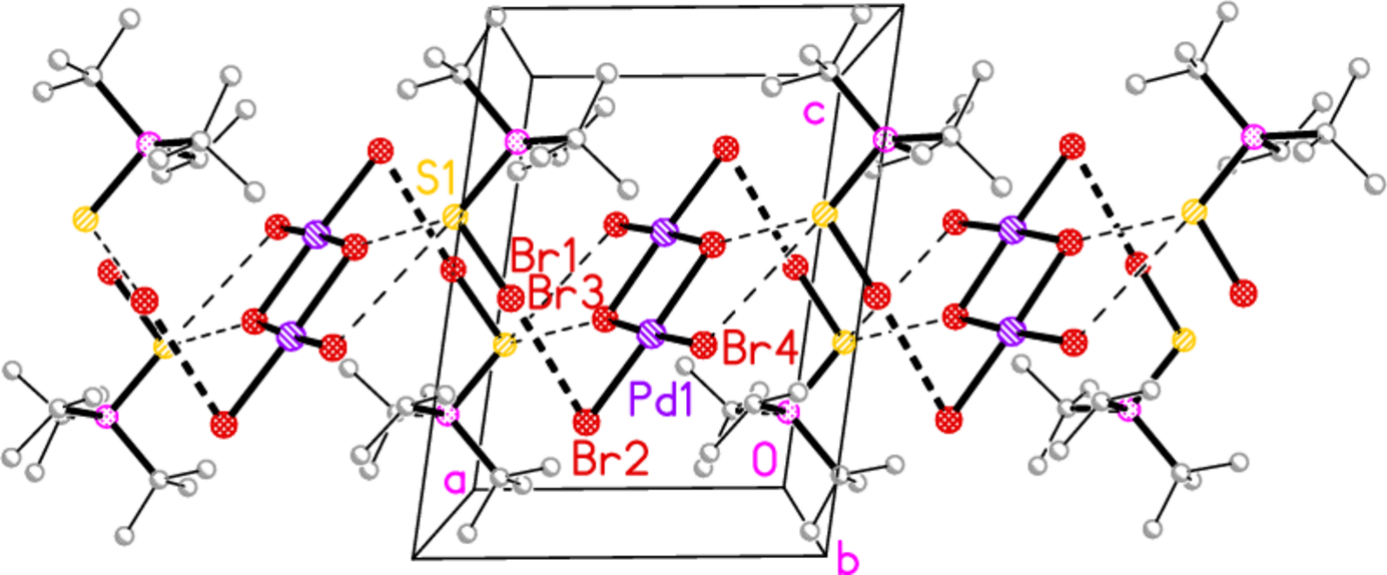
The packing of compound **7**, viewed parallel to the *b* axis in the region *y* ≃ 0.5. All hydrogen atoms are omitted. The dashed lines indicate Br⋯Br (thick) or Br⋯S (thin) contacts.

**Figure 18:**
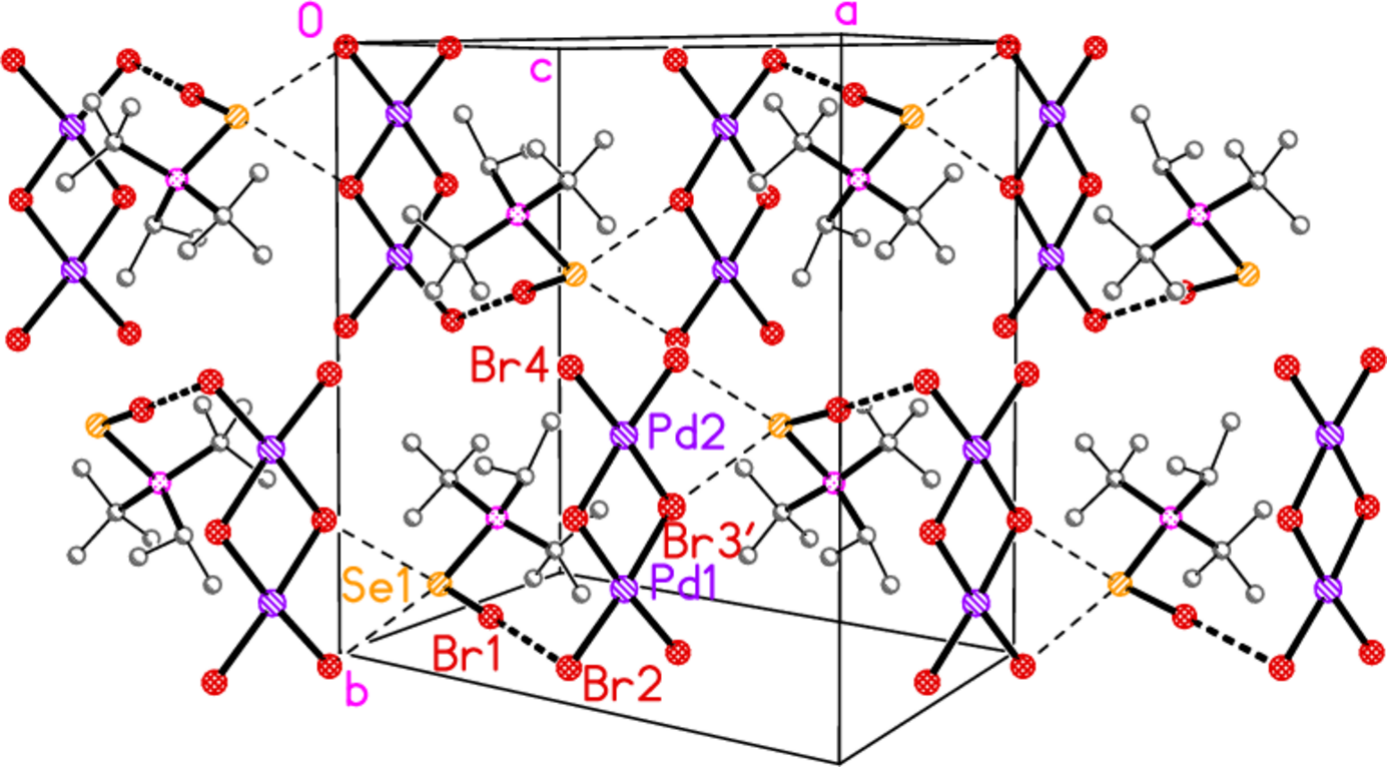
The packing of compound **8**, showing two ribbons of residues running horizontally, The view direction is approximately perpendicular to (10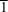), but was rotated slightly about the horizontal axis to minimize overlap of the ribbons. All hydrogen atoms are omitted. The dashed lines indicate Br⋯Br (thick) or Br⋯Se (thin) contacts. For clarity, the atom Br3 is represented by its equivalent Br3′.

In compound **7**, the cation and anion are connected by an extremely short halogen bond (for a review see Metrangolo *et al.*, 2008 ▸) Br1⋯Br2 of 3.2387 (7) Å, with a linear grouping S1—Br1⋯Br2 = 175.04 (3)° (Fig. 7 ▸), which is approximately perpendicular to the coordination plane of the metal, with Pd1—Br2⋯Br1 = 71.34 (2)°. Analogous halogen bonds were common, but not ubiquitous, features of the corresponding Au^III^ derivatives (*R*^1^*R*^2^*R*^3^P*EX*)[Au*X*_4_] (Upmann *et al.*, 2024*c* ▸). The ions are further linked by the contacts S1⋯Br3 (1 − *x*, 1 − *y*, 1 − *z*) 3.5463 (14) Å and the rather longer S1⋯Br4(1 + *x*, *y*, *z*) = 3.8042 (14) Å, with P1—S1⋯Br′ angles of 116.90 (6) and 173.51 (7)°, respectively, to produce ribbons of residues parallel to the *a* axis (Fig. 17 ▸). Despite the differing crystallographic symmetry, the packing of compound **8** is similar to that of **7**, with the halogen bond Br1⋯Br2 [3.2510 (5) Å, with Se1—Br1⋯Br2 = 176.81 (2) and Pd1—Br2⋯Br1 = 75.133 (10)°; see Fig. 8 ▸] and the Se⋯Br contacts Se1⋯Br4 and Se1⋯Br3 [3.5655 (5) and 3.6692 (5) Å, respectively, P—Se⋯Br′ angles of 167.14 (2) and 109.58 (2) Å, respectively, operator 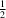 − *x*, 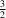 − *y*, −*z*] combining to produce ribbons parallel to [101] (Fig. 18 ▸).

**Figure 19:**
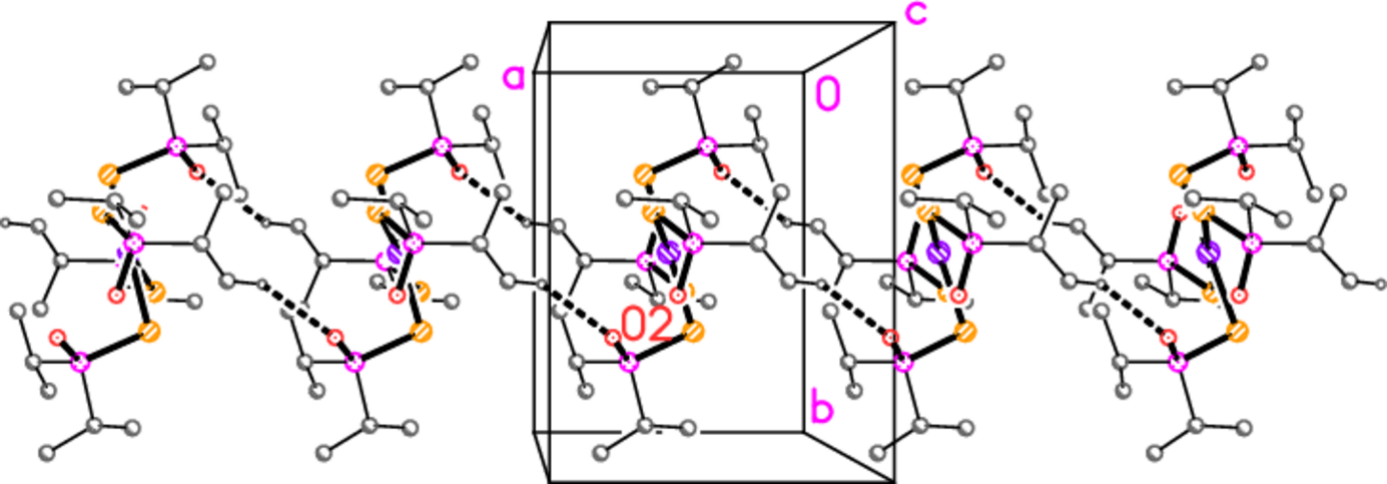
The packing of compound **9**, viewed perpendicular to the *ab* plane in the region *z* ≃ 0. Dashed lines indicate the short contact H21*A*⋯O2.

The packing of compound **9**, like those of **1**–**6**, is almost featureless. The main pattern involves ribbons parallel to the *a* axis *via* the ‘weak’ hydrogen bond H21*A*⋯O2′ (Fig. 19 ▸).

## 4. Database survey

The searches employed the routine ConQuest (Bruno *et al.*, 2002 ▸), part of Version 2024.1.0 of the Cambridge Structural Database (Groom *et al.*, 2016 ▸).

A search for compounds containing the moiety (C_3_P*E*)_2_*MX*_2_, with *E* = S or Se, *X* = any halogen, *M* = Pd or Pt, and coordination number 2 for *E*, gave seven hits, of which six were unique; all had *X* = Cl and all but one had *M* = Pd. Only two involved monodentate phosphane chalcogenide ligands (three were bidentate and one tridentate), namely *trans*-di­chlorido­bis­(tri-isobutyl phosphane sulfide)­palladium(II) (Rich­ardson, 1985 ▸; refcode COSWUC) and *trans*-bis­(di­ethyl­phenyl­phosphane sulfide)­dichlorido­palladium(II) (Satek *et al.*, 1975 ▸; EPPTPD). Both have crystallographic inversion symmetry, with Pd—S = 2.334 (1), Pd—Cl = 2.297 (1) and P—S = 2.014 (1) Å for the former and 2.350 (1), 2.302 (1) and 2.013 (2) Å, respectively, for the latter.

A search for the *M*_2_*X*_4_(*E=P*C_3_)_2_ core, as in **6**, gave only one hit, namely Pd_2_Cl_4_(S=PCy_2_Ar), where Cy = cyclo­hexyl and Ar is a 1,4-dimeth­oxy-3-tri­meth­oxy­phenyl-2-naphthyl group (Miroslaw *et al.*, 2023 ▸; ROGZEW). The bond lengths of the core are closely similar to those of **6**.

Finally, a search for the [Pd_2_Br_6_]^2−^ anion gave 22 hits with 24 independent anions. The terminal Pd—Br bond lengths were 2.369–2.438, av. 2.407 (11), and the bridging bonds were as expected significantly longer, at 2.423–2.513, av. 2.453 (13) Å.

## 5. Synthesis and crystallization

Full details of the preparations (including NMR data) are given in the PhD thesis of Upmann (2015 ▸). Here we present three representative syntheses.

Compound **2**: Palladium dichloride (95 mg, 0.5 mmol) was refluxed for 1 h in 25 mL of aceto­nitrile to give an orange solution. After cooling to r.t., ^*t*^Bu_2_^i^PrPSe (286 mg, 1.0 mmol) was added, causing an immediate colour change to reddish-brown. After stirring overnight, the solvent was removed *in vacuo*, the brown residue was washed with *n*-pentane (3 × 3 mL) and diethyl ether (2 × 3 mL) and dried *in vacuo*. The product was recrystallized from di­chloro­methane/*n*-pentane. ^31^P NMR (81 MHz, CDCl_3_): δ = 79.93 (singlet with P—Se satellites, *J*_PSe_ = 577 Hz).

Compound **6**: Palladium dichloride (454 mg, 2.6 mmol) was refluxed for 1 h in 100 mL of aceto­nitrile to give an orange solution. After cooling to r.t., ^*t*^Bu^i^Pr_2_PS (1.057 g, 5.2 mmol) was added, causing an immediate colour change to brown. After stirring overnight, the solvent was removed *in vacuo*, the brown residue was washed with *n*-pentane (2 × 5 mL) and diethyl ether (2 × 5 mL) and dried *in vacuo*. ^31^P NMR (81 MHz, CDCl_3_): δ = 81.54 (*s*).

Compound **8**: Compound **3** (103 mg, 0.1 mmol) was dissolved in 5 mL of di­chloro­methane. The solution was carefully overlayered with *n*-pentane, and two drops of elemental bromine were immediately added. Red crystals of **8** formed overnight. Elemental analysis: calculated: C 19.06, H 3.63. Found: C 18.79, H 3.77%. ^31^P NMR (81 MHz, CDCl_3_): δ = 83.77 (*s*). The solubility was too poor to detect P—Se coupling.

## 6. Refinement

Details of the measurements and refinements are given in Table 19 ▸. Structures were refined anisotropically on *F*^2^. Methine hydrogens were included at calculated positions and refined using a riding model with C—H = 1.00 Å and *U*_iso_(H) = 1.2 × *U*_eq_(C). Methyl groups were refined, using the command ‘AFIX 137’, as idealized rigid groups allowed to rotate but not tip, with C—H = 0.98 Å, H—C—H = 109.5° and *U*_iso_(H) = 1.5 × *U*_eq_(C). This procedure, relying as it does on the location of electron-density maxima corresponding to the H-atom sites, is less reliable for heavy-atom structures, so that any postulated hydrogen bonds involving methyl hydrogen atoms (especially for the disordered methyl groups of compound **7**) should be inter­preted with caution; however, clear maxima in the electron density were generally found.

*Exceptions and special features*. For compound **6**, two reflections with Δ/σ > 6 were omitted from the refinement; for compound **7**, three reflections with Δ/σ = 7–13 were similarly omitted. For compound **7**, the *tert*-butyl groups at C1 and C3 are disordered over two positions; the occupation factors of the isotropically refined minor components were 0.19 (1) at C1 and 0.20 (3) at C3. Appropriate restraints were applied to improve refinement stability; additionally, the isotropic *U* value of the minor component of C31 had to be fixed to prevent it becoming negative. The dimensions of disordered groups (especially the minor components) should always be inter­preted with caution. Only the major components were considered for the discussion and the figures. For compound **9**, the hydrogen atom of the O—H⋯O moiety was refined on two alternative, half-occupied positions (the occupations were fixed; refining them led to values within 1σ of 0.5). There is no evidence (on the basis of *U* values or residual electron density) that the corresponding oxygen atoms are disordered, and the P—O bond lengths are effectively equal, so that localized P—O and P=O bonds are unlikely.

## 7. Some comments on the SFAC command

Users of *SHELXL* will be familiar with the SFAC command, which defines the element types (and implicitly the scattering factors) to be used for each atom in the refinement. Thus the command ‘SFAC C H P SE BR PD’ defines carbon to be element type 1, hydrogen 2, phospho­rus 3, *etc*., and these numbers are given explicitly for each atom in the refinement (coming immediately after the atom name), *e.g.*

BR1 5 0.275182 0.581682 0.432225 11.00000 0.01922 0.01540 0.03417 − 0.00115 0.01030 0.00275

where the atom type ‘BR’ is the fifth element of the SFAC command (in this section, long lines of computer text have been split into more than one line).

The commonest convention for a standard order of SFAC elements is: C, then H, then other elements in order of atomic number. However, another possibility is: C, then H, then other elements alphabetically. Clearly, the refinement results will be the same in both cases, as long as the SFAC element numbers are given correctly for each atom. The user can change the SFAC order if required, making sure to change the SFAC numbers accordingly; the obvious danger is that, if errors are made, atoms may be refined with the wrong scattering factors. This is usually recognized easily, because the results will be entirely, and often disastrously, wrong (*e.g.* in terms of divergent refinement, high *R* factors, impossibly high or low *U* values, or major features in the residual electron density). A typical example would be an amine complex of gold, for which the third SFAC element would usually be N according to atomic number but Au alphabetically; erroneously using the scattering factors of nitro­gen to refine a gold atom, or *vice versa*, would result in nonsense, although the program *SHELXL* would not give an explicit error message.

In this age of automation, SFAC commands are generally set automatically by the program systems. Thus the Rigaku OD *CrysAlis PRO* (Rigaku, OD, 2012 ▸) system generates an INS file with an SFAC command corresponding to the ‘atomic number’ option. However, the *Autochem* option, which solves and refines the structure automatically during and after the data collection, using the *Olex2* platform of *SHELXL* (Dolomanov *et al.*, 2009 ▸), employs the ‘alphabetic’ option (in the version 1.171.43.143a that we currently use). A common first step in refining a structure is thus to extract the atom information from the *Olex2* RES file, edit it into the INS file and *change the SFAC command or the element type numbers appropriately* (if necessary; for many organic structures, *e.g.* those containing only C, H, N, O, P and S, there is no difference between the two options). Even experienced users occasionally forget to do this, but no permanent harm is done if the error is immediately recognized and corrected. *Note added during finalization of the manuscript*: Rigaku OD has informed us that the SFAC commands will be made consistent in the next version of the program, using the IUPAC recommendation for chemical formulae (Connelly & Damhus, 2005 ▸), the ‘alphabetic’ option, which was originally suggested by Hill (1900 ▸).

The structures reported here were determined some years ago, in an era where SFAC commands were often set by hand, and were re-refined for publication, using the most recent version of *SHELXL*. When preparing the structure of compound **3** for publication, a curious feature was noticed in the generation of both the figures and the tables; for elements with two letters in the atom symbol, the second letter remained a capital, although both *XP* (Bruker, 1998 ▸) and the tables program CIFTAB (as implemented in various *SHELX* platforms) usually convert the second letter automatically to lower case.

*checkCIF* (Spek, 2020 ▸ and references therein) gave no serious alerts of the type A or B; the solution to the conundrum was found in the list of ‘less serious’ alerts of type G:

PLAT017_ALERT_1_G Check Scattering Type Consistency of BR1 as SE

PLAT017_ALERT_1_G Check Scattering Type Consistency of BR2 as SE

PLAT017_ALERT_1_G Check Scattering Type Consistency of SE1 as BR

PLAT017_ALERT_1_G Check Scattering Type Consistency of SE2 as BR

These were generated because of corresponding inconsistencies in the CIF atom sites:

BR1 **Se** 0.27518 (3) 0.58168 (3) 0.43222 (3) 0.02212 (11) Uani 1 1 d . . . . .

BR2 **Se** 0.17265 (3) 0.91841 (3) 0.45811 (3) 0.01627 (10) Uani 1 1 d . . . . .

SE1 **Br** 0.08503 (3) 0.67520 (3) 0.45392 (3) 0.01664 (10) Uani 1 1 d . . . .

SE2 **Br** 0.35224 (3) 0.84539 (3) 0.42599 (3) 0.02206 (11) Uani 1 1 d . . . . .

The CIF atom list contains the explicit element symbols rather than the SFAC numbers, and it can thus be seen that the bromine atoms had been refined as selenium and *vice versa*, because the wrong SFAC numbers had been used in the refinement. Because the scattering factors of the two atom types, with atomic numbers 34 and 35, are not wildly different, the usual symptoms were not as obvious. The SFAC numbers were corrected and the refinement successfully completed. The *R* value thereby decreased only slightly (by *ca*. 0.2%), as did the residual electron density.

We conclude: (1) Even experienced users can make mistakes in the use of SFAC. (2) These errors are flagged by *checkCIF*, but only as ‘ALERT G’ (we feel that the severity should be upgraded to ‘ALERT B’ at least). *Note added during finalization of the manuscript*: The author of *checkCIF*, Professor A. L. Spek, has informed us that this change will soon be implemented. (3) Authors should check not only the serious A and B alerts, but also the ‘less serious’ alerts C and G; authors (and we include ourselves here!) have a natural tendency to screen the latter lists less conscientiously. (4) For reasons about which we do not speculate, such errors may be indicated by atom symbols with two capital letters when using *XP* or CIFTAB.

## Supplementary Material

Crystal structure: contains datablock(s) 1, 2, 3, 4, 5, 6, 7, 8, 9, global. DOI: 10.1107/S2056989025000805/yz2063sup1.cif

Structure factors: contains datablock(s) 1. DOI: 10.1107/S2056989025000805/yz20631sup2.hkl

Structure factors: contains datablock(s) 2. DOI: 10.1107/S2056989025000805/yz20632sup3.hkl

Structure factors: contains datablock(s) 3. DOI: 10.1107/S2056989025000805/yz20633sup4.hkl

Structure factors: contains datablock(s) 4. DOI: 10.1107/S2056989025000805/yz20634sup5.hkl

Structure factors: contains datablock(s) 5. DOI: 10.1107/S2056989025000805/yz20635sup6.hkl

Structure factors: contains datablock(s) 6. DOI: 10.1107/S2056989025000805/yz20636sup7.hkl

Structure factors: contains datablock(s) 7. DOI: 10.1107/S2056989025000805/yz20637sup8.hkl

Structure factors: contains datablock(s) 8. DOI: 10.1107/S2056989025000805/yz20638sup9.hkl

Structure factors: contains datablock(s) 9. DOI: 10.1107/S2056989025000805/yz20639sup10.hkl

CCDC references: 2420224, 2182097, 2420223, 2182096, 2182118, 2182120, 2182108, 2182092, 2182093

Additional supporting information:  crystallographic information; 3D view; checkCIF report

## Figures and Tables

**Figure 2:**
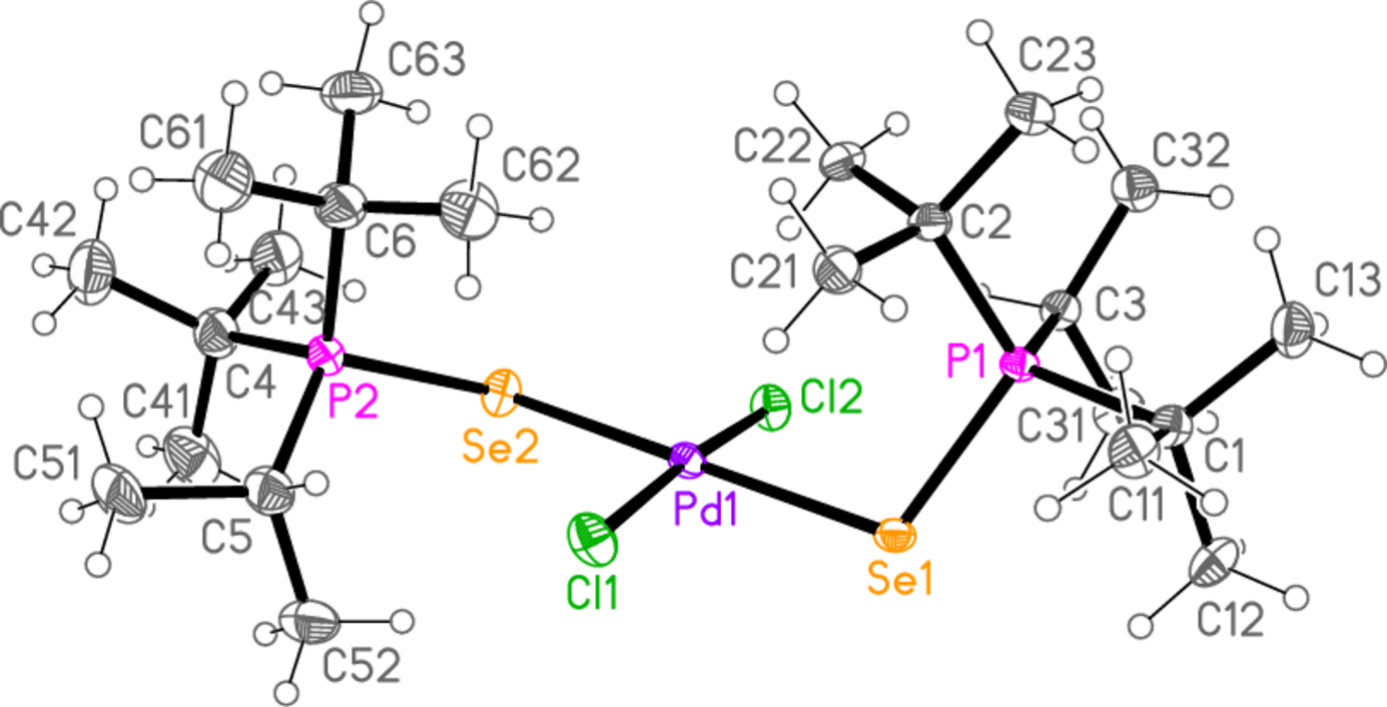
The mol­ecule of compound **2** in the crystal.

**Figure 3:**
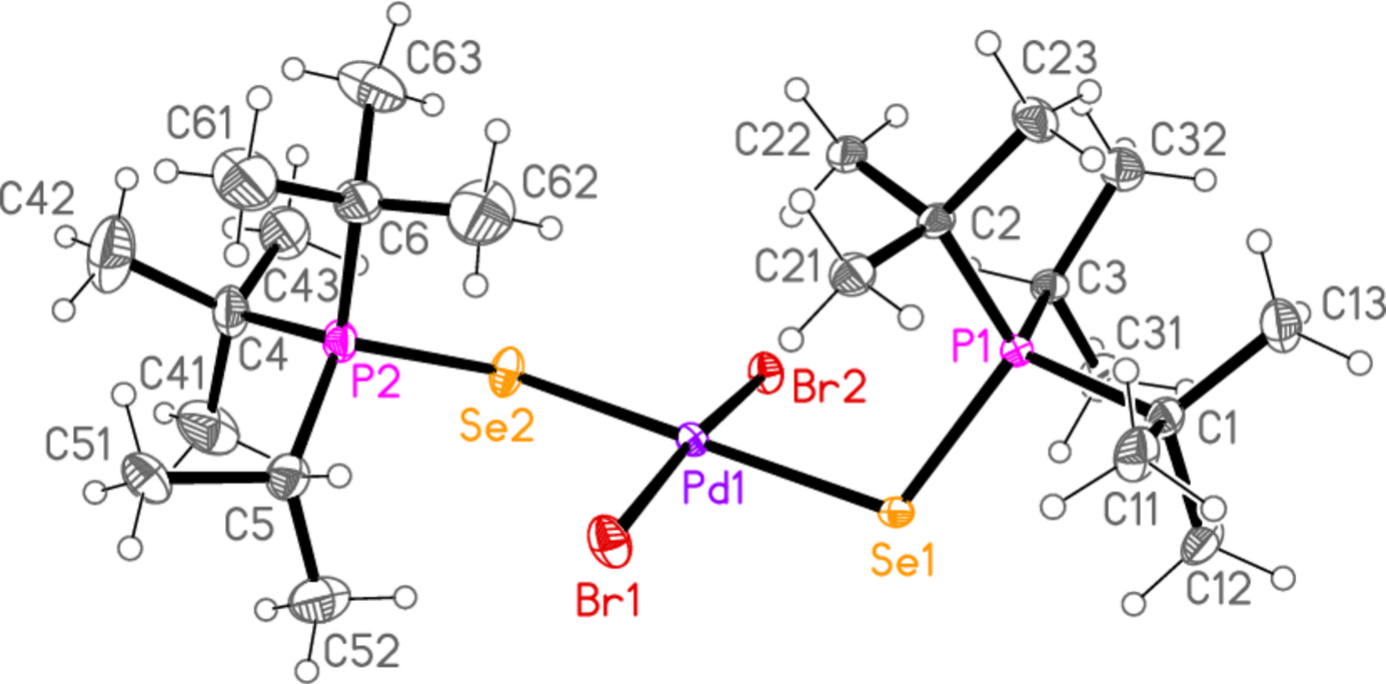
The mol­ecule of compound **3** in the crystal.

**Figure 4:**
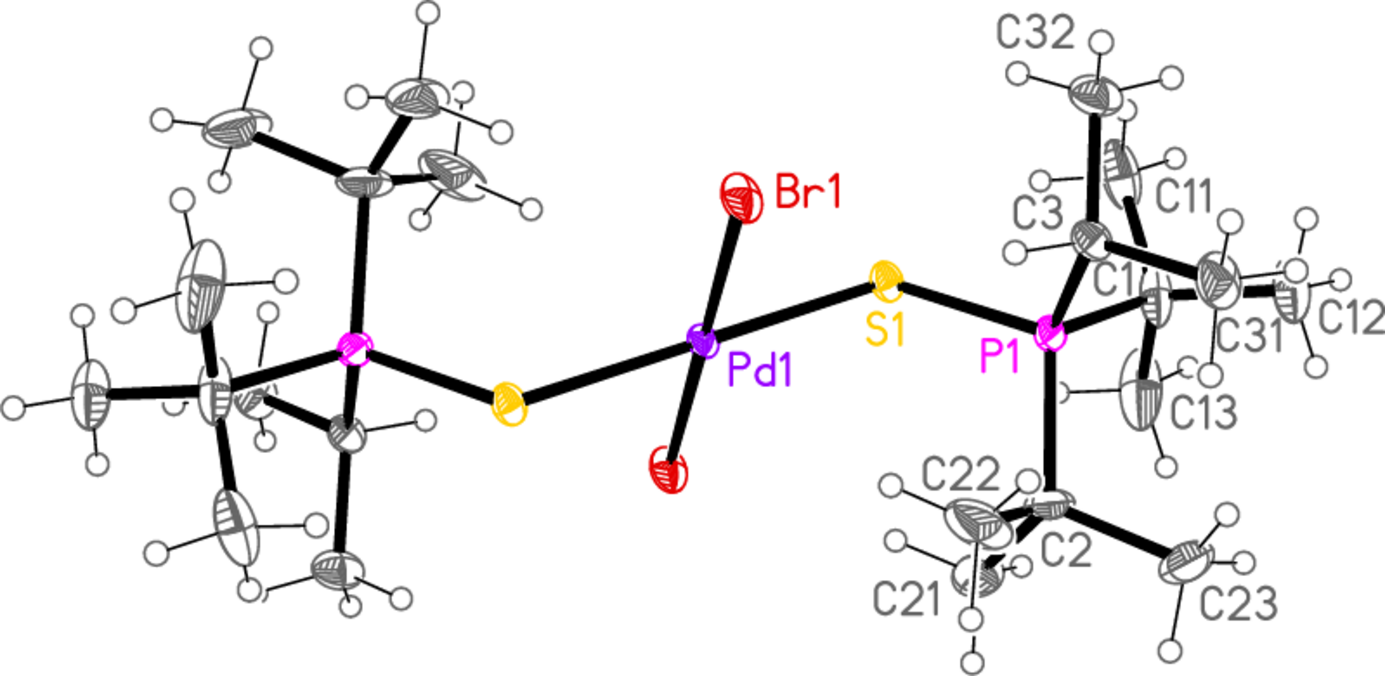
The mol­ecule of compound **4** in the crystal. Only the asymmetric unit is labelled.

**Figure 5:**
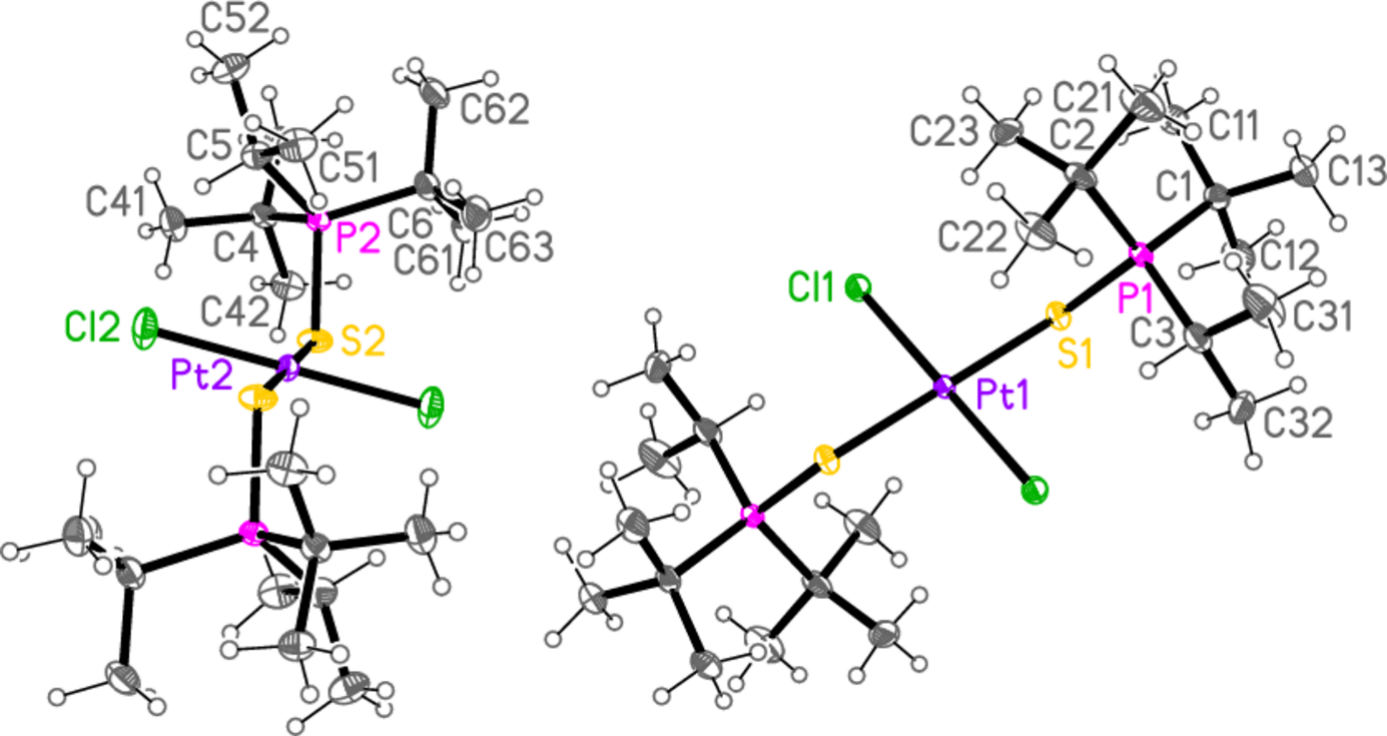
The two independent mol­ecules of compound **5** in the crystal. Each has inversion symmetry; only the asymmetric unit is labelled.

**Table 1:** Selected geometric parameters (Å, °) for **1**[Chem scheme1]

Pd1—Cl1	2.3099 (8)	Se1—P1	2.1881 (9)
Pd1—Se1	2.4322 (4)		
			
Cl1—Pd1—Se1^i^	81.37 (2)	C2—P1—Se1	114.92 (11)
Cl1—Pd1—Se1	98.63 (2)	C1—P1—Se1	102.53 (11)
Se1^i^—Pd1—Se1	180.0	C3—P1—Se1	114.48 (12)
P1—Se1—Pd1	116.92 (3)		

Symmetry code: (i) 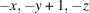.

**Table 2:** Selected geometric parameters (Å, °) for **2**[Chem scheme1]

Pd1—Cl1	2.3100 (7)	Pd1—Se2	2.4573 (4)
Pd1—Cl2	2.3169 (7)	Se1—P1	2.1870 (8)
Pd1—Se1	2.4411 (4)	Se2—P2	2.1936 (8)
			
Cl1—Pd1—Cl2	174.61 (3)	P1—Se1—Pd1	107.78 (2)
Cl1—Pd1—Se1	86.97 (2)	P2—Se2—Pd1	117.93 (3)
Cl2—Pd1—Se1	92.46 (2)	C3—P1—Se1	109.18 (10)
Cl1—Pd1—Se2	99.50 (2)	C2—P1—Se1	111.22 (9)
Cl2—Pd1—Se2	80.81 (2)	C1—P1—Se1	103.40 (10)
Se1—Pd1—Se2	172.772 (14)		

**Table 3:** Selected geometric parameters (Å, °) for **3**[Chem scheme1]

Pd1—Br1	2.4395 (5)	Se1—P1	2.1890 (11)
Pd1—Se1	2.4416 (5)	Se2—P2	2.1935 (12)
Pd1—Br2	2.4503 (5)	P1—C3	1.847 (4)
Pd1—Se2	2.4628 (5)		
			
Br1—Pd1—Se1	86.332 (18)	P1—Se1—Pd1	108.83 (3)
Br1—Pd1—Br2	172.66 (2)	P2—Se2—Pd1	119.30 (4)
Se1—Pd1—Br2	92.395 (17)	C3—P1—Se1	109.36 (14)
Br1—Pd1—Se2	100.149 (18)	C2—P1—Se1	111.31 (13)
Se1—Pd1—Se2	172.54 (2)	C1—P1—Se1	103.26 (14)
Br2—Pd1—Se2	80.706 (17)		

**Table 4:** Selected geometric parameters (Å, °) for **4**[Chem scheme1]

Pd1—S1	2.3317 (6)	S1—P1	2.0202 (10)
Pd1—Br1	2.4501 (3)		
			
S1^i^—Pd1—S1	180.0	P1—S1—Pd1	117.53 (4)
S1—Pd1—Br1	91.133 (17)	C3—P1—S1	111.18 (10)
S1—Pd1—Br1^i^	88.867 (17)	C2—P1—S1	111.32 (11)
Br1—Pd1—Br1^i^	180.0	C1—P1—S1	101.95 (11)

Symmetry code: (i) 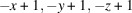.

**Table 5:** Selected geometric parameters (Å, °) for **5**[Chem scheme1]

Pt1—Cl1	2.3129 (7)	Pt2—S2	2.3278 (7)
Pt1—S1	2.3369 (7)	S1—P1	2.0322 (10)
Pt2—Cl2	2.3070 (7)	S2—P2	2.0323 (10)
			
Cl1—Pt1—Cl1^i^	180.0	P1—S1—Pt1	113.49 (3)
Cl1—Pt1—S1	87.04 (2)	P2—S2—Pt2	113.27 (4)
Cl1—Pt1—S1^i^	92.96 (2)	C3—P1—S1	108.40 (10)
S1—Pt1—S1^i^	180.0	C2—P1—S1	111.07 (10)
Cl2—Pt2—Cl2^ii^	180.0	C1—P1—S1	104.54 (10)
Cl2—Pt2—S2^ii^	89.24 (3)	C5—P2—S2	110.77 (10)
Cl2—Pt2—S2	90.75 (3)	C6—P2—S2	109.37 (10)
S2^ii^—Pt2—S2	180.0	C4—P2—S2	104.01 (10)

Symmetry codes: (i) 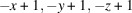; (ii) 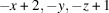.

**Table 6:** Selected geometric parameters (Å, °) for **6**[Chem scheme1]

Pd1—Cl1	2.2799 (6)	Pd1—Cl2^i^	2.3623 (6)
Pd1—S1	2.2882 (6)	S1—P1	2.0350 (7)
Pd1—Cl2	2.3349 (5)		
			
Cl1—Pd1—S1	93.95 (2)	Pd1—Cl2—Pd1^i^	94.46 (2)
Cl1—Pd1—Cl2	175.53 (2)	P1—S1—Pd1	107.34 (3)
S1—Pd1—Cl2	89.38 (2)	C3—P1—S1	110.54 (7)
Cl1—Pd1—Cl2^i^	91.03 (2)	C2—P1—S1	113.53 (7)
S1—Pd1—Cl2^i^	174.586 (19)	C1—P1—S1	105.38 (8)
Cl2—Pd1—Cl2^i^	85.54 (2)		

Symmetry code: (i) 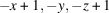.

**Table 7:** Selected geometric parameters (Å, °) for **7**[Chem scheme1]

Br1—S1	2.2027 (14)	Pd1—Br2	2.4199 (6)
Br1—Br2	3.2387 (7)	Pd1—Br3^i^	2.4447 (6)
P1—S1	2.0941 (18)	Pd1—Br3	2.4514 (6)
Pd1—Br4	2.4131 (6)		
			
S1—Br1—Br2	175.04 (4)	Br2—Pd1—Br3^i^	176.25 (2)
C2—P1—S1	107.50 (17)	Br4—Pd1—Br3	177.07 (2)
C1—P1—S1	100.83 (16)	Br2—Pd1—Br3	91.29 (2)
C3—P1—S1	109.19 (17)	Br3^i^—Pd1—Br3	85.00 (2)
P1—S1—Br1	103.52 (7)	Pd1—Br2—Br1	71.340 (18)
Br4—Pd1—Br2	91.64 (2)	Pd1^i^—Br3—Pd1	95.00 (2)
Br4—Pd1—Br3^i^	92.07 (2)		

Symmetry code: (i) 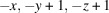.

**Table 8:** Selected geometric parameters (Å, °) for **8**[Chem scheme1]

P1—Se1	2.2505 (8)	Pd1—Br3	2.4413 (4)
Se1—Br1	2.3310 (4)	Pd2—Br4	2.4157 (4)
Br1—Br2	3.2510 (5)	Pd2—Br3	2.4562 (4)
Pd1—Br2	2.4218 (4)		
			
C2—P1—Se1	109.72 (10)	Br3—Pd1—Br3^i^	85.785 (18)
C3—P1—Se1	109.24 (10)	Br4^i^—Pd2—Br4	92.26 (2)
C1—P1—Se1	100.47 (10)	Br4—Pd2—Br3^i^	176.053 (14)
P1—Se1—Br1	100.30 (2)	Br4—Pd2—Br3	91.324 (11)
Se1—Br1—Br2	176.810 (16)	Br3^i^—Pd2—Br3	85.139 (18)
Br2—Pd1—Br2^i^	92.495 (19)	Pd1—Br2—Br1	75.133 (10)
Br2—Pd1—Br3	90.936 (11)	Pd1—Br3—Pd2	94.538 (13)
Br2—Pd1—Br3^i^	175.531 (13)		

Symmetry code: (i) 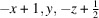.

**Table 9:** Selected geometric parameters (Å, °) for **9**[Chem scheme1]

Pd1—Se1	2.4642 (2)	Se2—P2	2.1863 (4)
Pd1—Se2	2.4662 (2)	P1—O1	1.5340 (13)
Se1—P1	2.1894 (5)	P2—O2	1.5287 (12)
			
Se1—Pd1—Se1^i^	180.0	Se2^i^—Pd1—Se2	180.0
Se1—Pd1—Se2^i^	82.184 (5)	P1—Se1—Pd1	114.028 (13)
Se1—Pd1—Se2	97.817 (5)	P2—Se2—Pd1	105.840 (13)

Symmetry code: (i) 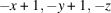.

**Table 10:** Hydrogen-bond geometry (Å, °) for **1**[Chem scheme1]

*D*—H⋯*A*	*D*—H	H⋯*A*	*D*⋯*A*	*D*—H⋯*A*
C2—H2⋯Cl1	1.00	2.85	3.501 (4)	123
C31—H31*A*⋯Cl1	0.98	2.70	3.629 (4)	158
C21—H21*B*⋯Se1	0.98	3.07	3.547 (4)	112
C11—H11*A*⋯Se1	0.98	2.93	3.419 (4)	112
C12—H12*C*⋯Se1	0.98	3.05	3.574 (4)	115

**Table 11:** Hydrogen-bond geometry (Å, °) for **2**[Chem scheme1]

*D*—H⋯*A*	*D*—H	H⋯*A*	*D*⋯*A*	*D*—H⋯*A*
C11—H11*B*⋯Se1	0.98	3.08	3.633 (3)	117
C12—H12*C*⋯Se1	0.98	2.72	3.282 (3)	117
C21—H21*C*⋯Cl1	0.98	2.77	3.741 (3)	170
C21—H21*C*⋯Se1	0.98	2.92	3.478 (3)	117
C3—H3⋯Cl2	1.00	2.77	3.608 (3)	142
C31—H31*B*⋯Se1	0.98	2.99	3.606 (3)	122
C41—H41*C*⋯Se2	0.98	3.08	3.632 (4)	117
C43—H43*A*⋯Se2	0.98	2.67	3.300 (4)	123
C5—H5⋯Cl1	1.00	2.83	3.484 (4)	124
C51—H51*C*⋯Cl1^i^	0.98	2.76	3.642 (3)	150
C52—H52*B*⋯Cl1	0.98	2.81	3.439 (4)	123
C52—H52*B*⋯Se2	0.98	2.97	3.507 (4)	116
C62—H62*B*⋯Se2	0.98	2.91	3.456 (4)	116
C62—H62*C*⋯Cl1	0.98	2.82	3.552 (4)	132
C63—H63*A*⋯Se1^ii^	0.98	3.05	3.948 (4)	153
C63—H63*A*⋯Pd1^ii^	0.98	2.87	3.793 (4)	158

Symmetry codes: (i) 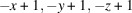; (ii) 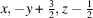.

**Table 12:** Hydrogen-bond geometry (Å, °) for **3**[Chem scheme1]

*D*—H⋯*A*	*D*—H	H⋯*A*	*D*⋯*A*	*D*—H⋯*A*
C11—H11*B*⋯Se1	0.98	3.07	3.630 (4)	117
C12—H12*C*⋯Se1	0.98	2.74	3.286 (5)	116
C21—H21*C*⋯Br1	0.98	2.88	3.843 (4)	168
C21—H21*C*⋯Se1	0.98	2.94	3.491 (4)	117
C22—H22*A*⋯Pd1	0.98	2.73	3.641 (4)	155
C3—H3⋯Br2	1.00	2.84	3.678 (4)	142
C31—H31*B*⋯Se1	0.98	3.00	3.612 (5)	122
C41—H41*C*⋯Se2	0.98	3.05	3.610 (6)	118
C43—H43*A*⋯Se2	0.98	2.70	3.307 (5)	120
C5—H5⋯Br1	1.00	2.90	3.588 (5)	127
C51—H51*C*⋯Br1^i^	0.98	2.87	3.706 (5)	144
C52—H52*B*⋯Br1	0.98	3.01	3.558 (6)	116
C52—H52*B*⋯Se2	0.98	2.96	3.545 (6)	120
C62—H62*B*⋯Se2	0.98	2.89	3.481 (6)	120
C62—H62*C*⋯Br1	0.98	3.03	3.678 (6)	124
C63—H63*A*⋯Se1^ii^	0.98	3.09	4.032 (6)	162
C63—H63*A*⋯Pd1^ii^	0.98	3.03	3.918 (6)	151

Symmetry codes: (i) 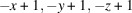; (ii) 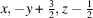.

**Table 13:** Hydrogen-bond geometry (Å, °) for **4**[Chem scheme1]

*D*—H⋯*A*	*D*—H	H⋯*A*	*D*⋯*A*	*D*—H⋯*A*
C3—H3⋯Br1	1.00	2.81	3.638 (3)	140
C11—H11*C*⋯S1	0.98	2.63	3.132 (3)	112
C21—H21*C*⋯S1	0.98	2.85	3.366 (4)	114
C32—H32*C*⋯S1	0.98	3.02	3.567 (3)	117
C22—H22*C*⋯Pd1	0.98	2.87	3.778 (4)	154
C12—H12*A*⋯S1^ii^	0.98	2.95	3.443 (3)	112
C21—H21*C*⋯Br1^i^	0.98	2.82	3.782 (4)	168

Symmetry codes: (i) 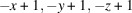; (ii) 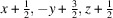.

**Table 14:** Hydrogen-bond geometry (Å, °) for **5**[Chem scheme1]

*D*—H⋯*A*	*D*—H	H⋯*A*	*D*⋯*A*	*D*—H⋯*A*
C3—H3⋯Cl1^i^	1.00	2.61	3.382 (3)	134
C5—H6⋯Cl2	1.00	2.65	3.470 (3)	140
C41—H41*A*⋯S2	0.98	3.01	3.541 (3)	115
C42—H42*C*⋯S2	0.98	2.64	3.195 (3)	116
C63—H63*B*⋯Cl2^ii^	0.98	2.85	3.666 (3)	141
C63—H63*B*⋯S2	0.98	2.85	3.374 (3)	114
C12—H12*A*⋯S1	0.98	2.67	3.188 (3)	113
C23—H23*B*⋯Cl1	0.98	2.73	3.708 (4)	175
C23—H23*B*⋯S1	0.98	2.84	3.370 (3)	115
C32—H32*B*⋯S1	0.98	2.87	3.467 (3)	121
C22—H22*C*⋯Pt1^i^	0.98	2.77	3.691 (3)	156
C51—H51*C*⋯Pt2^ii^	0.98	2.64	3.475 (3)	143
C32—H32*A*⋯Cl2^iii^	0.98	2.76	3.716 (3)	165
C43—H43*A*⋯S1^iv^	0.98	2.98	3.779 (3)	139

Symmetry codes: (i) 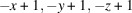; (ii) 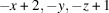; (iii) 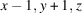; (iv) 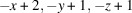.

**Table 15:** Hydrogen-bond geometry (Å, °) for **6**[Chem scheme1]

*D*—H⋯*A*	*D*—H	H⋯*A*	*D*⋯*A*	*D*—H⋯*A*
C3—H3⋯Cl1	1.00	2.74	3.466 (2)	130
C13—H13*C*⋯Cl1^ii^	0.98	2.95	3.893 (2)	162
C31—H31*B*⋯S1	0.98	2.93	3.523 (2)	120
C32—H32*C*⋯Cl1^iii^	0.98	2.89	3.728 (2)	144
C21—H21*C*⋯Cl2	0.98	2.90	3.844 (2)	161
C22—H22*C*⋯Cl2^iv^	0.98	2.99	3.960 (2)	172
C22—H22*A*⋯Pd1	0.98	2.52	3.383 (2)	147
C2—H2⋯Pd1^v^	1.00	3.09	3.688 (2)	120
C22—H22*C*⋯Pd1^v^	0.98	3.04	3.740 (2)	129
C21—H21*A*⋯Pd1^v^	0.98	3.16	3.784 (2)	123

Symmetry codes: (ii) 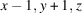; (iii) 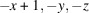; (iv) 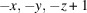; (v) 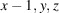.

**Table 16:** Hydrogen-bond geometry (Å, °) for **7**[Chem scheme1]

*D*—H⋯*A*	*D*—H	H⋯*A*	*D*⋯*A*	*D*—H⋯*A*
C11—H11*C*⋯Br4^ii^	0.98	3.04	4.005 (7)	167
C12—H12*C*⋯S1	0.98	3.03	3.547 (7)	114
C12—H12*C*⋯Br4^iii^	0.98	3.10	3.689 (6)	120
C13—H13*B*⋯S1	0.98	2.70	3.190 (7)	111
C2—H2⋯Br1	1.00	2.95	3.468 (5)	113
C2—H2⋯Br3^i^	1.00	3.12	3.928 (5)	139
C21—H21*B*⋯Br1	0.98	3.03	3.668 (6)	124
C21—H21*B*⋯S1	0.98	2.88	3.456 (6)	119
C21—H21*B*⋯Br3^iv^	0.98	3.10	3.968 (6)	149
C31—H31*C*⋯Br1	0.98	2.94	3.732 (11)	139
C33—H33*B*⋯S1	0.98	2.73	3.298 (10)	117

Symmetry codes: (i) 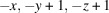; (ii) 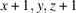; (iii) 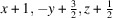; (iv) 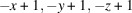.

**Table 17:** Hydrogen-bond geometry (Å, °) for **8**[Chem scheme1]

*D*—H⋯*A*	*D*—H	H⋯*A*	*D*⋯*A*	*D*—H⋯*A*
C12—H12*C*⋯Se1	0.98	3.12	3.640 (3)	115
C13—H13*B*⋯Se1	0.98	2.61	3.181 (3)	117
C21—H21*A*⋯Br3	0.98	3.14	3.822 (3)	128
C21—H21*B*⋯Se1	0.98	2.90	3.511 (3)	121
C21—H21*B*⋯Br1	0.98	2.80	3.574 (3)	137
C31—H31*B*⋯Se1	0.98	3.16	3.740 (3)	119
C32—H32*A*⋯Se1	0.98	2.97	3.526 (3)	117
C32—H32*A*⋯Br1	0.98	2.85	3.452 (3)	120
C12—H12*A*⋯Br2^ii^	0.98	2.98	3.877 (3)	152
C12—H12*C*⋯Br3^iii^	0.98	3.04	4.009 (3)	170
C2—H2⋯Br3^i^	1.00	3.03	3.928 (3)	151
C32—H32*C*⋯Br2^iv^	0.98	2.95	3.885 (3)	160

Symmetry codes: (i) 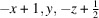; (ii) 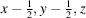; (iii) 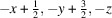; (iv) 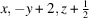.

**Table 18:** Hydrogen-bond geometry (Å, °) for **9**[Chem scheme1]

*D*—H⋯*A*	*D*—H	H⋯*A*	*D*⋯*A*	*D*—H⋯*A*
O1—H01⋯O2	0.78 (2)	1.63 (2)	2.4156 (17)	178 (4)
O2—H02⋯O1	0.78 (2)	1.64 (2)	2.4156 (17)	172 (5)
C11—H11*B*⋯Se1	0.98	2.95	3.4748 (18)	115
C21—H21*A*⋯O2^ii^	0.98	2.52	3.407 (2)	150
C32—H32*C*⋯Se2^iii^	0.98	3.13	3.8999 (17)	136
C41—H41*B*⋯Se2	0.98	2.94	3.4825 (18)	116
C42—H42*A*⋯Se1^iv^	0.98	2.98	3.8370 (19)	146
C32—H32*A*⋯Pd1	0.98	2.69	3.4897 (18)	139

Symmetry codes: (ii) 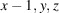; (iii) 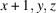; (iv) 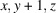.

**Table:** 

	**1**	**2**	**3**	**4**	**5**
Crystal data					
Chemical formula	[PdCl_2_(C_10_H_23_PSe)_2_]	[PdCl_2_(C_11_H_25_PSe)_2_]	[PdBr_2_(C_11_H_25_PSe)_2_]	[PdBr_2_(C_11_H_25_PS)_2_]	[PdCl_2_(C_11_H_25_PS)_2_]
*M*_r_	683.73	711.78	800.70	706.90	706.67
Crystal system, space group	Triclinic, *P* 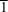	Monoclinic, *P*2_1_/*c*	Monoclinic, *P*2_1_/*c*	Monoclinic, *P*2_1_/*n*	Monoclinic, *P*2_1_/*c*
Temperature (K)	100	100	100	100	100
*a*, *b*, *c* (Å)	7.9312 (5), 8.5664 (6), 10.1483 (7)	15.3744 (4), 13.2969 (2), 16.0503 (3)	15.3561 (6), 13.4695 (4), 16.1371 (6)	7.8595 (3), 17.5019 (6), 10.6740 (3)	14.5283 (3), 14.4191 (3), 13.9428 (4)
α, β, γ (°)	88.993 (6), 88.010 (6), 77.063 (6)	90, 117.306 (3), 90	90, 116.558 (5), 90	90, 94.551 (3), 90	90, 94.571 (3), 90
*V* (Å^3^)	671.55 (8)	2915.56 (12)	2985.6 (2)	1463.65 (9)	2911.53 (12)
*Z*	1	4	4	2	4
Radiation type	Mo *K*α	Mo *K*α	Mo *K*α	Mo *K*α	Mo *K*α
μ (mm^−1^)	3.73	3.44	5.85	3.63	5.27
Crystal size (mm)	0.2 × 0.1 × 0.01	0.20 × 0.10 × 0.02	0.18 × 0.05 × 0.02	0.2 × 0.08 × 0.05	0.2 × 0.1 × 0.07

Data collection					
Diffractometer	Oxford Diffraction Xcalibur, Eos	Oxford Diffraction Xcalibur, Eos	Oxford Diffraction Xcalibur, Eos	Oxford Diffraction Xcalibur, Eos	Oxford Diffraction Xcalibur, Eos
Absorption correction	Multi-scan (*CrysAlis PRO*; Rigaku OD, 2012 ▸)	Multi-scan (*CrysAlis PRO*; Rigaku OD, 2012 ▸)	Multi-scan (*CrysAlis PRO*; Rigaku OD, 2012 ▸)	Multi-scan (*CrysAlis PRO*; Rigaku OD, 2012 ▸)	Multi-scan (*CrysAlis PRO*; Rigaku OD, 2012 ▸)
*T*_min_, *T*_max_	0.841, 1.000	0.713, 1.000	0.419, 0.892	0.865, 1.000	0.650, 1.000
No. of measured, independent and observed [*I* > 2σ(*I*)] reflections	20956, 3275, 2604	134870, 7231, 5841	120046, 7402, 5854	45160, 4422, 3832	79369, 8721, 6105
*R*_int_	0.072	0.091	0.104	0.050	0.044
θ values (°)	θ_max_ = 28.3, θ_min_ = 2.4	θ_max_ = 28.3, θ_min_ = 2.1	θ_max_ = 28.3, θ_min_ = 2.1	θ_max_ = 30.9, θ_min_ = 2.2	θ_max_ = 30.9, θ_min_ = 2.4
(sin θ/λ)_max_ (Å^−1^)	0.667	0.667	0.667	0.721	0.722

Refinement					
*R*[*F*^2^ > 2σ(*F*^2^)], *wR*(*F*^2^), *S*	0.036, 0.078, 1.04	0.032, 0.068, 1.04	0.039, 0.083, 1.04	0.039, 0.065, 1.22	0.026, 0.055, 1.04
No. of reflections	3275	7231	7402	4422	8721
No. of parameters	131	278	278	141	281
No. of restraints	0	0	0	0	0
H-atom treatment	H-atom parameters constrained	H-atom parameters constrained	H-atom parameters constrained	H-atom parameters constrained	H-atom parameters constrained
Δρ_max_, Δρ_min_ (e Å^−3^)	0.93, −0.98	1.35, −0.78	2.68, −1.32	1.02, −0.70	1.14, −0.97

**Table:** 

	**6**	**7**)	**8**	**9**
Crystal data				
Chemical formula	[PdCl_2_(C_10_H_23_PS)_2_]	(C_11_H_25_BrPS)_2_[Pd_2_Br_6_]	(C_11_H_25_BrPS_2_)_2_[Pd_2_Br_6_]	[Pd(C_6_H_14_OP)_2_(C_6_H_15_OP)_2_]
*M*_r_	767.23	1292.76	1386.56	956.82
Crystal system, space group	Triclinic, *P* 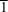	Monoclinic, *P*2_1_/*c*	Monoclinic, *C*2/*c*	Monoclinic, *P*2_1_/*n*
Temperature (K)	100	100	100	100
*a*, *b*, *c* (Å)	6.9753 (4), 8.7642 (5), 13.0718 (7)	7.8691 (4), 22.7255 (8), 10.5879 (3)	19.3550 (6), 14.8165 (2), 16.3047 (5)	7.56435 (6), 10.09140 (9), 24.13960 (19)
α, β, γ (°)	88.930 (6), 78.488 (6), 79.804 (7)	90, 98.386 (3), 90	90, 125.957 (5), 90	90, 92.7641 (8), 90
*V* (Å^3^)	770.56 (8)	1873.18 (13)	3784.8 (3)	1840.55 (3)
*Z*	1	2	4	2
Radiation type	Mo *K*α	Mo *K*α	Mo *K*α	Mo *K*α
μ (mm^−1^)	1.76	9.70	11.42	4.66
Crystal size (mm)	0.17 × 0.06 × 0.02	0.2 × 0.1 × 0.01	0.2 × 0.06 × 0.02	0.10 × 0.08 × 0.04

Data collection				
Diffractometer	Oxford Diffraction Xcalibur, Eos	Oxford Diffraction Xcalibur, Eos	Oxford Diffraction Xcalibur, Eos	Oxford Diffraction Xcalibur, Eos
Absorption correction	Multi-scan (*CrysAlis PRO*; Rigaku OD, 2012 ▸)	Multi-scan (*CrysAlis PRO*; Rigaku OD, 2012 ▸)	Multi-scan (*CrysAlis PRO*; Rigaku OD, 2012 ▸)	Multi-scan (*CrysAlis PRO*; Rigaku OD, 2012 ▸)
*T*_min_, *T*_max_	0.754, 0.966	0.495, 1.000	0.327, 1.000	0.650, 1.000
No. of measured, independent and observed [*I* > 2σ(*I*)] reflections	48215, 4550, 3905	52903, 4622, 3740	51432, 5622, 4433	86102, 5558, 4939
*R*_int_	0.052	0.082	0.074	0.047
θ values (°)	θ_max_ = 30.8, θ_min_ = 2.4	θ_max_ = 28.3, θ_min_ = 2.1	θ_max_ = 30.9, θ_min_ = 2.6	θ_max_ = 30.9, θ_min_ = 2.2
(sin θ/λ)_max_ (Å^−1^)	0.721	0.667	0.722	0.722

Refinement				
*R*[*F*^2^ > 2σ(*F*^2^)], *wR*(*F*^2^), *S*	0.029, 0.059, 1.05	0.042, 0.072, 1.11	0.031, 0.057, 1.04	0.022, 0.041, 1.07
No. of reflections	4550	4622	5622	5558
No. of parameters	143	196	172	185
No. of restraints	0	87	0	2
H-atom treatment	H-atom parameters constrained	H-atom parameters constrained	H-atom parameters constrained	H atoms treated by a mixture of independent and constrained refinement
Δρ_max_, Δρ_min_ (e Å^−3^)	1.29, −0.58	0.71, −0.95	0.97, −1.02	0.47, −0.54

Computer programs: *CrysAlis PRO* (Rigaku OD, 2012 ▸), *SHELXS97* (Sheldrick, 2008 ▸), *SHELXL2019/3* (Sheldrick, 2015 ▸), *XP* (Bruker, 1998 ▸) and *publCIF* (Westrip, 2010 ▸).

## supplementary crystallographic information

### (tert-Butyldiisopropylphosphine selenide-κSe)dichloridopalladium(II) (1) Crystal data

**Table:**

[PdCl_2_(C_10_H_23_PSe)_2_]	*Z* = 1
*M**_r_* = 683.73	*F*(000) = 344
Triclinic, *P*1	*D*_x_ = 1.691 Mg m^−^^3^
*a* = 7.9312 (5) Å	Mo *K*α radiation, λ = 0.71073 Å
*b* = 8.5664 (6) Å	Cell parameters from 4517 reflections
*c* = 10.1483 (7) Å	θ = 2.4–29.3°
α = 88.993 (6)°	µ = 3.73 mm^−^^1^
β = 88.010 (6)°	*T* = 100 K
γ = 77.063 (6)°	Thin plate, dichroic yellow orange
*V* = 671.55 (8) Å^3^	0.2 × 0.1 × 0.01 mm

### (tert-Butyldiisopropylphosphine selenide-κSe)dichloridopalladium(II) (1) Data collection

**Table:**

Oxford Diffraction Xcalibur, Eos diffractometer	3275 independent reflections
Radiation source: fine-focus sealed tube	2604 reflections with *I* > 2σ(*I*)
Graphite monochromator	*R*_int_ = 0.072
Detector resolution: 16.1419 pixels mm^-1^	θ_max_ = 28.3°, θ_min_ = 2.4°
ω scans	*h* = −10→10
Absorption correction: multi-scan (CrysAlisPro; Rigaku OD, 2012)	*k* = −11→10
*T*_min_ = 0.841, *T*_max_ = 1.000	*l* = −13→13
20956 measured reflections	

### (tert-Butyldiisopropylphosphine selenide-κSe)dichloridopalladium(II) (1) Refinement

**Table:**

Refinement on *F*^2^	Primary atom site location: structure-invariant direct methods
Least-squares matrix: full	Secondary atom site location: difference Fourier map
*R*[*F*^2^ > 2σ(*F*^2^)] = 0.036	Hydrogen site location: inferred from neighbouring sites
*wR*(*F*^2^) = 0.078	H-atom parameters constrained
*S* = 1.04	*w* = 1/[σ^2^(*F*_o_^2^) + (0.0307*P*)^2^ + 0.4792*P*] where *P* = (*F*_o_^2^ + 2*F*_c_^2^)/3
3275 reflections	(Δ/σ)_max_ < 0.001
131 parameters	Δρ_max_ = 0.93 e Å^−^^3^
0 restraints	Δρ_min_ = −0.98 e Å^−^^3^

### (tert-Butyldiisopropylphosphine selenide-κSe)dichloridopalladium(II) (1) Fractional atomic coordinates and isotropic or equivalent isotropic displacement parameters (Å^2^)

**Table:**

	*x*	*y*	*z*	*U*_iso_*/*U*_eq_	
Pd1	0.000000	0.500000	0.000000	0.01285 (10)	
Se1	0.07012 (4)	0.44262 (4)	0.22978 (4)	0.01727 (11)	
P1	0.29069 (11)	0.24483 (11)	0.26911 (9)	0.01228 (19)	
Cl1	0.17759 (11)	0.27541 (10)	−0.09307 (9)	0.0190 (2)	
C1	0.2805 (4)	0.2272 (4)	0.4510 (3)	0.0147 (7)	
H1	0.385348	0.145781	0.477693	0.018*	
C11	0.2856 (5)	0.3841 (4)	0.5188 (4)	0.0203 (8)	
H11A	0.180031	0.464600	0.500005	0.030*	
H11B	0.386789	0.422371	0.485510	0.030*	
H11C	0.293216	0.365997	0.614217	0.030*	
C12	0.1217 (5)	0.1681 (5)	0.5024 (4)	0.0201 (8)	
H12A	0.120148	0.164458	0.598972	0.030*	
H12B	0.127039	0.060539	0.468581	0.030*	
H12C	0.016410	0.241323	0.472417	0.030*	
C2	0.2789 (4)	0.0519 (4)	0.1971 (4)	0.0165 (7)	
H2	0.327920	0.052510	0.104964	0.020*	
C21	0.0930 (5)	0.0285 (5)	0.1851 (4)	0.0223 (8)	
H21A	0.096198	−0.072961	0.140989	0.034*	
H21B	0.025579	0.117118	0.133495	0.034*	
H21C	0.039096	0.026597	0.273322	0.034*	
C22	0.3941 (5)	−0.0906 (4)	0.2679 (4)	0.0230 (9)	
H22A	0.351376	−0.096425	0.359266	0.034*	
H22B	0.513302	−0.076338	0.267243	0.034*	
H22C	0.391017	−0.189887	0.222544	0.034*	
C3	0.5088 (4)	0.2865 (4)	0.2197 (3)	0.0158 (7)	
C31	0.5749 (5)	0.2080 (5)	0.0875 (4)	0.0213 (8)	
H31A	0.486334	0.241179	0.021383	0.032*	
H31B	0.599828	0.091189	0.097984	0.032*	
H31C	0.680742	0.241374	0.058531	0.032*	
C32	0.6447 (4)	0.2221 (5)	0.3233 (4)	0.0221 (8)	
H32A	0.756830	0.242050	0.293247	0.033*	
H32B	0.654933	0.106606	0.335441	0.033*	
H32C	0.609030	0.276447	0.407183	0.033*	
C33	0.4890 (5)	0.4678 (4)	0.2038 (4)	0.0229 (8)	
H33A	0.442647	0.520661	0.286371	0.034*	
H33B	0.409494	0.507633	0.132710	0.034*	
H33C	0.602237	0.491191	0.181993	0.034*	

### (tert-Butyldiisopropylphosphine selenide-κSe)dichloridopalladium(II) (1) Atomic displacement parameters (Å^2^)

**Table:**

	*U*^11^	*U*^22^	*U*^33^	*U*^12^	*U*^13^	*U*^23^
Pd1	0.00951 (19)	0.0118 (2)	0.0158 (2)	0.00070 (14)	−0.00103 (14)	0.00080 (15)
Se1	0.01435 (19)	0.0168 (2)	0.0165 (2)	0.00561 (14)	−0.00110 (14)	−0.00058 (15)
P1	0.0096 (4)	0.0118 (4)	0.0143 (4)	0.0003 (3)	−0.0011 (3)	0.0002 (3)
Cl1	0.0177 (4)	0.0153 (4)	0.0202 (5)	0.0042 (3)	−0.0011 (3)	−0.0009 (3)
C1	0.0154 (17)	0.0149 (18)	0.0130 (17)	−0.0015 (14)	0.0004 (14)	−0.0022 (14)
C11	0.0192 (19)	0.023 (2)	0.0169 (19)	−0.0019 (16)	0.0009 (15)	−0.0029 (15)
C12	0.0211 (19)	0.022 (2)	0.0168 (19)	−0.0048 (16)	0.0016 (15)	0.0004 (15)
C2	0.0150 (17)	0.0149 (18)	0.0175 (19)	0.0008 (14)	0.0014 (14)	−0.0003 (14)
C21	0.022 (2)	0.024 (2)	0.023 (2)	−0.0087 (16)	−0.0015 (16)	−0.0014 (16)
C22	0.026 (2)	0.0118 (18)	0.029 (2)	−0.0001 (15)	−0.0001 (17)	−0.0015 (16)
C3	0.0110 (17)	0.0223 (19)	0.0141 (18)	−0.0045 (14)	0.0016 (13)	−0.0001 (14)
C31	0.0179 (19)	0.027 (2)	0.019 (2)	−0.0064 (16)	0.0032 (15)	−0.0019 (16)
C32	0.0117 (17)	0.030 (2)	0.025 (2)	−0.0054 (16)	−0.0002 (15)	0.0011 (17)
C33	0.024 (2)	0.020 (2)	0.026 (2)	−0.0088 (16)	0.0010 (16)	0.0018 (16)

### (tert-Butyldiisopropylphosphine selenide-κSe)dichloridopalladium(II) (1) Geometric parameters (Å, º)

**Table:**

Pd1—Cl1^i^	2.3099 (8)	C2—H2	1.0000
Pd1—Cl1	2.3099 (8)	C21—H21A	0.9800
Pd1—Se1^i^	2.4322 (4)	C21—H21B	0.9800
Pd1—Se1	2.4322 (4)	C21—H21C	0.9800
Se1—P1	2.1881 (9)	C22—H22A	0.9800
P1—C2	1.842 (4)	C22—H22B	0.9800
P1—C1	1.850 (3)	C22—H22C	0.9800
P1—C3	1.892 (3)	C3—C33	1.532 (5)
C1—C11	1.530 (5)	C3—C31	1.534 (5)
C1—C12	1.531 (5)	C3—C32	1.538 (5)
C1—H1	1.0000	C31—H31A	0.9800
C11—H11A	0.9800	C31—H31B	0.9800
C11—H11B	0.9800	C31—H31C	0.9800
C11—H11C	0.9800	C32—H32A	0.9800
C12—H12A	0.9800	C32—H32B	0.9800
C12—H12B	0.9800	C32—H32C	0.9800
C12—H12C	0.9800	C33—H33A	0.9800
C2—C22	1.535 (5)	C33—H33B	0.9800
C2—C21	1.541 (5)	C33—H33C	0.9800
			
Cl1^i^—Pd1—Cl1	180.0	C2—C21—H21A	109.5
Cl1^i^—Pd1—Se1^i^	98.63 (2)	C2—C21—H21B	109.5
Cl1—Pd1—Se1^i^	81.37 (2)	H21A—C21—H21B	109.5
Cl1^i^—Pd1—Se1	81.37 (2)	C2—C21—H21C	109.5
Cl1—Pd1—Se1	98.63 (2)	H21A—C21—H21C	109.5
Se1^i^—Pd1—Se1	180.0	H21B—C21—H21C	109.5
P1—Se1—Pd1	116.92 (3)	C2—C22—H22A	109.5
C2—P1—C1	109.10 (16)	C2—C22—H22B	109.5
C2—P1—C3	107.77 (16)	H22A—C22—H22B	109.5
C1—P1—C3	107.62 (16)	C2—C22—H22C	109.5
C2—P1—Se1	114.92 (11)	H22A—C22—H22C	109.5
C1—P1—Se1	102.53 (11)	H22B—C22—H22C	109.5
C3—P1—Se1	114.48 (12)	C33—C3—C31	108.2 (3)
C11—C1—C12	109.5 (3)	C33—C3—C32	109.4 (3)
C11—C1—P1	112.4 (2)	C31—C3—C32	108.1 (3)
C12—C1—P1	112.7 (2)	C33—C3—P1	108.7 (2)
C11—C1—H1	107.3	C31—C3—P1	110.8 (2)
C12—C1—H1	107.3	C32—C3—P1	111.5 (2)
P1—C1—H1	107.3	C3—C31—H31A	109.5
C1—C11—H11A	109.5	C3—C31—H31B	109.5
C1—C11—H11B	109.5	H31A—C31—H31B	109.5
H11A—C11—H11B	109.5	C3—C31—H31C	109.5
C1—C11—H11C	109.5	H31A—C31—H31C	109.5
H11A—C11—H11C	109.5	H31B—C31—H31C	109.5
H11B—C11—H11C	109.5	C3—C32—H32A	109.5
C1—C12—H12A	109.5	C3—C32—H32B	109.5
C1—C12—H12B	109.5	H32A—C32—H32B	109.5
H12A—C12—H12B	109.5	C3—C32—H32C	109.5
C1—C12—H12C	109.5	H32A—C32—H32C	109.5
H12A—C12—H12C	109.5	H32B—C32—H32C	109.5
H12B—C12—H12C	109.5	C3—C33—H33A	109.5
C22—C2—C21	111.5 (3)	C3—C33—H33B	109.5
C22—C2—P1	112.1 (3)	H33A—C33—H33B	109.5
C21—C2—P1	113.9 (2)	C3—C33—H33C	109.5
C22—C2—H2	106.2	H33A—C33—H33C	109.5
C21—C2—H2	106.2	H33B—C33—H33C	109.5
P1—C2—H2	106.2		
			
Cl1^i^—Pd1—Se1—P1	−169.36 (4)	Se1—P1—C2—C22	−158.3 (2)
Cl1—Pd1—Se1—P1	10.64 (4)	C1—P1—C2—C21	84.0 (3)
Pd1—Se1—P1—C2	−55.00 (14)	C3—P1—C2—C21	−159.4 (3)
Pd1—Se1—P1—C1	−173.22 (11)	Se1—P1—C2—C21	−30.5 (3)
Pd1—Se1—P1—C3	70.55 (13)	C2—P1—C3—C33	147.4 (3)
C2—P1—C1—C11	−178.8 (2)	C1—P1—C3—C33	−95.1 (3)
C3—P1—C1—C11	64.6 (3)	Se1—P1—C3—C33	18.2 (3)
Se1—P1—C1—C11	−56.5 (3)	C2—P1—C3—C31	28.6 (3)
C2—P1—C1—C12	−54.5 (3)	C1—P1—C3—C31	146.1 (3)
C3—P1—C1—C12	−171.2 (2)	Se1—P1—C3—C31	−100.6 (2)
Se1—P1—C1—C12	67.8 (2)	C2—P1—C3—C32	−91.9 (3)
C1—P1—C2—C22	−43.8 (3)	C1—P1—C3—C32	25.7 (3)
C3—P1—C2—C22	72.8 (3)	Se1—P1—C3—C32	138.9 (2)

Symmetry code: (i) −*x*, −*y*+1, −*z*.

### (tert-Butyldiisopropylphosphine selenide-κSe)dichloridopalladium(II) (1) Hydrogen-bond geometry (Å, º)

**Table:**

*D*—H···*A*	*D*—H	H···*A*	*D*···*A*	*D*—H···*A*
C2—H2···Cl1	1.00	2.85	3.501 (4)	123
C31—H31*A*···Cl1	0.98	2.70	3.629 (4)	158
C21—H21*B*···Se1	0.98	3.07	3.547 (4)	112
C11—H11*A*···Se1	0.98	2.93	3.419 (4)	112
C12—H12*C*···Se1	0.98	3.05	3.574 (4)	115

### (Di-tert-butylisopropylphosphine selenide-κSe)dichloridopalladium(II) (2) Crystal data

**Table:**

[PdCl_2_(C_11_H_25_PSe)_2_]	*F*(000) = 1440
*M**_r_* = 711.78	*D*_x_ = 1.622 Mg m^−^^3^
Monoclinic, *P*2_1_/*c*	Mo *K*α radiation, λ = 0.71073 Å
*a* = 15.3744 (4) Å	Cell parameters from 15039 reflections
*b* = 13.2969 (2) Å	θ = 2.1–30.8°
*c* = 16.0503 (3) Å	µ = 3.44 mm^−^^1^
β = 117.306 (3)°	*T* = 100 K
*V* = 2915.56 (12) Å^3^	Plate, dichroic red orange
*Z* = 4	0.20 × 0.10 × 0.02 mm

### (Di-tert-butylisopropylphosphine selenide-κSe)dichloridopalladium(II) (2) Data collection

**Table:**

Oxford Diffraction Xcalibur, Eos diffractometer	7231 independent reflections
Radiation source: Enhance (Mo) X-ray Source	5841 reflections with *I* > 2σ(*I*)
Graphite monochromator	*R*_int_ = 0.091
Detector resolution: 16.1419 pixels mm^-1^	θ_max_ = 28.3°, θ_min_ = 2.1°
ω scans	*h* = −20→20
Absorption correction: multi-scan (CrysAlisPro; Rigaku OD, 2012)	*k* = −17→17
*T*_min_ = 0.713, *T*_max_ = 1.000	*l* = −21→21
134870 measured reflections	

### (Di-tert-butylisopropylphosphine selenide-κSe)dichloridopalladium(II) (2) Refinement

**Table:**

Refinement on *F*^2^	Primary atom site location: structure-invariant direct methods
Least-squares matrix: full	Secondary atom site location: difference Fourier map
*R*[*F*^2^ > 2σ(*F*^2^)] = 0.032	Hydrogen site location: inferred from neighbouring sites
*wR*(*F*^2^) = 0.068	H-atom parameters constrained
*S* = 1.04	*w* = 1/[σ^2^(*F*_o_^2^) + (0.0234*P*)^2^ + 4.2293*P*] where *P* = (*F*_o_^2^ + 2*F*_c_^2^)/3
7231 reflections	(Δ/σ)_max_ = 0.001
278 parameters	Δρ_max_ = 1.35 e Å^−^^3^
0 restraints	Δρ_min_ = −0.78 e Å^−^^3^

### (Di-tert-butylisopropylphosphine selenide-κSe)dichloridopalladium(II) (2) Special details

**Table:**

Geometry. All esds (except the esd in the dihedral angle between two l.s. planes) are estimated using the full covariance matrix. The cell esds are taken into account individually in the estimation of esds in distances, angles and torsion angles; correlations between esds in cell parameters are only used when they are defined by crystal symmetry. An approximate (isotropic) treatment of cell esds is used for estimating esds involving l.s. planes.

### (Di-tert-butylisopropylphosphine selenide-κSe)dichloridopalladium(II) (2) Fractional atomic coordinates and isotropic or equivalent isotropic displacement parameters (Å^2^)

**Table:**

	*x*	*y*	*z*	*U*_iso_*/*U*_eq_	
Pd1	0.21631 (2)	0.75350 (2)	0.43474 (2)	0.01454 (6)	
Cl1	0.27119 (6)	0.59216 (5)	0.43300 (6)	0.02429 (16)	
Cl2	0.16916 (5)	0.91456 (5)	0.45175 (5)	0.02071 (15)	
Se1	0.08372 (2)	0.67680 (2)	0.45622 (2)	0.01735 (7)	
Se2	0.34701 (2)	0.84936 (2)	0.42322 (2)	0.02034 (7)	
P1	−0.05382 (5)	0.71107 (5)	0.33209 (5)	0.01336 (14)	
P2	0.43631 (6)	0.77169 (6)	0.36886 (5)	0.02029 (17)	
C1	−0.1433 (2)	0.6199 (2)	0.3408 (2)	0.0179 (6)	
C11	−0.1219 (2)	0.5134 (2)	0.3182 (2)	0.0234 (7)	
H11A	−0.160709	0.464688	0.332998	0.035*	
H11B	−0.052156	0.498536	0.355833	0.035*	
H11C	−0.139325	0.508954	0.251481	0.035*	
C12	−0.1301 (2)	0.6188 (2)	0.4422 (2)	0.0257 (7)	
H12A	−0.176767	0.571550	0.446459	0.039*	
H12B	−0.141840	0.686386	0.459183	0.039*	
H12C	−0.063198	0.597785	0.485175	0.039*	
C13	−0.2503 (2)	0.6480 (2)	0.2754 (2)	0.0259 (7)	
H13A	−0.260177	0.651640	0.210622	0.039*	
H13B	−0.265127	0.713400	0.293923	0.039*	
H13C	−0.293788	0.596718	0.279834	0.039*	
C2	−0.0416 (2)	0.6943 (2)	0.2212 (2)	0.0176 (6)	
C21	0.0215 (2)	0.6025 (2)	0.2280 (2)	0.0228 (7)	
H21A	0.031078	0.598683	0.171795	0.034*	
H21B	−0.011447	0.541326	0.232944	0.034*	
H21C	0.085160	0.608671	0.283618	0.034*	
C22	0.0092 (2)	0.7880 (2)	0.2074 (2)	0.0205 (6)	
H22A	0.070588	0.800034	0.264343	0.031*	
H22B	−0.033787	0.846468	0.194614	0.031*	
H22C	0.023270	0.776957	0.154365	0.031*	
C23	−0.1426 (2)	0.6809 (2)	0.1351 (2)	0.0222 (6)	
H23A	−0.134537	0.679690	0.077960	0.033*	
H23B	−0.185338	0.736972	0.131925	0.033*	
H23C	−0.171914	0.617463	0.140730	0.033*	
C3	−0.0861 (2)	0.8448 (2)	0.3365 (2)	0.0173 (6)	
H3	−0.029117	0.883940	0.339530	0.021*	
C31	−0.0912 (3)	0.8716 (2)	0.4271 (2)	0.0264 (7)	
H31A	−0.088046	0.944789	0.435029	0.040*	
H31B	−0.035950	0.840529	0.480612	0.040*	
H31C	−0.152747	0.846544	0.423459	0.040*	
C32	−0.1759 (2)	0.8883 (2)	0.2515 (2)	0.0241 (7)	
H32A	−0.235194	0.854274	0.244931	0.036*	
H32B	−0.168594	0.877950	0.194552	0.036*	
H32C	−0.180919	0.960508	0.260924	0.036*	
C4	0.5372 (3)	0.8675 (3)	0.3899 (3)	0.0310 (8)	
C41	0.6073 (3)	0.8667 (3)	0.4940 (3)	0.0420 (10)	
H41A	0.655858	0.920298	0.508305	0.063*	
H41B	0.640662	0.801528	0.511255	0.063*	
H41C	0.570742	0.877751	0.529645	0.063*	
C42	0.5944 (3)	0.8430 (3)	0.3349 (3)	0.0440 (10)	
H42A	0.645149	0.894025	0.348349	0.066*	
H42B	0.549428	0.842777	0.267542	0.066*	
H42C	0.625043	0.776703	0.353712	0.066*	
C43	0.4951 (3)	0.9741 (3)	0.3629 (3)	0.0359 (8)	
H43A	0.453950	0.989210	0.393325	0.054*	
H43B	0.455536	0.978337	0.294732	0.054*	
H43C	0.548795	1.022783	0.383513	0.054*	
C5	0.4908 (3)	0.6542 (3)	0.4317 (2)	0.0287 (7)	
H5	0.438287	0.602317	0.402417	0.034*	
C51	0.5791 (3)	0.6127 (3)	0.4210 (3)	0.0400 (9)	
H51A	0.636018	0.656369	0.454863	0.060*	
H51B	0.563010	0.611008	0.354459	0.060*	
H51C	0.594383	0.544578	0.446984	0.060*	
C52	0.5152 (3)	0.6556 (3)	0.5356 (2)	0.0375 (9)	
H52A	0.535191	0.588088	0.562099	0.056*	
H52B	0.457333	0.676426	0.541816	0.056*	
H52C	0.568761	0.703035	0.569321	0.056*	
C6	0.3601 (3)	0.7436 (3)	0.2415 (2)	0.0294 (7)	
C61	0.4087 (3)	0.6709 (3)	0.2026 (3)	0.0425 (10)	
H61A	0.419072	0.606082	0.234969	0.064*	
H61B	0.471833	0.698348	0.212523	0.064*	
H61C	0.366455	0.661231	0.135372	0.064*	
C62	0.2628 (3)	0.6983 (3)	0.2264 (3)	0.0362 (9)	
H62A	0.222619	0.683661	0.159586	0.054*	
H62B	0.228373	0.746102	0.247212	0.054*	
H62C	0.275005	0.635872	0.262518	0.054*	
C63	0.3368 (3)	0.8428 (3)	0.1851 (2)	0.0372 (9)	
H63A	0.289663	0.829621	0.119645	0.056*	
H63B	0.397166	0.870319	0.187677	0.056*	
H63C	0.308914	0.891380	0.212100	0.056*	

### (Di-tert-butylisopropylphosphine selenide-κSe)dichloridopalladium(II) (2) Atomic displacement parameters (Å^2^)

**Table:**

	*U*^11^	*U*^22^	*U*^33^	*U*^12^	*U*^13^	*U*^23^
Pd1	0.01474 (11)	0.01251 (10)	0.01476 (11)	0.00092 (8)	0.00538 (9)	0.00004 (8)
Cl1	0.0229 (4)	0.0153 (3)	0.0320 (4)	0.0039 (3)	0.0103 (3)	0.0004 (3)
Cl2	0.0209 (4)	0.0151 (3)	0.0267 (4)	0.0009 (3)	0.0115 (3)	−0.0021 (3)
Se1	0.01708 (15)	0.01778 (14)	0.01473 (14)	−0.00054 (11)	0.00516 (12)	0.00452 (11)
Se2	0.02171 (16)	0.01730 (15)	0.02555 (17)	−0.00316 (12)	0.01387 (14)	−0.00411 (12)
P1	0.0149 (4)	0.0115 (3)	0.0129 (3)	−0.0003 (3)	0.0056 (3)	0.0005 (3)
P2	0.0180 (4)	0.0242 (4)	0.0187 (4)	0.0048 (3)	0.0084 (3)	0.0027 (3)
C1	0.0193 (15)	0.0139 (14)	0.0196 (15)	−0.0036 (11)	0.0081 (13)	−0.0002 (11)
C11	0.0243 (17)	0.0143 (14)	0.0305 (18)	−0.0033 (12)	0.0118 (15)	0.0015 (12)
C12	0.0312 (18)	0.0243 (16)	0.0279 (17)	−0.0056 (13)	0.0190 (16)	0.0021 (13)
C13	0.0201 (16)	0.0254 (16)	0.0333 (18)	−0.0030 (13)	0.0133 (15)	0.0013 (14)
C2	0.0189 (15)	0.0191 (15)	0.0143 (14)	0.0008 (11)	0.0071 (12)	0.0013 (11)
C21	0.0225 (16)	0.0265 (17)	0.0196 (16)	0.0024 (13)	0.0099 (14)	−0.0026 (13)
C22	0.0240 (16)	0.0221 (15)	0.0169 (15)	−0.0010 (12)	0.0106 (13)	0.0020 (12)
C23	0.0211 (16)	0.0264 (16)	0.0169 (15)	0.0001 (13)	0.0068 (13)	0.0001 (12)
C3	0.0200 (15)	0.0127 (13)	0.0203 (15)	0.0007 (11)	0.0101 (13)	0.0007 (11)
C31	0.0347 (19)	0.0201 (16)	0.0276 (17)	0.0025 (14)	0.0171 (16)	−0.0037 (13)
C32	0.0247 (17)	0.0186 (15)	0.0280 (17)	0.0033 (13)	0.0112 (15)	0.0034 (13)
C4	0.0270 (18)	0.0299 (18)	0.039 (2)	0.0024 (14)	0.0177 (17)	0.0086 (15)
C41	0.026 (2)	0.040 (2)	0.048 (2)	−0.0067 (16)	0.0070 (18)	0.0027 (18)
C42	0.038 (2)	0.044 (2)	0.062 (3)	0.0066 (18)	0.033 (2)	0.011 (2)
C43	0.036 (2)	0.0285 (19)	0.046 (2)	−0.0031 (15)	0.0219 (19)	0.0064 (16)
C5	0.0279 (18)	0.0256 (17)	0.0322 (19)	0.0050 (14)	0.0134 (16)	0.0062 (14)
C51	0.035 (2)	0.034 (2)	0.051 (2)	0.0194 (17)	0.0195 (19)	0.0160 (18)
C52	0.036 (2)	0.041 (2)	0.031 (2)	0.0045 (17)	0.0115 (17)	0.0150 (16)
C6	0.0308 (18)	0.0340 (19)	0.0226 (17)	0.0049 (15)	0.0116 (15)	−0.0014 (14)
C61	0.046 (2)	0.049 (2)	0.034 (2)	0.0139 (19)	0.0201 (19)	−0.0064 (18)
C62	0.030 (2)	0.043 (2)	0.0292 (19)	−0.0094 (17)	0.0088 (16)	−0.0113 (16)
C63	0.044 (2)	0.044 (2)	0.0217 (18)	0.0098 (18)	0.0131 (17)	0.0049 (16)

### (Di-tert-butylisopropylphosphine selenide-κSe)dichloridopalladium(II) (2) Geometric parameters (Å, º)

**Table:**

Pd1—Cl1	2.3100 (7)	C31—H31A	0.9800
Pd1—Cl2	2.3169 (7)	C31—H31B	0.9800
Pd1—Se1	2.4411 (4)	C31—H31C	0.9800
Pd1—Se2	2.4573 (4)	C32—H32A	0.9800
Se1—P1	2.1870 (8)	C32—H32B	0.9800
Se2—P2	2.1936 (8)	C32—H32C	0.9800
P1—C3	1.856 (3)	C4—C41	1.517 (5)
P1—C2	1.886 (3)	C4—C43	1.535 (5)
P1—C1	1.887 (3)	C4—C42	1.541 (5)
P2—C5	1.840 (3)	C41—H41A	0.9800
P2—C6	1.869 (3)	C41—H41B	0.9800
P2—C4	1.913 (4)	C41—H41C	0.9800
C1—C11	1.534 (4)	C42—H42A	0.9800
C1—C13	1.537 (4)	C42—H42B	0.9800
C1—C12	1.544 (4)	C42—H42C	0.9800
C11—H11A	0.9800	C43—H43A	0.9800
C11—H11B	0.9800	C43—H43B	0.9800
C11—H11C	0.9800	C43—H43C	0.9800
C12—H12A	0.9800	C5—C52	1.532 (5)
C12—H12B	0.9800	C5—C51	1.547 (5)
C12—H12C	0.9800	C5—H5	1.0000
C13—H13A	0.9800	C51—H51A	0.9800
C13—H13B	0.9800	C51—H51B	0.9800
C13—H13C	0.9800	C51—H51C	0.9800
C2—C21	1.532 (4)	C52—H52A	0.9800
C2—C22	1.540 (4)	C52—H52B	0.9800
C2—C23	1.543 (4)	C52—H52C	0.9800
C21—H21A	0.9800	C6—C61	1.520 (5)
C21—H21B	0.9800	C6—C62	1.524 (5)
C21—H21C	0.9800	C6—C63	1.547 (5)
C22—H22A	0.9800	C61—H61A	0.9800
C22—H22B	0.9800	C61—H61B	0.9800
C22—H22C	0.9800	C61—H61C	0.9800
C23—H23A	0.9800	C62—H62A	0.9800
C23—H23B	0.9800	C62—H62B	0.9800
C23—H23C	0.9800	C62—H62C	0.9800
C3—C31	1.534 (4)	C63—H63A	0.9800
C3—C32	1.541 (4)	C63—H63B	0.9800
C3—H3	1.0000	C63—H63C	0.9800
			
Cl1—Pd1—Cl2	174.61 (3)	H31A—C31—H31B	109.5
Cl1—Pd1—Se1	86.97 (2)	C3—C31—H31C	109.5
Cl2—Pd1—Se1	92.46 (2)	H31A—C31—H31C	109.5
Cl1—Pd1—Se2	99.50 (2)	H31B—C31—H31C	109.5
Cl2—Pd1—Se2	80.81 (2)	C3—C32—H32A	109.5
Se1—Pd1—Se2	172.772 (14)	C3—C32—H32B	109.5
P1—Se1—Pd1	107.78 (2)	H32A—C32—H32B	109.5
P2—Se2—Pd1	117.93 (3)	C3—C32—H32C	109.5
C3—P1—C2	107.01 (13)	H32A—C32—H32C	109.5
C3—P1—C1	113.37 (13)	H32B—C32—H32C	109.5
C2—P1—C1	112.67 (13)	C41—C4—C43	108.2 (3)
C3—P1—Se1	109.18 (10)	C41—C4—C42	109.0 (3)
C2—P1—Se1	111.22 (9)	C43—C4—C42	108.1 (3)
C1—P1—Se1	103.40 (10)	C41—C4—P2	107.4 (2)
C5—P2—C6	109.22 (16)	C43—C4—P2	111.2 (2)
C5—P2—C4	109.91 (16)	C42—C4—P2	112.8 (3)
C6—P2—C4	112.17 (16)	C4—C41—H41A	109.5
C5—P2—Se2	113.08 (11)	C4—C41—H41B	109.5
C6—P2—Se2	109.66 (11)	H41A—C41—H41B	109.5
C4—P2—Se2	102.73 (11)	C4—C41—H41C	109.5
C11—C1—C13	109.9 (2)	H41A—C41—H41C	109.5
C11—C1—C12	107.5 (2)	H41B—C41—H41C	109.5
C13—C1—C12	107.7 (3)	C4—C42—H42A	109.5
C11—C1—P1	109.5 (2)	C4—C42—H42B	109.5
C13—C1—P1	112.4 (2)	H42A—C42—H42B	109.5
C12—C1—P1	109.6 (2)	C4—C42—H42C	109.5
C1—C11—H11A	109.5	H42A—C42—H42C	109.5
C1—C11—H11B	109.5	H42B—C42—H42C	109.5
H11A—C11—H11B	109.5	C4—C43—H43A	109.5
C1—C11—H11C	109.5	C4—C43—H43B	109.5
H11A—C11—H11C	109.5	H43A—C43—H43B	109.5
H11B—C11—H11C	109.5	C4—C43—H43C	109.5
C1—C12—H12A	109.5	H43A—C43—H43C	109.5
C1—C12—H12B	109.5	H43B—C43—H43C	109.5
H12A—C12—H12B	109.5	C52—C5—C51	109.6 (3)
C1—C12—H12C	109.5	C52—C5—P2	114.2 (2)
H12A—C12—H12C	109.5	C51—C5—P2	116.3 (2)
H12B—C12—H12C	109.5	C52—C5—H5	105.2
C1—C13—H13A	109.5	C51—C5—H5	105.2
C1—C13—H13B	109.5	P2—C5—H5	105.2
H13A—C13—H13B	109.5	C5—C51—H51A	109.5
C1—C13—H13C	109.5	C5—C51—H51B	109.5
H13A—C13—H13C	109.5	H51A—C51—H51B	109.5
H13B—C13—H13C	109.5	C5—C51—H51C	109.5
C21—C2—C22	107.9 (2)	H51A—C51—H51C	109.5
C21—C2—C23	108.4 (2)	H51B—C51—H51C	109.5
C22—C2—C23	109.5 (2)	C5—C52—H52A	109.5
C21—C2—P1	111.1 (2)	C5—C52—H52B	109.5
C22—C2—P1	108.51 (19)	H52A—C52—H52B	109.5
C23—C2—P1	111.4 (2)	C5—C52—H52C	109.5
C2—C21—H21A	109.5	H52A—C52—H52C	109.5
C2—C21—H21B	109.5	H52B—C52—H52C	109.5
H21A—C21—H21B	109.5	C61—C6—C62	108.3 (3)
C2—C21—H21C	109.5	C61—C6—C63	109.1 (3)
H21A—C21—H21C	109.5	C62—C6—C63	107.3 (3)
H21B—C21—H21C	109.5	C61—C6—P2	113.4 (3)
C2—C22—H22A	109.5	C62—C6—P2	109.3 (2)
C2—C22—H22B	109.5	C63—C6—P2	109.3 (2)
H22A—C22—H22B	109.5	C6—C61—H61A	109.5
C2—C22—H22C	109.5	C6—C61—H61B	109.5
H22A—C22—H22C	109.5	H61A—C61—H61B	109.5
H22B—C22—H22C	109.5	C6—C61—H61C	109.5
C2—C23—H23A	109.5	H61A—C61—H61C	109.5
C2—C23—H23B	109.5	H61B—C61—H61C	109.5
H23A—C23—H23B	109.5	C6—C62—H62A	109.5
C2—C23—H23C	109.5	C6—C62—H62B	109.5
H23A—C23—H23C	109.5	H62A—C62—H62B	109.5
H23B—C23—H23C	109.5	C6—C62—H62C	109.5
C31—C3—C32	109.7 (2)	H62A—C62—H62C	109.5
C31—C3—P1	112.9 (2)	H62B—C62—H62C	109.5
C32—C3—P1	118.2 (2)	C6—C63—H63A	109.5
C31—C3—H3	104.9	C6—C63—H63B	109.5
C32—C3—H3	104.9	H63A—C63—H63B	109.5
P1—C3—H3	104.9	C6—C63—H63C	109.5
C3—C31—H31A	109.5	H63A—C63—H63C	109.5
C3—C31—H31B	109.5	H63B—C63—H63C	109.5
			
Cl1—Pd1—Se1—P1	−115.18 (3)	C1—P1—C3—C31	59.3 (3)
Cl2—Pd1—Se1—P1	70.19 (3)	Se1—P1—C3—C31	−55.4 (2)
Cl1—Pd1—Se2—P2	20.17 (3)	C2—P1—C3—C32	54.2 (3)
Cl2—Pd1—Se2—P2	−165.29 (3)	C1—P1—C3—C32	−70.6 (3)
Pd1—Se1—P1—C3	−74.49 (10)	Se1—P1—C3—C32	174.7 (2)
Pd1—Se1—P1—C2	43.37 (10)	C5—P2—C4—C41	−45.7 (3)
Pd1—Se1—P1—C1	164.54 (9)	C6—P2—C4—C41	−167.4 (2)
Pd1—Se2—P2—C5	−53.52 (13)	Se2—P2—C4—C41	74.9 (2)
Pd1—Se2—P2—C6	68.63 (12)	C5—P2—C4—C43	−163.9 (2)
Pd1—Se2—P2—C4	−171.93 (11)	C6—P2—C4—C43	74.4 (3)
C3—P1—C1—C11	169.5 (2)	Se2—P2—C4—C43	−43.3 (3)
C2—P1—C1—C11	47.7 (2)	C5—P2—C4—C42	74.4 (3)
Se1—P1—C1—C11	−72.4 (2)	C6—P2—C4—C42	−47.3 (3)
C3—P1—C1—C13	47.0 (3)	Se2—P2—C4—C42	−165.0 (2)
C2—P1—C1—C13	−74.7 (2)	C6—P2—C5—C52	−154.2 (3)
Se1—P1—C1—C13	165.09 (19)	C4—P2—C5—C52	82.4 (3)
C3—P1—C1—C12	−72.8 (2)	Se2—P2—C5—C52	−31.8 (3)
C2—P1—C1—C12	165.5 (2)	C6—P2—C5—C51	76.6 (3)
Se1—P1—C1—C12	45.3 (2)	C4—P2—C5—C51	−46.9 (3)
C3—P1—C2—C21	158.0 (2)	Se2—P2—C5—C51	−161.0 (2)
C1—P1—C2—C21	−76.8 (2)	C5—P2—C6—C61	−43.2 (3)
Se1—P1—C2—C21	38.8 (2)	C4—P2—C6—C61	78.9 (3)
C3—P1—C2—C22	39.5 (2)	Se2—P2—C6—C61	−167.7 (2)
C1—P1—C2—C22	164.73 (19)	C5—P2—C6—C62	77.6 (3)
Se1—P1—C2—C22	−79.7 (2)	C4—P2—C6—C62	−160.2 (2)
C3—P1—C2—C23	−81.1 (2)	Se2—P2—C6—C62	−46.8 (3)
C1—P1—C2—C23	44.2 (2)	C5—P2—C6—C63	−165.2 (2)
Se1—P1—C2—C23	159.75 (18)	C4—P2—C6—C63	−43.1 (3)
C2—P1—C3—C31	−175.8 (2)	Se2—P2—C6—C63	70.4 (3)

### (Di-tert-butylisopropylphosphine selenide-κSe)dichloridopalladium(II) (2) Hydrogen-bond geometry (Å, º)

**Table:**

*D*—H···*A*	*D*—H	H···*A*	*D*···*A*	*D*—H···*A*
C11—H11*B*···Se1	0.98	3.08	3.633 (3)	117
C12—H12*C*···Se1	0.98	2.72	3.282 (3)	117
C21—H21*C*···Cl1	0.98	2.77	3.741 (3)	170
C21—H21*C*···Se1	0.98	2.92	3.478 (3)	117
C3—H3···Cl2	1.00	2.77	3.608 (3)	142
C31—H31*B*···Se1	0.98	2.99	3.606 (3)	122
C41—H41*C*···Se2	0.98	3.08	3.632 (4)	117
C43—H43*A*···Se2	0.98	2.67	3.300 (4)	123
C5—H5···Cl1	1.00	2.83	3.484 (4)	124
C51—H51*C*···Cl1^i^	0.98	2.76	3.642 (3)	150
C52—H52*B*···Cl1	0.98	2.81	3.439 (4)	123
C52—H52*B*···Se2	0.98	2.97	3.507 (4)	116
C62—H62*B*···Se2	0.98	2.91	3.456 (4)	116
C62—H62*C*···Cl1	0.98	2.82	3.552 (4)	132
C63—H63*A*···Se1^ii^	0.98	3.05	3.948 (4)	153
C63—H63*A*···Pd1^ii^	0.98	2.87	3.793 (4)	158

Symmetry codes: (i) −*x*+1, −*y*+1, −*z*+1; (ii) *x*, −*y*+3/2, *z*−1/2.

### Dibromido(di-tert-butylisopropylphosphine selenide-κSe)palladium(II) (3) Crystal data

**Table:**

[PdBr_2_(C_11_H_25_PSe)_2_]	*F*(000) = 1584
*M**_r_* = 800.70	*D*_x_ = 1.781 Mg m^−^^3^
Monoclinic, *P*2_1_/*c*	Mo *K*α radiation, λ = 0.71073 Å
*a* = 15.3561 (6) Å	Cell parameters from 11858 reflections
*b* = 13.4695 (4) Å	θ = 2.1–30.8°
*c* = 16.1371 (6) Å	µ = 5.85 mm^−^^1^
β = 116.558 (5)°	*T* = 100 K
*V* = 2985.6 (2) Å^3^	Plate, dichroic orange yellow
*Z* = 4	0.18 × 0.05 × 0.02 mm

### Dibromido(di-tert-butylisopropylphosphine selenide-κSe)palladium(II) (3) Data collection

**Table:**

Oxford Diffraction Xcalibur, Eos diffractometer	7402 independent reflections
Radiation source: fine-focus sealed tube	5854 reflections with *I* > 2σ(*I*)
Graphite monochromator	*R*_int_ = 0.104
Detector resolution: 16.1419 pixels mm^-1^	θ_max_ = 28.3°, θ_min_ = 2.1°
ω scans	*h* = −20→20
Absorption correction: multi-scan (CrysAlisPro; Rigaku OD, 2012)	*k* = −17→17
*T*_min_ = 0.419, *T*_max_ = 0.892	*l* = −21→21
120046 measured reflections	

### Dibromido(di-tert-butylisopropylphosphine selenide-κSe)palladium(II) (3) Refinement

**Table:**

Refinement on *F*^2^	Primary atom site location: structure-invariant direct methods
Least-squares matrix: full	Secondary atom site location: difference Fourier map
*R*[*F*^2^ > 2σ(*F*^2^)] = 0.039	Hydrogen site location: inferred from neighbouring sites
*wR*(*F*^2^) = 0.083	H-atom parameters constrained
*S* = 1.04	*w* = 1/[σ^2^(*F*_o_^2^) + (0.0262*P*)^2^ + 9.8278*P*] where *P* = (*F*_o_^2^ + 2*F*_c_^2^)/3
7402 reflections	(Δ/σ)_max_ = 0.001
278 parameters	Δρ_max_ = 2.68 e Å^−^^3^
0 restraints	Δρ_min_ = −1.32 e Å^−^^3^

### Dibromido(di-tert-butylisopropylphosphine selenide-κSe)palladium(II) (3) Fractional atomic coordinates and isotropic or equivalent isotropic displacement parameters (Å^2^)

**Table:**

	*x*	*y*	*z*	*U*_iso_*/*U*_eq_	
Pd1	0.21918 (2)	0.75073 (2)	0.43434 (2)	0.01287 (7)	
Br1	0.27518 (3)	0.58168 (3)	0.43222 (3)	0.02354 (11)	
Br2	0.17265 (3)	0.91841 (3)	0.45811 (3)	0.01765 (9)	
Se1	0.08502 (3)	0.67520 (3)	0.45392 (3)	0.01525 (9)	
Se2	0.35225 (3)	0.84539 (3)	0.42600 (3)	0.02064 (10)	
P1	−0.05189 (7)	0.71084 (8)	0.33294 (7)	0.0120 (2)	
P2	0.44090 (8)	0.77472 (9)	0.36726 (8)	0.0192 (2)	
C1	−0.1407 (3)	0.6195 (3)	0.3414 (3)	0.0182 (9)	
C11	−0.1193 (3)	0.5150 (3)	0.3172 (3)	0.0240 (10)	
H11A	−0.156840	0.466156	0.332898	0.036*	
H11B	−0.049659	0.500810	0.352519	0.036*	
H11C	−0.137787	0.511447	0.250767	0.036*	
C12	−0.1279 (3)	0.6164 (4)	0.4421 (3)	0.0257 (10)	
H12A	−0.175427	0.570368	0.445869	0.039*	
H12B	−0.138252	0.682963	0.460610	0.039*	
H12C	−0.061899	0.593931	0.483685	0.039*	
C13	−0.2468 (3)	0.6477 (4)	0.2783 (3)	0.0261 (10)	
H13A	−0.256585	0.652786	0.214177	0.039*	
H13B	−0.261527	0.711689	0.298098	0.039*	
H13C	−0.290132	0.596587	0.282483	0.039*	
C2	−0.0403 (3)	0.6968 (3)	0.2225 (3)	0.0161 (8)	
C21	0.0218 (3)	0.6064 (3)	0.2255 (3)	0.0207 (9)	
H21A	0.030884	0.604683	0.169173	0.031*	
H21B	−0.011140	0.545654	0.229574	0.031*	
H21C	0.085417	0.611226	0.279842	0.031*	
C22	0.0110 (3)	0.7902 (3)	0.2100 (3)	0.0200 (9)	
H22A	0.073163	0.799598	0.265212	0.030*	
H22B	−0.030508	0.848400	0.201391	0.030*	
H22C	0.022835	0.781899	0.155587	0.030*	
C23	−0.1407 (3)	0.6866 (4)	0.1375 (3)	0.0223 (9)	
H23A	−0.132414	0.687864	0.080729	0.033*	
H23B	−0.182575	0.741859	0.136722	0.033*	
H23C	−0.170713	0.623666	0.141251	0.033*	
C3	−0.0841 (3)	0.8417 (3)	0.3399 (3)	0.0160 (8)	
H3	−0.027467	0.880820	0.342719	0.019*	
C31	−0.0892 (4)	0.8665 (4)	0.4308 (3)	0.0270 (10)	
H31A	−0.086566	0.938682	0.439388	0.041*	
H31B	−0.034074	0.835710	0.482964	0.041*	
H31C	−0.150280	0.840984	0.427894	0.041*	
C32	−0.1734 (3)	0.8863 (3)	0.2577 (3)	0.0235 (10)	
H32A	−0.232344	0.851460	0.250726	0.035*	
H32B	−0.165869	0.878896	0.200851	0.035*	
H32C	−0.178865	0.956897	0.269306	0.035*	
C4	0.5400 (3)	0.8700 (4)	0.3894 (4)	0.0290 (11)	
C41	0.6093 (4)	0.8676 (5)	0.4926 (4)	0.0457 (15)	
H41A	0.656464	0.921832	0.507720	0.069*	
H41B	0.643857	0.803925	0.508191	0.069*	
H41C	0.572109	0.875352	0.528170	0.069*	
C42	0.5960 (5)	0.8510 (5)	0.3324 (5)	0.0551 (17)	
H42A	0.642049	0.905379	0.342308	0.083*	
H42B	0.550002	0.847280	0.266478	0.083*	
H42C	0.631709	0.788233	0.351925	0.083*	
C43	0.4983 (4)	0.9747 (4)	0.3662 (4)	0.0401 (13)	
H43A	0.460652	0.989064	0.400297	0.060*	
H43B	0.455855	0.979673	0.299479	0.060*	
H43C	0.551666	1.022634	0.383809	0.060*	
C5	0.4953 (3)	0.6558 (3)	0.4234 (3)	0.0241 (10)	
H5	0.441791	0.605898	0.394996	0.029*	
C51	0.5784 (4)	0.6157 (4)	0.4052 (4)	0.0397 (14)	
H51A	0.637091	0.655526	0.439550	0.060*	
H51B	0.559936	0.619523	0.338762	0.060*	
H51C	0.591391	0.546434	0.425604	0.060*	
C52	0.5245 (4)	0.6523 (4)	0.5272 (3)	0.0385 (13)	
H52A	0.538156	0.583456	0.548987	0.058*	
H52B	0.471159	0.678218	0.538551	0.058*	
H52C	0.582848	0.692822	0.560611	0.058*	
C6	0.3644 (4)	0.7522 (4)	0.2406 (3)	0.0287 (11)	
C61	0.4136 (5)	0.6876 (5)	0.1969 (4)	0.0529 (18)	
H61A	0.427936	0.622348	0.226977	0.079*	
H61B	0.474323	0.719151	0.204913	0.079*	
H61C	0.370228	0.679586	0.130734	0.079*	
C62	0.2698 (4)	0.7024 (5)	0.2255 (4)	0.0472 (15)	
H62A	0.227124	0.696604	0.158951	0.071*	
H62B	0.237428	0.742320	0.254450	0.071*	
H62C	0.283632	0.636084	0.253488	0.071*	
C63	0.3361 (4)	0.8524 (4)	0.1882 (4)	0.0455 (15)	
H63A	0.285766	0.841135	0.124748	0.068*	
H63B	0.393630	0.881876	0.186418	0.068*	
H63C	0.311119	0.897575	0.220092	0.068*	

### Dibromido(di-tert-butylisopropylphosphine selenide-κSe)palladium(II) (3) Atomic displacement parameters (Å^2^)

**Table:**

	*U*^11^	*U*^22^	*U*^33^	*U*^12^	*U*^13^	*U*^23^
Pd1	0.01204 (15)	0.01246 (14)	0.01323 (14)	0.00053 (11)	0.00486 (12)	−0.00056 (12)
Br1	0.0192 (2)	0.0154 (2)	0.0342 (3)	0.00275 (17)	0.0103 (2)	−0.00115 (18)
Br2	0.0166 (2)	0.01380 (19)	0.0229 (2)	0.00086 (16)	0.00917 (17)	−0.00232 (16)
Se1	0.0140 (2)	0.0174 (2)	0.01274 (19)	−0.00010 (16)	0.00447 (16)	0.00418 (16)
Se2	0.0205 (2)	0.0203 (2)	0.0266 (2)	−0.00533 (18)	0.01539 (19)	−0.00853 (18)
P1	0.0118 (5)	0.0126 (5)	0.0118 (5)	0.0001 (4)	0.0054 (4)	0.0009 (4)
P2	0.0163 (5)	0.0234 (6)	0.0195 (6)	0.0023 (4)	0.0094 (5)	−0.0018 (4)
C1	0.018 (2)	0.015 (2)	0.022 (2)	−0.0022 (17)	0.0094 (18)	0.0017 (17)
C11	0.025 (2)	0.014 (2)	0.036 (3)	−0.0052 (18)	0.016 (2)	0.0000 (19)
C12	0.028 (3)	0.030 (3)	0.028 (2)	−0.003 (2)	0.020 (2)	0.005 (2)
C13	0.018 (2)	0.025 (2)	0.034 (3)	−0.0013 (19)	0.011 (2)	0.001 (2)
C2	0.016 (2)	0.020 (2)	0.0115 (19)	−0.0045 (17)	0.0054 (16)	−0.0004 (16)
C21	0.022 (2)	0.024 (2)	0.016 (2)	0.0012 (18)	0.0093 (19)	−0.0020 (17)
C22	0.018 (2)	0.029 (2)	0.013 (2)	−0.0025 (18)	0.0063 (17)	−0.0001 (17)
C23	0.018 (2)	0.029 (2)	0.016 (2)	−0.0013 (19)	0.0037 (18)	−0.0045 (18)
C3	0.018 (2)	0.014 (2)	0.016 (2)	−0.0003 (16)	0.0069 (17)	0.0009 (16)
C31	0.033 (3)	0.022 (2)	0.031 (3)	0.005 (2)	0.019 (2)	−0.001 (2)
C32	0.023 (2)	0.018 (2)	0.028 (2)	0.0042 (18)	0.011 (2)	0.0040 (18)
C4	0.021 (2)	0.031 (3)	0.040 (3)	0.000 (2)	0.018 (2)	0.002 (2)
C41	0.025 (3)	0.045 (4)	0.046 (3)	−0.013 (3)	−0.003 (3)	0.007 (3)
C42	0.044 (4)	0.056 (4)	0.082 (5)	−0.005 (3)	0.044 (4)	0.000 (4)
C43	0.034 (3)	0.034 (3)	0.053 (4)	−0.002 (2)	0.020 (3)	0.013 (3)
C5	0.025 (2)	0.025 (2)	0.024 (2)	0.0012 (19)	0.013 (2)	−0.0006 (19)
C51	0.035 (3)	0.042 (3)	0.045 (3)	0.024 (3)	0.020 (3)	0.019 (3)
C52	0.045 (3)	0.039 (3)	0.031 (3)	−0.002 (3)	0.016 (3)	0.008 (2)
C6	0.028 (3)	0.039 (3)	0.019 (2)	0.005 (2)	0.010 (2)	−0.002 (2)
C61	0.050 (4)	0.078 (5)	0.026 (3)	0.023 (3)	0.013 (3)	−0.009 (3)
C62	0.045 (4)	0.061 (4)	0.031 (3)	−0.015 (3)	0.013 (3)	−0.015 (3)
C63	0.045 (4)	0.046 (4)	0.031 (3)	0.013 (3)	0.004 (3)	0.004 (3)

### Dibromido(di-tert-butylisopropylphosphine selenide-κSe)palladium(II) (3) Geometric parameters (Å, º)

**Table:**

Pd1—Br1	2.4395 (5)	C31—H31A	0.9800
Pd1—Se1	2.4416 (5)	C31—H31B	0.9800
Pd1—Br2	2.4503 (5)	C31—H31C	0.9800
Pd1—Se2	2.4628 (5)	C32—H32A	0.9800
Se1—P1	2.1890 (11)	C32—H32B	0.9800
Se2—P2	2.1935 (12)	C32—H32C	0.9800
P1—C3	1.847 (4)	C4—C41	1.524 (7)
P1—C2	1.879 (4)	C4—C43	1.524 (7)
P1—C1	1.886 (4)	C4—C42	1.536 (7)
P2—C5	1.846 (5)	C41—H41A	0.9800
P2—C6	1.871 (5)	C41—H41B	0.9800
P2—C4	1.899 (5)	C41—H41C	0.9800
C1—C11	1.534 (6)	C42—H42A	0.9800
C1—C13	1.534 (6)	C42—H42B	0.9800
C1—C12	1.549 (6)	C42—H42C	0.9800
C11—H11A	0.9800	C43—H43A	0.9800
C11—H11B	0.9800	C43—H43B	0.9800
C11—H11C	0.9800	C43—H43C	0.9800
C12—H12A	0.9800	C5—C52	1.529 (6)
C12—H12B	0.9800	C5—C51	1.530 (6)
C12—H12C	0.9800	C5—H5	1.0000
C13—H13A	0.9800	C51—H51A	0.9800
C13—H13B	0.9800	C51—H51B	0.9800
C13—H13C	0.9800	C51—H51C	0.9800
C2—C21	1.534 (6)	C52—H52A	0.9800
C2—C22	1.544 (6)	C52—H52B	0.9800
C2—C23	1.544 (6)	C52—H52C	0.9800
C21—H21A	0.9800	C6—C61	1.517 (7)
C21—H21B	0.9800	C6—C62	1.517 (7)
C21—H21C	0.9800	C6—C63	1.549 (7)
C22—H22A	0.9800	C61—H61A	0.9800
C22—H22B	0.9800	C61—H61B	0.9800
C22—H22C	0.9800	C61—H61C	0.9800
C23—H23A	0.9800	C62—H62A	0.9800
C23—H23B	0.9800	C62—H62B	0.9800
C23—H23C	0.9800	C62—H62C	0.9800
C3—C32	1.541 (6)	C63—H63A	0.9800
C3—C31	1.541 (6)	C63—H63B	0.9800
C3—H3	1.0000	C63—H63C	0.9800
			
Br1—Pd1—Se1	86.332 (18)	H31A—C31—H31B	109.5
Br1—Pd1—Br2	172.66 (2)	C3—C31—H31C	109.5
Se1—Pd1—Br2	92.395 (17)	H31A—C31—H31C	109.5
Br1—Pd1—Se2	100.149 (18)	H31B—C31—H31C	109.5
Se1—Pd1—Se2	172.54 (2)	C3—C32—H32A	109.5
Br2—Pd1—Se2	80.706 (17)	C3—C32—H32B	109.5
P1—Se1—Pd1	108.83 (3)	H32A—C32—H32B	109.5
P2—Se2—Pd1	119.30 (4)	C3—C32—H32C	109.5
C3—P1—C2	107.14 (19)	H32A—C32—H32C	109.5
C3—P1—C1	113.37 (19)	H32B—C32—H32C	109.5
C2—P1—C1	112.42 (19)	C41—C4—C43	107.5 (5)
C3—P1—Se1	109.36 (14)	C41—C4—C42	110.3 (5)
C2—P1—Se1	111.31 (13)	C43—C4—C42	107.1 (5)
C1—P1—Se1	103.26 (14)	C41—C4—P2	107.4 (3)
C5—P2—C6	108.7 (2)	C43—C4—P2	111.4 (3)
C5—P2—C4	110.2 (2)	C42—C4—P2	113.0 (4)
C6—P2—C4	112.0 (2)	C4—C41—H41A	109.5
C5—P2—Se2	113.36 (14)	C4—C41—H41B	109.5
C6—P2—Se2	109.52 (16)	H41A—C41—H41B	109.5
C4—P2—Se2	103.04 (16)	C4—C41—H41C	109.5
C11—C1—C13	110.1 (4)	H41A—C41—H41C	109.5
C11—C1—C12	107.5 (4)	H41B—C41—H41C	109.5
C13—C1—C12	107.5 (4)	C4—C42—H42A	109.5
C11—C1—P1	109.7 (3)	C4—C42—H42B	109.5
C13—C1—P1	112.2 (3)	H42A—C42—H42B	109.5
C12—C1—P1	109.7 (3)	C4—C42—H42C	109.5
C1—C11—H11A	109.5	H42A—C42—H42C	109.5
C1—C11—H11B	109.5	H42B—C42—H42C	109.5
H11A—C11—H11B	109.5	C4—C43—H43A	109.5
C1—C11—H11C	109.5	C4—C43—H43B	109.5
H11A—C11—H11C	109.5	H43A—C43—H43B	109.5
H11B—C11—H11C	109.5	C4—C43—H43C	109.5
C1—C12—H12A	109.5	H43A—C43—H43C	109.5
C1—C12—H12B	109.5	H43B—C43—H43C	109.5
H12A—C12—H12B	109.5	C52—C5—C51	109.8 (4)
C1—C12—H12C	109.5	C52—C5—P2	114.3 (3)
H12A—C12—H12C	109.5	C51—C5—P2	116.4 (3)
H12B—C12—H12C	109.5	C52—C5—H5	105.0
C1—C13—H13A	109.5	C51—C5—H5	105.0
C1—C13—H13B	109.5	P2—C5—H5	105.0
H13A—C13—H13B	109.5	C5—C51—H51A	109.5
C1—C13—H13C	109.5	C5—C51—H51B	109.5
H13A—C13—H13C	109.5	H51A—C51—H51B	109.5
H13B—C13—H13C	109.5	C5—C51—H51C	109.5
C21—C2—C22	107.8 (3)	H51A—C51—H51C	109.5
C21—C2—C23	108.5 (3)	H51B—C51—H51C	109.5
C22—C2—C23	108.8 (3)	C5—C52—H52A	109.5
C21—C2—P1	111.7 (3)	C5—C52—H52B	109.5
C22—C2—P1	108.2 (3)	H52A—C52—H52B	109.5
C23—C2—P1	111.7 (3)	C5—C52—H52C	109.5
C2—C21—H21A	109.5	H52A—C52—H52C	109.5
C2—C21—H21B	109.5	H52B—C52—H52C	109.5
H21A—C21—H21B	109.5	C61—C6—C62	108.2 (5)
C2—C21—H21C	109.5	C61—C6—C63	108.7 (5)
H21A—C21—H21C	109.5	C62—C6—C63	106.5 (5)
H21B—C21—H21C	109.5	C61—C6—P2	113.6 (4)
C2—C22—H22A	109.5	C62—C6—P2	109.6 (3)
C2—C22—H22B	109.5	C63—C6—P2	110.0 (4)
H22A—C22—H22B	109.5	C6—C61—H61A	109.5
C2—C22—H22C	109.5	C6—C61—H61B	109.5
H22A—C22—H22C	109.5	H61A—C61—H61B	109.5
H22B—C22—H22C	109.5	C6—C61—H61C	109.5
C2—C23—H23A	109.5	H61A—C61—H61C	109.5
C2—C23—H23B	109.5	H61B—C61—H61C	109.5
H23A—C23—H23B	109.5	C6—C62—H62A	109.5
C2—C23—H23C	109.5	C6—C62—H62B	109.5
H23A—C23—H23C	109.5	H62A—C62—H62B	109.5
H23B—C23—H23C	109.5	C6—C62—H62C	109.5
C32—C3—C31	109.2 (4)	H62A—C62—H62C	109.5
C32—C3—P1	118.6 (3)	H62B—C62—H62C	109.5
C31—C3—P1	113.2 (3)	C6—C63—H63A	109.5
C32—C3—H3	104.8	C6—C63—H63B	109.5
C31—C3—H3	104.8	H63A—C63—H63B	109.5
P1—C3—H3	104.8	C6—C63—H63C	109.5
C3—C31—H31A	109.5	H63A—C63—H63C	109.5
C3—C31—H31B	109.5	H63B—C63—H63C	109.5
			
Br1—Pd1—Se1—P1	−116.19 (3)	C1—P1—C3—C32	−70.6 (4)
Br2—Pd1—Se1—P1	71.05 (3)	Se1—P1—C3—C32	174.8 (3)
Br1—Pd1—Se2—P2	23.37 (4)	C2—P1—C3—C31	−176.0 (3)
Br2—Pd1—Se2—P2	−164.03 (4)	C1—P1—C3—C31	59.4 (4)
Pd1—Se1—P1—C3	−74.90 (14)	Se1—P1—C3—C31	−55.2 (3)
Pd1—Se1—P1—C2	43.29 (15)	C5—P2—C4—C41	−47.9 (4)
Pd1—Se1—P1—C1	164.12 (14)	C6—P2—C4—C41	−169.0 (4)
Pd1—Se2—P2—C5	−54.43 (18)	Se2—P2—C4—C41	73.4 (4)
Pd1—Se2—P2—C6	67.15 (18)	C5—P2—C4—C43	−165.3 (4)
Pd1—Se2—P2—C4	−173.53 (16)	C6—P2—C4—C43	73.5 (4)
C3—P1—C1—C11	169.6 (3)	Se2—P2—C4—C43	−44.1 (4)
C2—P1—C1—C11	47.9 (4)	C5—P2—C4—C42	74.0 (4)
Se1—P1—C1—C11	−72.2 (3)	C6—P2—C4—C42	−47.1 (5)
C3—P1—C1—C13	47.0 (4)	Se2—P2—C4—C42	−164.7 (4)
C2—P1—C1—C13	−74.7 (4)	C6—P2—C5—C52	−156.7 (4)
Se1—P1—C1—C13	165.2 (3)	C4—P2—C5—C52	80.3 (4)
C3—P1—C1—C12	−72.5 (3)	Se2—P2—C5—C52	−34.6 (4)
C2—P1—C1—C12	165.8 (3)	C6—P2—C5—C51	73.6 (4)
Se1—P1—C1—C12	45.7 (3)	C4—P2—C5—C51	−49.4 (4)
C3—P1—C2—C21	158.5 (3)	Se2—P2—C5—C51	−164.3 (3)
C1—P1—C2—C21	−76.3 (3)	C5—P2—C6—C61	−45.5 (5)
Se1—P1—C2—C21	38.9 (3)	C4—P2—C6—C61	76.5 (5)
C3—P1—C2—C22	40.0 (3)	Se2—P2—C6—C61	−169.9 (4)
C1—P1—C2—C22	165.2 (3)	C5—P2—C6—C62	75.6 (4)
Se1—P1—C2—C22	−79.5 (3)	C4—P2—C6—C62	−162.4 (4)
C3—P1—C2—C23	−79.7 (3)	Se2—P2—C6—C62	−48.7 (4)
C1—P1—C2—C23	45.5 (4)	C5—P2—C6—C63	−167.7 (4)
Se1—P1—C2—C23	160.8 (3)	C4—P2—C6—C63	−45.7 (4)
C2—P1—C3—C32	54.0 (4)	Se2—P2—C6—C63	68.0 (4)

### Dibromido(di-tert-butylisopropylphosphine selenide-κSe)palladium(II) (3) Hydrogen-bond geometry (Å, º)

**Table:**

*D*—H···*A*	*D*—H	H···*A*	*D*···*A*	*D*—H···*A*
C11—H11*B*···Se1	0.98	3.07	3.630 (4)	117
C12—H12*C*···Se1	0.98	2.74	3.286 (5)	116
C21—H21*C*···Br1	0.98	2.88	3.843 (4)	168
C21—H21*C*···Se1	0.98	2.94	3.491 (4)	117
C22—H22*A*···Pd1	0.98	2.73	3.641 (4)	155
C3—H3···Br2	1.00	2.84	3.678 (4)	142
C31—H31*B*···Se1	0.98	3.00	3.612 (5)	122
C41—H41*C*···Se2	0.98	3.05	3.610 (6)	118
C43—H43*A*···Se2	0.98	2.70	3.307 (5)	120
C5—H5···Br1	1.00	2.90	3.588 (5)	127
C51—H51*C*···Br1^i^	0.98	2.87	3.706 (5)	144
C52—H52*B*···Br1	0.98	3.01	3.558 (6)	116
C52—H52*B*···Se2	0.98	2.96	3.545 (6)	120
C62—H62*B*···Se2	0.98	2.89	3.481 (6)	120
C62—H62*C*···Br1	0.98	3.03	3.678 (6)	124
C63—H63*A*···Se1^ii^	0.98	3.09	4.032 (6)	162
C63—H63*A*···Pd1^ii^	0.98	3.03	3.918 (6)	151

Symmetry codes: (i) −*x*+1, −*y*+1, −*z*+1; (ii) *x*, −*y*+3/2, *z*−1/2.

### Dibromido(di-tert-butylisopropylphosphine sulfide-κS)palladium(II) (4) Crystal data

**Table:**

[PdBr_2_(C_11_H_25_PS)_2_]	*F*(000) = 720
*M**_r_* = 706.90	*D*_x_ = 1.604 Mg m^−^^3^
Monoclinic, *P*2_1_/*n*	Mo *K*α radiation, λ = 0.71073 Å
*a* = 7.8595 (3) Å	Cell parameters from 9329 reflections
*b* = 17.5019 (6) Å	θ = 2.2–30.8°
*c* = 10.6740 (3) Å	µ = 3.63 mm^−^^1^
β = 94.551 (3)°	*T* = 100 K
*V* = 1463.65 (9) Å^3^	Prism, red
*Z* = 2	0.2 × 0.08 × 0.05 mm

### Dibromido(di-tert-butylisopropylphosphine sulfide-κS)palladium(II) (4) Data collection

**Table:**

Oxford Diffraction Xcalibur, Eos diffractometer	4422 independent reflections
Radiation source: Enhance (Mo) X-ray Source	3832 reflections with *I* > 2σ(*I*)
Detector resolution: 16.1419 pixels mm^-1^	*R*_int_ = 0.050
ω scans	θ_max_ = 30.9°, θ_min_ = 2.2°
Absorption correction: multi-scan (CrysAlisPro; Rigaku OD, 2012)	*h* = −10→11
*T*_min_ = 0.865, *T*_max_ = 1.000	*k* = −25→24
45160 measured reflections	*l* = −15→14

### Dibromido(di-tert-butylisopropylphosphine sulfide-κS)palladium(II) (4) Refinement

**Table:**

Refinement on *F*^2^	Primary atom site location: structure-invariant direct methods
Least-squares matrix: full	Secondary atom site location: difference Fourier map
*R*[*F*^2^ > 2σ(*F*^2^)] = 0.039	Hydrogen site location: inferred from neighbouring sites
*wR*(*F*^2^) = 0.065	H-atom parameters constrained
*S* = 1.22	*w* = 1/[σ^2^(*F*_o_^2^) + (0.0106*P*)^2^ + 2.7056*P*] where *P* = (*F*_o_^2^ + 2*F*_c_^2^)/3
4422 reflections	(Δ/σ)_max_ = 0.001
141 parameters	Δρ_max_ = 1.02 e Å^−^^3^
0 restraints	Δρ_min_ = −0.70 e Å^−^^3^

### Dibromido(di-tert-butylisopropylphosphine sulfide-κS)palladium(II) (4) Fractional atomic coordinates and isotropic or equivalent isotropic displacement parameters (Å^2^)

**Table:**

	*x*	*y*	*z*	*U*_iso_*/*U*_eq_	
Pd1	0.500000	0.500000	0.500000	0.01288 (6)	
Br1	0.42664 (4)	0.43715 (2)	0.69362 (2)	0.02184 (7)	
S1	0.50672 (9)	0.61904 (4)	0.59836 (6)	0.01631 (13)	
P1	0.69438 (9)	0.63805 (4)	0.73533 (6)	0.01672 (14)	
C1	0.6766 (5)	0.74449 (17)	0.7604 (3)	0.0303 (7)	
C2	0.9064 (4)	0.6062 (3)	0.6863 (3)	0.0394 (9)	
C3	0.6566 (4)	0.58401 (15)	0.8794 (2)	0.0177 (5)	
H3	0.643819	0.529579	0.851762	0.021*	
C11	0.4869 (5)	0.76795 (18)	0.7592 (3)	0.0370 (9)	
H11A	0.479014	0.822916	0.775306	0.055*	
H11B	0.432726	0.739706	0.824649	0.055*	
H11C	0.428534	0.756105	0.676939	0.055*	
C12	0.7681 (5)	0.76860 (19)	0.8859 (3)	0.0360 (8)	
H12A	0.767553	0.824455	0.892452	0.054*	
H12B	0.886187	0.750204	0.890819	0.054*	
H12C	0.709110	0.746614	0.954975	0.054*	
C13	0.7540 (6)	0.7875 (2)	0.6522 (3)	0.0526 (12)	
H13A	0.875993	0.775669	0.652991	0.079*	
H13B	0.739078	0.842626	0.663040	0.079*	
H13C	0.696157	0.771508	0.571718	0.079*	
C21	0.9235 (5)	0.6266 (3)	0.5471 (3)	0.0556 (13)	
H21A	1.031394	0.606305	0.520980	0.083*	
H21B	0.921907	0.682258	0.537176	0.083*	
H21C	0.828103	0.604191	0.494919	0.083*	
C22	0.9172 (5)	0.5191 (3)	0.6991 (4)	0.0543 (13)	
H22A	0.914052	0.504892	0.787721	0.082*	
H22B	1.024007	0.501008	0.667953	0.082*	
H22C	0.820267	0.495659	0.649915	0.082*	
C23	1.0582 (5)	0.6450 (3)	0.7640 (4)	0.0599 (14)	
H23A	1.053169	0.632649	0.853171	0.090*	
H23B	1.051007	0.700531	0.752609	0.090*	
H23C	1.166053	0.626419	0.735231	0.090*	
C31	0.7987 (4)	0.58306 (19)	0.9882 (3)	0.0278 (7)	
H31A	0.763606	0.550379	1.056143	0.042*	
H31B	0.818221	0.635141	1.019900	0.042*	
H31C	0.904217	0.563072	0.957619	0.042*	
C32	0.4866 (4)	0.60471 (18)	0.9302 (3)	0.0262 (6)	
H32A	0.493743	0.656222	0.966213	0.039*	
H32B	0.460738	0.567997	0.995351	0.039*	
H32C	0.395990	0.603216	0.861626	0.039*	

### Dibromido(di-tert-butylisopropylphosphine sulfide-κS)palladium(II) (4) Atomic displacement parameters (Å^2^)

**Table:**

	*U*^11^	*U*^22^	*U*^33^	*U*^12^	*U*^13^	*U*^23^
Pd1	0.01454 (13)	0.01546 (12)	0.00854 (12)	0.00066 (11)	0.00023 (9)	−0.00076 (10)
Br1	0.03258 (17)	0.02237 (13)	0.01082 (12)	−0.00486 (12)	0.00328 (11)	0.00052 (10)
S1	0.0192 (3)	0.0167 (3)	0.0125 (3)	0.0023 (2)	−0.0020 (2)	−0.0014 (2)
P1	0.0159 (3)	0.0239 (3)	0.0104 (3)	−0.0046 (3)	0.0015 (3)	−0.0018 (3)
C1	0.050 (2)	0.0214 (14)	0.0183 (14)	−0.0136 (14)	−0.0064 (14)	−0.0016 (11)
C2	0.0112 (15)	0.086 (3)	0.0220 (16)	0.0006 (16)	0.0038 (12)	−0.0152 (17)
C3	0.0209 (14)	0.0188 (12)	0.0136 (12)	0.0014 (10)	0.0019 (10)	0.0009 (10)
C11	0.061 (3)	0.0201 (15)	0.0269 (17)	0.0095 (15)	−0.0133 (16)	−0.0059 (12)
C12	0.055 (2)	0.0295 (16)	0.0225 (16)	−0.0164 (16)	−0.0039 (15)	−0.0035 (13)
C13	0.089 (3)	0.044 (2)	0.0234 (17)	−0.041 (2)	−0.0055 (19)	0.0074 (15)
C21	0.029 (2)	0.119 (4)	0.0205 (17)	−0.021 (2)	0.0115 (15)	−0.021 (2)
C22	0.030 (2)	0.095 (4)	0.037 (2)	0.035 (2)	−0.0038 (16)	−0.022 (2)
C23	0.0173 (18)	0.132 (5)	0.031 (2)	−0.013 (2)	0.0039 (15)	−0.020 (2)
C31	0.0313 (18)	0.0345 (17)	0.0164 (14)	0.0034 (13)	−0.0056 (12)	−0.0003 (12)
C32	0.0258 (16)	0.0356 (17)	0.0181 (14)	0.0023 (13)	0.0077 (12)	0.0040 (12)

### Dibromido(di-tert-butylisopropylphosphine sulfide-κS)palladium(II) (4) Geometric parameters (Å, º)

**Table:**

Pd1—S1^i^	2.3317 (6)	C12—H12B	0.9800
Pd1—S1	2.3317 (6)	C12—H12C	0.9800
Pd1—Br1	2.4501 (3)	C13—H13A	0.9800
Pd1—Br1^i^	2.4501 (3)	C13—H13B	0.9800
S1—P1	2.0202 (10)	C13—H13C	0.9800
P1—C3	1.849 (3)	C21—H21A	0.9800
P1—C2	1.871 (3)	C21—H21B	0.9800
P1—C1	1.889 (3)	C21—H21C	0.9800
C1—C12	1.529 (4)	C22—H22A	0.9800
C1—C13	1.544 (5)	C22—H22B	0.9800
C1—C11	1.546 (5)	C22—H22C	0.9800
C2—C22	1.532 (6)	C23—H23A	0.9800
C2—C21	1.544 (5)	C23—H23B	0.9800
C2—C23	1.555 (5)	C23—H23C	0.9800
C3—C32	1.525 (4)	C31—H31A	0.9800
C3—C31	1.546 (4)	C31—H31B	0.9800
C3—H3	1.0000	C31—H31C	0.9800
C11—H11A	0.9800	C32—H32A	0.9800
C11—H11B	0.9800	C32—H32B	0.9800
C11—H11C	0.9800	C32—H32C	0.9800
C12—H12A	0.9800		
			
S1^i^—Pd1—S1	180.0	C1—C12—H12C	109.5
S1^i^—Pd1—Br1	88.867 (17)	H12A—C12—H12C	109.5
S1—Pd1—Br1	91.133 (17)	H12B—C12—H12C	109.5
S1^i^—Pd1—Br1^i^	91.133 (17)	C1—C13—H13A	109.5
S1—Pd1—Br1^i^	88.867 (17)	C1—C13—H13B	109.5
Br1—Pd1—Br1^i^	180.0	H13A—C13—H13B	109.5
P1—S1—Pd1	117.53 (4)	C1—C13—H13C	109.5
C3—P1—C2	106.59 (16)	H13A—C13—H13C	109.5
C3—P1—C1	111.60 (13)	H13B—C13—H13C	109.5
C2—P1—C1	114.28 (18)	C2—C21—H21A	109.5
C3—P1—S1	111.18 (10)	C2—C21—H21B	109.5
C2—P1—S1	111.32 (11)	H21A—C21—H21B	109.5
C1—P1—S1	101.95 (11)	C2—C21—H21C	109.5
C12—C1—C13	109.6 (3)	H21A—C21—H21C	109.5
C12—C1—C11	108.6 (3)	H21B—C21—H21C	109.5
C13—C1—C11	107.6 (3)	C2—C22—H22A	109.5
C12—C1—P1	111.2 (2)	C2—C22—H22B	109.5
C13—C1—P1	109.7 (2)	H22A—C22—H22B	109.5
C11—C1—P1	110.0 (2)	C2—C22—H22C	109.5
C22—C2—C21	108.0 (3)	H22A—C22—H22C	109.5
C22—C2—C23	110.7 (4)	H22B—C22—H22C	109.5
C21—C2—C23	106.9 (3)	C2—C23—H23A	109.5
C22—C2—P1	108.4 (3)	C2—C23—H23B	109.5
C21—C2—P1	110.3 (3)	H23A—C23—H23B	109.5
C23—C2—P1	112.5 (3)	C2—C23—H23C	109.5
C32—C3—C31	109.7 (2)	H23A—C23—H23C	109.5
C32—C3—P1	112.16 (19)	H23B—C23—H23C	109.5
C31—C3—P1	118.4 (2)	C3—C31—H31A	109.5
C32—C3—H3	105.1	C3—C31—H31B	109.5
C31—C3—H3	105.1	H31A—C31—H31B	109.5
P1—C3—H3	105.1	C3—C31—H31C	109.5
C1—C11—H11A	109.5	H31A—C31—H31C	109.5
C1—C11—H11B	109.5	H31B—C31—H31C	109.5
H11A—C11—H11B	109.5	C3—C32—H32A	109.5
C1—C11—H11C	109.5	C3—C32—H32B	109.5
H11A—C11—H11C	109.5	H32A—C32—H32B	109.5
H11B—C11—H11C	109.5	C3—C32—H32C	109.5
C1—C12—H12A	109.5	H32A—C32—H32C	109.5
C1—C12—H12B	109.5	H32B—C32—H32C	109.5
H12A—C12—H12B	109.5		
			
Br1—Pd1—S1—P1	67.56 (4)	C1—P1—C2—C22	167.0 (2)
Br1^i^—Pd1—S1—P1	−112.44 (4)	S1—P1—C2—C22	−78.1 (3)
Pd1—S1—P1—C3	−72.48 (10)	C3—P1—C2—C21	161.3 (3)
Pd1—S1—P1—C2	46.20 (16)	C1—P1—C2—C21	−74.9 (3)
Pd1—S1—P1—C1	168.46 (11)	S1—P1—C2—C21	39.9 (3)
C3—P1—C1—C12	43.8 (3)	C3—P1—C2—C23	−79.4 (3)
C2—P1—C1—C12	−77.2 (3)	C1—P1—C2—C23	44.3 (4)
S1—P1—C1—C12	162.6 (2)	S1—P1—C2—C23	159.2 (3)
C3—P1—C1—C13	165.2 (2)	C2—P1—C3—C32	179.9 (2)
C2—P1—C1—C13	44.2 (3)	C1—P1—C3—C32	54.5 (3)
S1—P1—C1—C13	−76.0 (3)	S1—P1—C3—C32	−58.6 (2)
C3—P1—C1—C11	−76.6 (2)	C2—P1—C3—C31	50.6 (3)
C2—P1—C1—C11	162.3 (2)	C1—P1—C3—C31	−74.8 (3)
S1—P1—C1—C11	42.1 (2)	S1—P1—C3—C31	172.08 (19)
C3—P1—C2—C22	43.3 (3)		

Symmetry code: (i) −*x*+1, −*y*+1, −*z*+1.

### Dibromido(di-tert-butylisopropylphosphine sulfide-κS)palladium(II) (4) Hydrogen-bond geometry (Å, º)

**Table:**

*D*—H···*A*	*D*—H	H···*A*	*D*···*A*	*D*—H···*A*
C3—H3···Br1	1.00	2.81	3.638 (3)	140
C11—H11*C*···S1	0.98	2.63	3.132 (3)	112
C21—H21*C*···S1	0.98	2.85	3.366 (4)	114
C32—H32*C*···S1	0.98	3.02	3.567 (3)	117
C22—H22*C*···Pd1	0.98	2.87	3.778 (4)	154
C12—H12*A*···S1^ii^	0.98	2.95	3.443 (3)	112
C21—H21*C*···Br1^i^	0.98	2.82	3.782 (4)	168

Symmetry codes: (i) −*x*+1, −*y*+1, −*z*+1; (ii) *x*+1/2, −*y*+3/2, *z*+1/2.

### Dichlorido(di-tert-butylisopropylphosphine sulfide-κS)palladium(II) (5) Crystal data

**Table:**

[PdCl_2_(C_11_H_25_PS)_2_]	*F*(000) = 1424
*M**_r_* = 706.67	*D*_x_ = 1.612 Mg m^−^^3^
Monoclinic, *P*2_1_/*c*	Mo *K*α radiation, λ = 0.71073 Å
*a* = 14.5283 (3) Å	Cell parameters from 16523 reflections
*b* = 14.4191 (3) Å	θ = 2.4–30.8°
*c* = 13.9428 (4) Å	µ = 5.27 mm^−^^1^
β = 94.571 (3)°	*T* = 100 K
*V* = 2911.53 (12) Å^3^	Plate, orange
*Z* = 4	0.2 × 0.1 × 0.07 mm

### Dichlorido(di-tert-butylisopropylphosphine sulfide-κS)palladium(II) (5) Data collection

**Table:**

Oxford Diffraction Xcalibur, Eos diffractometer	8721 independent reflections
Radiation source: Enhance (Mo) X-ray Source	6105 reflections with *I* > 2σ(*I*)
Graphite monochromator	*R*_int_ = 0.044
Detector resolution: 16.1419 pixels mm^-1^	θ_max_ = 30.9°, θ_min_ = 2.4°
ω scans	*h* = −20→19
Absorption correction: multi-scan (CrysAlisPro; Rigaku OD, 2012)	*k* = −20→20
*T*_min_ = 0.650, *T*_max_ = 1.000	*l* = −19→19
79369 measured reflections	

### Dichlorido(di-tert-butylisopropylphosphine sulfide-κS)palladium(II) (5) Refinement

**Table:**

Refinement on *F*^2^	Primary atom site location: structure-invariant direct methods
Least-squares matrix: full	Secondary atom site location: difference Fourier map
*R*[*F*^2^ > 2σ(*F*^2^)] = 0.026	Hydrogen site location: inferred from neighbouring sites
*wR*(*F*^2^) = 0.055	H-atom parameters constrained
*S* = 1.04	*w* = 1/[σ^2^(*F*_o_^2^) + (0.0154*P*)^2^ + 5.0893*P*] where *P* = (*F*_o_^2^ + 2*F*_c_^2^)/3
8721 reflections	(Δ/σ)_max_ = 0.001
281 parameters	Δρ_max_ = 1.14 e Å^−^^3^
0 restraints	Δρ_min_ = −0.97 e Å^−^^3^

### Dichlorido(di-tert-butylisopropylphosphine sulfide-κS)palladium(II) (5) Fractional atomic coordinates and isotropic or equivalent isotropic displacement parameters (Å^2^)

**Table:**

	*x*	*y*	*z*	*U*_iso_*/*U*_eq_	
Pt1	0.500000	0.500000	0.500000	0.01098 (4)	
Pt2	1.000000	0.000000	0.500000	0.01384 (4)	
Cl1	0.63902 (5)	0.51548 (5)	0.43147 (5)	0.01956 (14)	
Cl2	1.11830 (6)	−0.09779 (5)	0.46453 (7)	0.0331 (2)	
S1	0.46640 (5)	0.65107 (5)	0.44755 (5)	0.01454 (13)	
S2	1.10486 (5)	0.11932 (5)	0.53673 (5)	0.02066 (15)	
P1	0.36073 (5)	0.65824 (5)	0.34317 (5)	0.01255 (13)	
P2	1.15656 (5)	0.17568 (5)	0.41869 (5)	0.01434 (14)	
C1	0.3559 (2)	0.78408 (19)	0.3069 (2)	0.0168 (6)	
C2	0.3820 (2)	0.5802 (2)	0.2395 (2)	0.0210 (6)	
C3	0.2543 (2)	0.6187 (2)	0.3944 (2)	0.0213 (6)	
H3	0.268369	0.554165	0.417452	0.026*	
C11	0.4342 (2)	0.8074 (2)	0.2434 (2)	0.0241 (7)	
H11A	0.434372	0.874186	0.230651	0.036*	
H11B	0.424701	0.773586	0.182419	0.036*	
H11C	0.493434	0.789167	0.276544	0.036*	
C12	0.3691 (2)	0.8452 (2)	0.3974 (2)	0.0259 (7)	
H12A	0.429443	0.832118	0.431296	0.039*	
H12B	0.320267	0.831639	0.439964	0.039*	
H12C	0.366055	0.910682	0.378725	0.039*	
C13	0.2617 (2)	0.8088 (2)	0.2546 (2)	0.0249 (7)	
H13A	0.264392	0.871411	0.227656	0.037*	
H13B	0.213872	0.806424	0.300297	0.037*	
H13C	0.246881	0.764237	0.202563	0.037*	
C21	0.3226 (3)	0.6059 (2)	0.1473 (2)	0.0356 (9)	
H21A	0.338772	0.668409	0.126848	0.053*	
H21B	0.257188	0.604125	0.159742	0.053*	
H21C	0.333941	0.561417	0.096345	0.053*	
C22	0.3590 (3)	0.4801 (2)	0.2688 (2)	0.0285 (8)	
H22A	0.376003	0.436967	0.218822	0.043*	
H22B	0.292668	0.475023	0.276190	0.043*	
H22C	0.393688	0.464781	0.329878	0.043*	
C23	0.4838 (2)	0.5823 (2)	0.2187 (2)	0.0293 (8)	
H23A	0.494595	0.536750	0.168649	0.044*	
H23B	0.522372	0.567268	0.277471	0.044*	
H23C	0.499647	0.644401	0.196502	0.044*	
C31	0.1662 (2)	0.6095 (3)	0.3268 (3)	0.0405 (10)	
H31A	0.117957	0.579561	0.361093	0.061*	
H31B	0.179136	0.571937	0.270916	0.061*	
H31C	0.145316	0.671282	0.305321	0.061*	
C32	0.2342 (2)	0.6725 (2)	0.4846 (2)	0.0312 (8)	
H32A	0.205901	0.732244	0.465998	0.047*	
H32B	0.291972	0.683219	0.524245	0.047*	
H32C	0.191773	0.636716	0.521383	0.047*	
C4	1.28268 (19)	0.1938 (2)	0.4566 (2)	0.0179 (6)	
C5	1.1497 (2)	0.0919 (2)	0.3175 (2)	0.0208 (6)	
H6	1.171530	0.031438	0.346156	0.025*	
C6	1.0935 (2)	0.2867 (2)	0.3861 (2)	0.0211 (6)	
C41	1.3291 (2)	0.0980 (2)	0.4566 (2)	0.0260 (7)	
H41A	1.294399	0.054273	0.493665	0.039*	
H41B	1.392548	0.102896	0.485921	0.039*	
H41C	1.330114	0.075718	0.390301	0.039*	
C42	1.2955 (2)	0.2306 (2)	0.5602 (2)	0.0249 (7)	
H42A	1.266935	0.292009	0.563232	0.037*	
H42B	1.361545	0.235329	0.580115	0.037*	
H42C	1.266161	0.188009	0.603242	0.037*	
C43	1.3306 (2)	0.2614 (2)	0.3912 (2)	0.0218 (6)	
H43A	1.397265	0.261236	0.408971	0.033*	
H43B	1.306219	0.324094	0.399079	0.033*	
H43C	1.318924	0.242032	0.323990	0.033*	
C51	1.0518 (2)	0.0737 (2)	0.2732 (2)	0.0285 (7)	
H51A	1.051247	0.017243	0.234046	0.043*	
H51B	1.031131	0.126376	0.232513	0.043*	
H51C	1.010113	0.065921	0.324517	0.043*	
C52	1.2114 (2)	0.1109 (2)	0.2353 (2)	0.0292 (7)	
H52A	1.193601	0.170001	0.204498	0.044*	
H52B	1.203986	0.060810	0.187854	0.044*	
H52C	1.276058	0.114029	0.261225	0.044*	
C61	1.1185 (2)	0.3602 (2)	0.4628 (2)	0.0289 (7)	
H61A	1.107728	0.335411	0.526414	0.043*	
H61B	1.079998	0.415351	0.449882	0.043*	
H61C	1.183701	0.377119	0.461402	0.043*	
C62	1.1152 (2)	0.3226 (2)	0.2865 (2)	0.0290 (7)	
H62A	1.182092	0.329578	0.284636	0.043*	
H62B	1.085109	0.382733	0.274379	0.043*	
H62C	1.092081	0.278224	0.237003	0.043*	
C63	0.9891 (2)	0.2686 (2)	0.3848 (3)	0.0300 (8)	
H63A	0.955485	0.326305	0.369323	0.045*	
H63B	0.974242	0.246472	0.448200	0.045*	
H63C	0.971147	0.221626	0.336124	0.045*	

### Dichlorido(di-tert-butylisopropylphosphine sulfide-κS)palladium(II) (5) Atomic displacement parameters (Å^2^)

**Table:**

	*U*^11^	*U*^22^	*U*^33^	*U*^12^	*U*^13^	*U*^23^
Pt1	0.00922 (7)	0.01024 (7)	0.01332 (7)	−0.00095 (5)	−0.00001 (5)	0.00139 (5)
Pt2	0.01099 (7)	0.01107 (7)	0.02000 (8)	−0.00090 (6)	0.00461 (5)	0.00118 (6)
Cl1	0.0139 (3)	0.0204 (3)	0.0250 (4)	0.0013 (3)	0.0052 (3)	0.0077 (3)
Cl2	0.0249 (4)	0.0179 (4)	0.0592 (6)	0.0054 (3)	0.0209 (4)	0.0058 (4)
S1	0.0135 (3)	0.0108 (3)	0.0187 (3)	−0.0019 (2)	−0.0024 (3)	0.0010 (3)
S2	0.0246 (4)	0.0211 (4)	0.0165 (4)	−0.0097 (3)	0.0029 (3)	0.0013 (3)
P1	0.0142 (3)	0.0103 (3)	0.0129 (3)	−0.0006 (3)	−0.0003 (3)	0.0010 (3)
P2	0.0141 (3)	0.0145 (3)	0.0144 (3)	−0.0025 (3)	0.0010 (3)	0.0012 (3)
C1	0.0220 (15)	0.0108 (12)	0.0178 (14)	0.0014 (11)	0.0019 (11)	0.0013 (10)
C2	0.0367 (18)	0.0136 (13)	0.0118 (14)	0.0033 (12)	−0.0031 (12)	−0.0010 (11)
C3	0.0146 (14)	0.0235 (15)	0.0255 (16)	−0.0009 (12)	−0.0011 (12)	0.0105 (12)
C11	0.0313 (17)	0.0137 (13)	0.0280 (17)	0.0000 (12)	0.0070 (14)	0.0079 (12)
C12	0.0398 (19)	0.0119 (13)	0.0254 (16)	0.0015 (13)	−0.0001 (14)	−0.0030 (12)
C13	0.0270 (17)	0.0228 (15)	0.0249 (16)	0.0072 (13)	0.0021 (13)	0.0068 (13)
C21	0.062 (3)	0.0232 (17)	0.0197 (17)	0.0024 (17)	−0.0091 (16)	−0.0039 (13)
C22	0.047 (2)	0.0134 (14)	0.0229 (16)	−0.0028 (13)	−0.0073 (15)	−0.0033 (12)
C23	0.047 (2)	0.0210 (16)	0.0223 (16)	0.0117 (15)	0.0161 (15)	0.0023 (13)
C31	0.0197 (17)	0.048 (2)	0.051 (2)	−0.0112 (16)	−0.0102 (16)	0.0242 (19)
C32	0.0258 (17)	0.0306 (18)	0.039 (2)	0.0119 (14)	0.0170 (15)	0.0122 (15)
C4	0.0146 (14)	0.0179 (13)	0.0210 (15)	−0.0035 (11)	0.0007 (11)	0.0037 (11)
C5	0.0227 (15)	0.0208 (14)	0.0189 (15)	−0.0034 (12)	0.0016 (12)	−0.0017 (12)
C6	0.0209 (15)	0.0171 (14)	0.0253 (16)	0.0016 (12)	0.0013 (12)	0.0041 (12)
C41	0.0201 (16)	0.0212 (15)	0.0363 (19)	0.0014 (12)	−0.0003 (13)	0.0049 (13)
C42	0.0234 (16)	0.0271 (16)	0.0235 (16)	−0.0078 (13)	−0.0021 (13)	0.0007 (13)
C43	0.0180 (15)	0.0239 (15)	0.0239 (16)	−0.0060 (12)	0.0037 (12)	0.0012 (12)
C51	0.0283 (18)	0.0338 (18)	0.0226 (17)	−0.0085 (14)	−0.0027 (13)	−0.0063 (14)
C52	0.0323 (18)	0.0345 (18)	0.0218 (17)	−0.0049 (15)	0.0077 (14)	−0.0071 (14)
C61	0.0359 (19)	0.0190 (15)	0.0321 (18)	0.0069 (14)	0.0042 (15)	−0.0009 (13)
C62	0.0306 (18)	0.0266 (17)	0.0296 (18)	0.0050 (14)	0.0019 (14)	0.0124 (14)
C63	0.0187 (16)	0.0340 (19)	0.037 (2)	0.0052 (14)	0.0026 (14)	0.0068 (15)

### Dichlorido(di-tert-butylisopropylphosphine sulfide-κS)palladium(II) (5) Geometric parameters (Å, º)

**Table:**

Pt1—Cl1	2.3129 (7)	C23—H23B	0.9800
Pt1—Cl1^i^	2.3129 (7)	C23—H23C	0.9800
Pt1—S1	2.3369 (7)	C31—H31A	0.9800
Pt1—S1^i^	2.3369 (7)	C31—H31B	0.9800
Pt2—Cl2	2.3070 (7)	C31—H31C	0.9800
Pt2—Cl2^ii^	2.3070 (7)	C32—H32A	0.9800
Pt2—S2^ii^	2.3278 (7)	C32—H32B	0.9800
Pt2—S2	2.3278 (7)	C32—H32C	0.9800
S1—P1	2.0322 (10)	C4—C42	1.536 (4)
S2—P2	2.0323 (10)	C4—C41	1.537 (4)
P1—C3	1.844 (3)	C4—C43	1.539 (4)
P1—C2	1.877 (3)	C5—C51	1.528 (4)
P1—C1	1.883 (3)	C5—C52	1.535 (4)
P2—C5	1.854 (3)	C5—H6	1.0000
P2—C6	1.882 (3)	C6—C61	1.530 (4)
P2—C4	1.884 (3)	C6—C62	1.537 (4)
C1—C11	1.533 (4)	C6—C63	1.538 (4)
C1—C12	1.539 (4)	C41—H41A	0.9800
C1—C13	1.541 (4)	C41—H41B	0.9800
C2—C23	1.530 (5)	C41—H41C	0.9800
C2—C21	1.536 (4)	C42—H42A	0.9800
C2—C22	1.543 (4)	C42—H42B	0.9800
C3—C32	1.526 (4)	C42—H42C	0.9800
C3—C31	1.533 (4)	C43—H43A	0.9800
C3—H3	1.0000	C43—H43B	0.9800
C11—H11A	0.9800	C43—H43C	0.9800
C11—H11B	0.9800	C51—H51A	0.9800
C11—H11C	0.9800	C51—H51B	0.9800
C12—H12A	0.9800	C51—H51C	0.9800
C12—H12B	0.9800	C52—H52A	0.9800
C12—H12C	0.9800	C52—H52B	0.9800
C13—H13A	0.9800	C52—H52C	0.9800
C13—H13B	0.9800	C61—H61A	0.9800
C13—H13C	0.9800	C61—H61B	0.9800
C21—H21A	0.9800	C61—H61C	0.9800
C21—H21B	0.9800	C62—H62A	0.9800
C21—H21C	0.9800	C62—H62B	0.9800
C22—H22A	0.9800	C62—H62C	0.9800
C22—H22B	0.9800	C63—H63A	0.9800
C22—H22C	0.9800	C63—H63B	0.9800
C23—H23A	0.9800	C63—H63C	0.9800
			
Cl1—Pt1—Cl1^i^	180.0	H23A—C23—H23C	109.5
Cl1—Pt1—S1	87.04 (2)	H23B—C23—H23C	109.5
Cl1^i^—Pt1—S1	92.96 (2)	C3—C31—H31A	109.5
Cl1—Pt1—S1^i^	92.96 (2)	C3—C31—H31B	109.5
Cl1^i^—Pt1—S1^i^	87.04 (2)	H31A—C31—H31B	109.5
S1—Pt1—S1^i^	180.0	C3—C31—H31C	109.5
Cl2—Pt2—Cl2^ii^	180.0	H31A—C31—H31C	109.5
Cl2—Pt2—S2^ii^	89.24 (3)	H31B—C31—H31C	109.5
Cl2^ii^—Pt2—S2^ii^	90.76 (3)	C3—C32—H32A	109.5
Cl2—Pt2—S2	90.75 (3)	C3—C32—H32B	109.5
Cl2^ii^—Pt2—S2	89.25 (3)	H32A—C32—H32B	109.5
S2^ii^—Pt2—S2	180.0	C3—C32—H32C	109.5
P1—S1—Pt1^i^	113.49 (3)	H32A—C32—H32C	109.5
P1—S1—Pt1	113.49 (3)	H32B—C32—H32C	109.5
P2—S2—Pt2^ii^	113.27 (4)	C42—C4—C41	106.9 (2)
P2—S2—Pt2	113.27 (4)	C42—C4—C43	108.1 (2)
C3—P1—C2	107.91 (14)	C41—C4—C43	110.5 (2)
C3—P1—C1	112.81 (13)	C42—C4—P2	110.7 (2)
C2—P1—C1	112.08 (13)	C41—C4—P2	107.03 (19)
C3—P1—S1	108.40 (10)	C43—C4—P2	113.4 (2)
C2—P1—S1	111.07 (10)	C51—C5—C52	108.0 (3)
C1—P1—S1	104.54 (10)	C51—C5—P2	114.3 (2)
C5—P2—C6	112.01 (14)	C52—C5—P2	116.9 (2)
C5—P2—C4	107.21 (13)	C51—C5—H6	105.5
C6—P2—C4	113.18 (13)	C52—C5—H6	105.5
C5—P2—S2	110.77 (10)	P2—C5—H6	105.5
C6—P2—S2	109.37 (10)	C61—C6—C62	110.1 (3)
C4—P2—S2	104.01 (10)	C61—C6—C63	107.7 (3)
C11—C1—C12	107.4 (2)	C62—C6—C63	108.6 (3)
C11—C1—C13	110.3 (2)	C61—C6—P2	109.7 (2)
C12—C1—C13	107.7 (2)	C62—C6—P2	112.0 (2)
C11—C1—P1	110.65 (19)	C63—C6—P2	108.6 (2)
C12—C1—P1	109.38 (19)	C4—C41—H41A	109.5
C13—C1—P1	111.2 (2)	C4—C41—H41B	109.5
C23—C2—C21	108.9 (3)	H41A—C41—H41B	109.5
C23—C2—C22	107.6 (3)	C4—C41—H41C	109.5
C21—C2—C22	109.1 (3)	H41A—C41—H41C	109.5
C23—C2—P1	110.8 (2)	H41B—C41—H41C	109.5
C21—C2—P1	112.6 (2)	C4—C42—H42A	109.5
C22—C2—P1	107.7 (2)	C4—C42—H42B	109.5
C32—C3—C31	110.2 (3)	H42A—C42—H42B	109.5
C32—C3—P1	112.6 (2)	C4—C42—H42C	109.5
C31—C3—P1	118.2 (2)	H42A—C42—H42C	109.5
C32—C3—H3	104.9	H42B—C42—H42C	109.5
C31—C3—H3	104.9	C4—C43—H43A	109.5
P1—C3—H3	104.9	C4—C43—H43B	109.5
C1—C11—H11A	109.5	H43A—C43—H43B	109.5
C1—C11—H11B	109.5	C4—C43—H43C	109.5
H11A—C11—H11B	109.5	H43A—C43—H43C	109.5
C1—C11—H11C	109.5	H43B—C43—H43C	109.5
H11A—C11—H11C	109.5	C5—C51—H51A	109.5
H11B—C11—H11C	109.5	C5—C51—H51B	109.5
C1—C12—H12A	109.5	H51A—C51—H51B	109.5
C1—C12—H12B	109.5	C5—C51—H51C	109.5
H12A—C12—H12B	109.5	H51A—C51—H51C	109.5
C1—C12—H12C	109.5	H51B—C51—H51C	109.5
H12A—C12—H12C	109.5	C5—C52—H52A	109.5
H12B—C12—H12C	109.5	C5—C52—H52B	109.5
C1—C13—H13A	109.5	H52A—C52—H52B	109.5
C1—C13—H13B	109.5	C5—C52—H52C	109.5
H13A—C13—H13B	109.5	H52A—C52—H52C	109.5
C1—C13—H13C	109.5	H52B—C52—H52C	109.5
H13A—C13—H13C	109.5	C6—C61—H61A	109.5
H13B—C13—H13C	109.5	C6—C61—H61B	109.5
C2—C21—H21A	109.5	H61A—C61—H61B	109.5
C2—C21—H21B	109.5	C6—C61—H61C	109.5
H21A—C21—H21B	109.5	H61A—C61—H61C	109.5
C2—C21—H21C	109.5	H61B—C61—H61C	109.5
H21A—C21—H21C	109.5	C6—C62—H62A	109.5
H21B—C21—H21C	109.5	C6—C62—H62B	109.5
C2—C22—H22A	109.5	H62A—C62—H62B	109.5
C2—C22—H22B	109.5	C6—C62—H62C	109.5
H22A—C22—H22B	109.5	H62A—C62—H62C	109.5
C2—C22—H22C	109.5	H62B—C62—H62C	109.5
H22A—C22—H22C	109.5	C6—C63—H63A	109.5
H22B—C22—H22C	109.5	C6—C63—H63B	109.5
C2—C23—H23A	109.5	H63A—C63—H63B	109.5
C2—C23—H23B	109.5	C6—C63—H63C	109.5
H23A—C23—H23B	109.5	H63A—C63—H63C	109.5
C2—C23—H23C	109.5	H63B—C63—H63C	109.5
			
Cl1—Pt1—S1—P1	112.78 (4)	C1—P1—C2—C22	−165.1 (2)
Cl1^i^—Pt1—S1—P1	−67.22 (4)	S1—P1—C2—C22	78.4 (2)
Cl2—Pt2—S2—P2	73.29 (5)	C2—P1—C3—C32	175.4 (2)
Cl2^ii^—Pt2—S2—P2	−106.71 (5)	C1—P1—C3—C32	−60.2 (2)
Pt1^i^—S1—P1—C3	64.50 (11)	S1—P1—C3—C32	55.1 (2)
Pt1—S1—P1—C3	64.50 (11)	C2—P1—C3—C31	−54.3 (3)
Pt1^i^—S1—P1—C2	−53.88 (11)	C1—P1—C3—C31	70.1 (3)
Pt1—S1—P1—C2	−53.88 (11)	S1—P1—C3—C31	−174.7 (2)
Pt1^i^—S1—P1—C1	−174.95 (9)	C5—P2—C4—C42	−158.5 (2)
Pt1—S1—P1—C1	−174.95 (9)	C6—P2—C4—C42	77.5 (2)
Pt2^ii^—S2—P2—C5	−24.15 (12)	S2—P2—C4—C42	−41.1 (2)
Pt2—S2—P2—C5	−24.15 (12)	C5—P2—C4—C41	−42.4 (2)
Pt2^ii^—S2—P2—C6	99.77 (11)	C6—P2—C4—C41	−166.4 (2)
Pt2—S2—P2—C6	99.77 (11)	S2—P2—C4—C41	75.0 (2)
Pt2^ii^—S2—P2—C4	−139.04 (10)	C5—P2—C4—C43	79.8 (2)
Pt2—S2—P2—C4	−139.04 (10)	C6—P2—C4—C43	−44.2 (3)
C3—P1—C1—C11	−166.3 (2)	S2—P2—C4—C43	−162.85 (19)
C2—P1—C1—C11	−44.2 (3)	C6—P2—C5—C51	−51.5 (3)
S1—P1—C1—C11	76.2 (2)	C4—P2—C5—C51	−176.3 (2)
C3—P1—C1—C12	75.6 (2)	S2—P2—C5—C51	70.9 (2)
C2—P1—C1—C12	−162.3 (2)	C6—P2—C5—C52	76.0 (3)
S1—P1—C1—C12	−41.9 (2)	C4—P2—C5—C52	−48.7 (3)
C3—P1—C1—C13	−43.2 (2)	S2—P2—C5—C52	−161.6 (2)
C2—P1—C1—C13	78.8 (2)	C5—P2—C6—C61	−167.8 (2)
S1—P1—C1—C13	−160.78 (18)	C4—P2—C6—C61	−46.5 (3)
C3—P1—C2—C23	−157.8 (2)	S2—P2—C6—C61	69.0 (2)
C1—P1—C2—C23	77.4 (2)	C5—P2—C6—C62	−45.2 (3)
S1—P1—C2—C23	−39.1 (2)	C4—P2—C6—C62	76.1 (3)
C3—P1—C2—C21	80.1 (3)	S2—P2—C6—C62	−168.44 (19)
C1—P1—C2—C21	−44.7 (3)	C5—P2—C6—C63	74.7 (2)
S1—P1—C2—C21	−161.3 (2)	C4—P2—C6—C63	−164.0 (2)
C3—P1—C2—C22	−40.3 (2)	S2—P2—C6—C63	−48.5 (2)

Symmetry codes: (i) −*x*+1, −*y*+1, −*z*+1; (ii) −*x*+2, −*y*, −*z*+1.

### Dichlorido(di-tert-butylisopropylphosphine sulfide-κS)palladium(II) (5) Hydrogen-bond geometry (Å, º)

**Table:**

*D*—H···*A*	*D*—H	H···*A*	*D*···*A*	*D*—H···*A*
C3—H3···Cl1^i^	1.00	2.61	3.382 (3)	134
C5—H6···Cl2	1.00	2.65	3.470 (3)	140
C41—H41*A*···S2	0.98	3.01	3.541 (3)	115
C42—H42*C*···S2	0.98	2.64	3.195 (3)	116
C63—H63*B*···Cl2^ii^	0.98	2.85	3.666 (3)	141
C63—H63*B*···S2	0.98	2.85	3.374 (3)	114
C12—H12*A*···S1	0.98	2.67	3.188 (3)	113
C23—H23*B*···Cl1	0.98	2.73	3.708 (4)	175
C23—H23*B*···S1	0.98	2.84	3.370 (3)	115
C32—H32*B*···S1	0.98	2.87	3.467 (3)	121
C22—H22*C*···Pt1^i^	0.98	2.77	3.691 (3)	156
C51—H51*C*···Pt2^ii^	0.98	2.64	3.475 (3)	143
C32—H32*A*···Cl2^iii^	0.98	2.76	3.716 (3)	165
C43—H43*A*···S1^iv^	0.98	2.98	3.779 (3)	139

Symmetry codes: (i) −*x*+1, −*y*+1, −*z*+1; (ii) −*x*+2, −*y*, −*z*+1; (iii) *x*−1, *y*+1, *z*; (iv) −*x*+2, −*y*+1, −*z*+1.

### Di-µ-chlorido-bis[(tert-butyldiisopropylphosphine sulfide-κS)chloridopalladium(II)] (6) Crystal data

**Table:**

[PdCl_2_(C_10_H_23_PS)_2_]	*Z* = 1
*M**_r_* = 767.23	*F*(000) = 388
Triclinic, *P*1	*D*_x_ = 1.653 Mg m^−^^3^
*a* = 6.9753 (4) Å	Mo *K*α radiation, λ = 0.71073 Å
*b* = 8.7642 (5) Å	Cell parameters from 13225 reflections
*c* = 13.0718 (7) Å	θ = 2.4–30.8°
α = 88.930 (6)°	µ = 1.76 mm^−^^1^
β = 78.488 (6)°	*T* = 100 K
γ = 79.804 (7)°	Plate, dichroic yellow orange
*V* = 770.56 (8) Å^3^	0.17 × 0.06 × 0.02 mm

### Di-µ-chlorido-bis[(tert-butyldiisopropylphosphine sulfide-κS)chloridopalladium(II)] (6) Data collection

**Table:**

Oxford Diffraction Xcalibur, Eos diffractometer	4550 independent reflections
Radiation source: fine-focus sealed tube	3905 reflections with *I* > 2σ(*I*)
Graphite monochromator	*R*_int_ = 0.052
Detector resolution: 16.1419 pixels mm^-1^	θ_max_ = 30.8°, θ_min_ = 2.4°
ω scans	*h* = −9→10
Absorption correction: multi-scan (CrysAlisPro; Rigaku OD, 2012)	*k* = −12→12
*T*_min_ = 0.754, *T*_max_ = 0.966	*l* = −18→18
48215 measured reflections	

### Di-µ-chlorido-bis[(tert-butyldiisopropylphosphine sulfide-κS)chloridopalladium(II)] (6) Refinement

**Table:**

Refinement on *F*^2^	Primary atom site location: structure-invariant direct methods
Least-squares matrix: full	Secondary atom site location: difference Fourier map
*R*[*F*^2^ > 2σ(*F*^2^)] = 0.029	Hydrogen site location: inferred from neighbouring sites
*wR*(*F*^2^) = 0.059	H-atom parameters constrained
*S* = 1.05	*w* = 1/[σ^2^(*F*_o_^2^) + (0.022*P*)^2^ + 0.6436*P*] where *P* = (*F*_o_^2^ + 2*F*_c_^2^)/3
4550 reflections	(Δ/σ)_max_ = 0.001
143 parameters	Δρ_max_ = 1.29 e Å^−^^3^
0 restraints	Δρ_min_ = −0.58 e Å^−^^3^

### Di-µ-chlorido-bis[(tert-butyldiisopropylphosphine sulfide-κS)chloridopalladium(II)] (6) Special details

**Table:**

Geometry. All esds (except the esd in the dihedral angle between two l.s. planes) are estimated using the full covariance matrix. The cell esds are taken into account individually in the estimation of esds in distances, angles and torsion angles; correlations between esds in cell parameters are only used when they are defined by crystal symmetry. An approximate (isotropic) treatment of cell esds is used for estimating esds involving l.s. planes.

### Di-µ-chlorido-bis[(tert-butyldiisopropylphosphine sulfide-κS)chloridopalladium(II)] (6) Fractional atomic coordinates and isotropic or equivalent isotropic displacement parameters (Å^2^)

**Table:**

	*x*	*y*	*z*	*U*_iso_*/*U*_eq_	
Pd1	0.51394 (2)	0.06683 (2)	0.37430 (2)	0.01336 (5)	
Cl1	0.65368 (8)	−0.04335 (6)	0.21405 (4)	0.02082 (12)	
Cl2	0.39202 (8)	0.16888 (6)	0.54318 (4)	0.01794 (11)	
S1	0.42186 (8)	0.30672 (6)	0.30898 (4)	0.01541 (11)	
P1	0.18827 (8)	0.29587 (6)	0.23938 (4)	0.01113 (10)	
C1	0.0763 (3)	0.5020 (2)	0.22052 (17)	0.0176 (4)	
C2	−0.0026 (3)	0.1987 (2)	0.31853 (16)	0.0139 (4)	
H2	−0.117975	0.212802	0.282397	0.017*	
C3	0.2721 (3)	0.1824 (3)	0.11670 (16)	0.0165 (4)	
H3	0.319466	0.074571	0.138464	0.020*	
C11	0.1990 (4)	0.5677 (3)	0.12441 (19)	0.0261 (5)	
H11A	0.150739	0.679261	0.120726	0.039*	
H11B	0.185254	0.515820	0.061147	0.039*	
H11C	0.339152	0.549753	0.130053	0.039*	
C12	0.0766 (4)	0.6024 (3)	0.31521 (19)	0.0246 (5)	
H12A	0.213851	0.608368	0.319203	0.037*	
H12B	0.012994	0.556072	0.379020	0.037*	
H12C	0.003033	0.706862	0.307981	0.037*	
C13	−0.1395 (3)	0.5133 (3)	0.2055 (2)	0.0241 (5)	
H13A	−0.220896	0.480355	0.269184	0.036*	
H13B	−0.141633	0.446013	0.146814	0.036*	
H13C	−0.192987	0.620778	0.190777	0.036*	
C21	−0.0800 (3)	0.2700 (3)	0.42818 (17)	0.0210 (5)	
H21A	−0.172218	0.208534	0.468672	0.032*	
H21B	−0.149281	0.376538	0.423102	0.032*	
H21C	0.031967	0.270646	0.462986	0.032*	
C22	0.0679 (3)	0.0233 (3)	0.32575 (19)	0.0204 (5)	
H22A	0.180471	0.004640	0.361567	0.031*	
H22B	0.109462	−0.022640	0.255299	0.031*	
H22C	−0.041055	−0.023900	0.364992	0.031*	
C31	0.4524 (4)	0.2273 (3)	0.04244 (17)	0.0232 (5)	
H31A	0.517504	0.140119	−0.005206	0.035*	
H31B	0.546491	0.253151	0.083088	0.035*	
H31C	0.408421	0.317434	0.002014	0.035*	
C32	0.1047 (4)	0.1677 (3)	0.05945 (18)	0.0245 (5)	
H32A	0.058487	0.268083	0.030222	0.037*	
H32B	−0.005830	0.135317	0.108573	0.037*	
H32C	0.154396	0.090228	0.002903	0.037*	

### Di-µ-chlorido-bis[(tert-butyldiisopropylphosphine sulfide-κS)chloridopalladium(II)] (6) Atomic displacement parameters (Å^2^)

**Table:**

	*U*^11^	*U*^22^	*U*^33^	*U*^12^	*U*^13^	*U*^23^
Pd1	0.01149 (8)	0.01694 (8)	0.01083 (8)	−0.00036 (6)	−0.00233 (6)	0.00143 (6)
Cl1	0.0223 (3)	0.0237 (3)	0.0129 (2)	0.0047 (2)	−0.0027 (2)	−0.0012 (2)
Cl2	0.0195 (3)	0.0189 (2)	0.0127 (2)	0.0027 (2)	−0.00211 (19)	0.00072 (19)
S1	0.0153 (2)	0.0168 (2)	0.0159 (2)	−0.00509 (19)	−0.0054 (2)	0.00214 (19)
P1	0.0129 (2)	0.0110 (2)	0.0095 (2)	−0.00207 (19)	−0.00224 (19)	0.00125 (18)
C1	0.0202 (11)	0.0124 (9)	0.0198 (11)	−0.0021 (8)	−0.0039 (9)	0.0026 (8)
C2	0.0140 (9)	0.0145 (9)	0.0138 (9)	−0.0047 (8)	−0.0027 (8)	0.0035 (7)
C3	0.0194 (10)	0.0164 (10)	0.0126 (9)	−0.0011 (8)	−0.0021 (8)	−0.0016 (8)
C11	0.0326 (13)	0.0190 (11)	0.0254 (12)	−0.0050 (10)	−0.0032 (10)	0.0121 (9)
C12	0.0314 (13)	0.0139 (10)	0.0273 (12)	−0.0015 (9)	−0.0046 (10)	−0.0050 (9)
C13	0.0214 (11)	0.0178 (11)	0.0334 (13)	0.0005 (9)	−0.0101 (10)	0.0029 (10)
C21	0.0189 (11)	0.0273 (12)	0.0162 (10)	−0.0076 (9)	0.0010 (9)	0.0007 (9)
C22	0.0210 (11)	0.0157 (10)	0.0264 (12)	−0.0062 (9)	−0.0076 (9)	0.0070 (9)
C31	0.0250 (12)	0.0272 (12)	0.0140 (10)	−0.0026 (10)	0.0021 (9)	0.0013 (9)
C32	0.0259 (12)	0.0311 (13)	0.0176 (11)	−0.0040 (10)	−0.0073 (9)	−0.0046 (9)

### Di-µ-chlorido-bis[(tert-butyldiisopropylphosphine sulfide-κS)chloridopalladium(II)] (6) Geometric parameters (Å, º)

**Table:**

Pd1—Cl1	2.2799 (6)	C11—H11C	0.9800
Pd1—S1	2.2882 (6)	C12—H12A	0.9800
Pd1—Cl2	2.3349 (5)	C12—H12B	0.9800
Pd1—Cl2^i^	2.3623 (6)	C12—H12C	0.9800
S1—P1	2.0350 (7)	C13—H13A	0.9800
P1—C3	1.839 (2)	C13—H13B	0.9800
P1—C2	1.841 (2)	C13—H13C	0.9800
P1—C1	1.870 (2)	C21—H21A	0.9800
C1—C12	1.532 (3)	C21—H21B	0.9800
C1—C11	1.536 (3)	C21—H21C	0.9800
C1—C13	1.542 (3)	C22—H22A	0.9800
C2—C21	1.530 (3)	C22—H22B	0.9800
C2—C22	1.536 (3)	C22—H22C	0.9800
C2—H2	1.0000	C31—H31A	0.9800
C3—C32	1.531 (3)	C31—H31B	0.9800
C3—C31	1.534 (3)	C31—H31C	0.9800
C3—H3	1.0000	C32—H32A	0.9800
C11—H11A	0.9800	C32—H32B	0.9800
C11—H11B	0.9800	C32—H32C	0.9800
			
Cl1—Pd1—S1	93.95 (2)	H11B—C11—H11C	109.5
Cl1—Pd1—Cl2	175.53 (2)	C1—C12—H12A	109.5
S1—Pd1—Cl2	89.38 (2)	C1—C12—H12B	109.5
Cl1—Pd1—Cl2^i^	91.03 (2)	H12A—C12—H12B	109.5
S1—Pd1—Cl2^i^	174.586 (19)	C1—C12—H12C	109.5
Cl2—Pd1—Cl2^i^	85.54 (2)	H12A—C12—H12C	109.5
Pd1—Cl2—Pd1^i^	94.46 (2)	H12B—C12—H12C	109.5
P1—S1—Pd1	107.34 (3)	C1—C13—H13A	109.5
C3—P1—C2	105.26 (10)	C1—C13—H13B	109.5
C3—P1—C1	113.34 (10)	H13A—C13—H13B	109.5
C2—P1—C1	108.97 (10)	C1—C13—H13C	109.5
C3—P1—S1	110.54 (7)	H13A—C13—H13C	109.5
C2—P1—S1	113.53 (7)	H13B—C13—H13C	109.5
C1—P1—S1	105.38 (8)	C2—C21—H21A	109.5
C12—C1—C11	107.57 (19)	C2—C21—H21B	109.5
C12—C1—C13	108.94 (19)	H21A—C21—H21B	109.5
C11—C1—C13	109.29 (19)	C2—C21—H21C	109.5
C12—C1—P1	110.55 (15)	H21A—C21—H21C	109.5
C11—C1—P1	110.01 (15)	H21B—C21—H21C	109.5
C13—C1—P1	110.41 (15)	C2—C22—H22A	109.5
C21—C2—C22	109.89 (18)	C2—C22—H22B	109.5
C21—C2—P1	112.98 (15)	H22A—C22—H22B	109.5
C22—C2—P1	112.56 (15)	C2—C22—H22C	109.5
C21—C2—H2	107.0	H22A—C22—H22C	109.5
C22—C2—H2	107.0	H22B—C22—H22C	109.5
P1—C2—H2	107.0	C3—C31—H31A	109.5
C32—C3—C31	111.67 (18)	C3—C31—H31B	109.5
C32—C3—P1	113.93 (15)	H31A—C31—H31B	109.5
C31—C3—P1	115.39 (16)	C3—C31—H31C	109.5
C32—C3—H3	104.9	H31A—C31—H31C	109.5
C31—C3—H3	104.9	H31B—C31—H31C	109.5
P1—C3—H3	104.9	C3—C32—H32A	109.5
C1—C11—H11A	109.5	C3—C32—H32B	109.5
C1—C11—H11B	109.5	H32A—C32—H32B	109.5
H11A—C11—H11B	109.5	C3—C32—H32C	109.5
C1—C11—H11C	109.5	H32A—C32—H32C	109.5
H11A—C11—H11C	109.5	H32B—C32—H32C	109.5
			
S1—Pd1—Cl2—Pd1^i^	−178.13 (2)	C2—P1—C1—C13	−37.80 (18)
Cl2^i^—Pd1—Cl2—Pd1^i^	0.0	S1—P1—C1—C13	−159.96 (14)
Cl1—Pd1—S1—P1	73.01 (3)	C3—P1—C2—C21	175.65 (16)
Cl2—Pd1—S1—P1	−109.98 (3)	C1—P1—C2—C21	−62.49 (18)
Pd1—S1—P1—C3	−72.23 (8)	S1—P1—C2—C21	54.62 (17)
Pd1—S1—P1—C2	45.78 (8)	C3—P1—C2—C22	50.45 (18)
Pd1—S1—P1—C1	164.96 (7)	C1—P1—C2—C22	172.32 (15)
C3—P1—C1—C12	−160.34 (16)	S1—P1—C2—C22	−70.58 (16)
C2—P1—C1—C12	82.83 (18)	C2—P1—C3—C32	56.71 (18)
S1—P1—C1—C12	−39.34 (17)	C1—P1—C3—C32	−62.28 (19)
C3—P1—C1—C11	−41.68 (19)	S1—P1—C3—C32	179.67 (14)
C2—P1—C1—C11	−158.51 (16)	C2—P1—C3—C31	−172.15 (16)
S1—P1—C1—C11	79.32 (16)	C1—P1—C3—C31	68.86 (19)
C3—P1—C1—C13	79.03 (18)	S1—P1—C3—C31	−49.19 (18)

Symmetry code: (i) −*x*+1, −*y*, −*z*+1.

### Di-µ-chlorido-bis[(tert-butyldiisopropylphosphine sulfide-κS)chloridopalladium(II)] (6) Hydrogen-bond geometry (Å, º)

**Table:**

*D*—H···*A*	*D*—H	H···*A*	*D*···*A*	*D*—H···*A*
C3—H3···Cl1	1.00	2.74	3.466 (2)	130
C13—H13*C*···Cl1^ii^	0.98	2.95	3.893 (2)	162
C31—H31*B*···S1	0.98	2.93	3.523 (2)	120
C32—H32*C*···Cl1^iii^	0.98	2.89	3.728 (2)	144
C21—H21*C*···Cl2	0.98	2.90	3.844 (2)	161
C22—H22*C*···Cl2^iv^	0.98	2.99	3.960 (2)	172
C22—H22*A*···Pd1	0.98	2.52	3.383 (2)	147
C2—H2···Pd1^v^	1.00	3.09	3.688 (2)	120
C22—H22*C*···Pd1^v^	0.98	3.04	3.740 (2)	129
C21—H21*A*···Pd1^v^	0.98	3.16	3.784 (2)	123

Symmetry codes: (ii) *x*−1, *y*+1, *z*; (iii) −*x*+1, −*y*, −*z*; (iv) −*x*, −*y*, −*z*+1; (v) *x*−1, *y*, *z*.

### (Bromosulfanyl)di-tert-butylisopropylphosphanium di-µ-bromido-bis[dibromidopalladium(II)] (7) Crystal data

**Table:**

(C_11_H_25_BrPS)_2_[Pd_2_Br_6_]	*F*(000) = 1232
*M**_r_* = 1292.76	*D*_x_ = 2.292 Mg m^−^^3^
Monoclinic, *P*2_1_/*c*	Mo *K*α radiation, λ = 0.71073 Å
*a* = 7.8691 (4) Å	Cell parameters from 8375 reflections
*b* = 22.7255 (8) Å	θ = 2.1–29.2°
*c* = 10.5879 (3) Å	µ = 9.70 mm^−^^1^
β = 98.386 (3)°	*T* = 100 K
*V* = 1873.18 (13) Å^3^	Plate, red
*Z* = 2	0.2 × 0.1 × 0.01 mm

### (Bromosulfanyl)di-tert-butylisopropylphosphanium di-µ-bromido-bis[dibromidopalladium(II)] (7) Data collection

**Table:**

Oxford Diffraction Xcalibur, Eos diffractometer	4622 independent reflections
Radiation source: Enhance (Mo) X-ray Source	3740 reflections with *I* > 2σ(*I*)
Graphite monochromator	*R*_int_ = 0.082
Detector resolution: 16.1419 pixels mm^-1^	θ_max_ = 28.3°, θ_min_ = 2.1°
ω scans	*h* = −10→10
Absorption correction: multi-scan (CrysAlisPro; Rigaku OD, 2012)	*k* = −30→30
*T*_min_ = 0.495, *T*_max_ = 1.000	*l* = −14→14
52903 measured reflections	

### (Bromosulfanyl)di-tert-butylisopropylphosphanium di-µ-bromido-bis[dibromidopalladium(II)] (7) Refinement

**Table:**

Refinement on *F*^2^	Primary atom site location: structure-invariant direct methods
Least-squares matrix: full	Secondary atom site location: difference Fourier map
*R*[*F*^2^ > 2σ(*F*^2^)] = 0.042	Hydrogen site location: inferred from neighbouring sites
*wR*(*F*^2^) = 0.072	H-atom parameters constrained
*S* = 1.11	*w* = 1/[σ^2^(*F*_o_^2^) + (0.0177*P*)^2^ + 6.208*P*] where *P* = (*F*_o_^2^ + 2*F*_c_^2^)/3
4622 reflections	(Δ/σ)_max_ = 0.001
196 parameters	Δρ_max_ = 0.71 e Å^−^^3^
87 restraints	Δρ_min_ = −0.95 e Å^−^^3^

### (Bromosulfanyl)di-tert-butylisopropylphosphanium di-µ-bromido-bis[dibromidopalladium(II)] (7) Fractional atomic coordinates and isotropic or equivalent isotropic displacement parameters (Å^2^)

**Table:**

	*x*	*y*	*z*	*U*_iso_*/*U*_eq_	Occ. (<1)
Br1	0.39593 (7)	0.61750 (2)	0.46899 (5)	0.02308 (12)	
P1	0.43599 (16)	0.63574 (5)	0.78836 (12)	0.0152 (3)	
S1	0.57414 (17)	0.64827 (6)	0.63578 (12)	0.0201 (3)	
Pd1	−0.00182 (5)	0.56159 (2)	0.39219 (3)	0.01429 (9)	
Br2	0.14330 (7)	0.58219 (2)	0.21071 (5)	0.02152 (12)	
Br3	0.13664 (6)	0.46495 (2)	0.42182 (5)	0.01861 (12)	
Br4	−0.14636 (7)	0.65550 (2)	0.37175 (5)	0.02252 (12)	
C1	0.6145 (6)	0.6373 (2)	0.9272 (4)	0.0195 (10)	
C11	0.5461 (9)	0.6149 (4)	1.0480 (6)	0.0291 (19)	0.806 (14)
H11A	0.516541	0.573107	1.037742	0.044*	0.806 (14)
H11B	0.443594	0.637366	1.060595	0.044*	0.806 (14)
H11C	0.634802	0.619982	1.122408	0.044*	0.806 (14)
C12	0.6801 (10)	0.7008 (3)	0.9529 (7)	0.030 (2)	0.806 (14)
H12A	0.784684	0.700238	1.015776	0.046*	0.806 (14)
H12B	0.591760	0.724207	0.986083	0.046*	0.806 (14)
H12C	0.705636	0.718364	0.873216	0.046*	0.806 (14)
C13	0.7632 (9)	0.5987 (4)	0.9015 (7)	0.028 (2)	0.806 (14)
H13A	0.849008	0.596782	0.978382	0.042*	0.806 (14)
H13B	0.815668	0.615459	0.831111	0.042*	0.806 (14)
H13C	0.720826	0.559044	0.878454	0.042*	0.806 (14)
C11'	0.749 (3)	0.6836 (11)	0.902 (3)	0.026 (8)*	0.194 (14)
H11D	0.782645	0.676778	0.817698	0.040*	0.194 (14)
H11E	0.699108	0.723087	0.904523	0.040*	0.194 (14)
H11F	0.849914	0.680470	0.967595	0.040*	0.194 (14)
C12'	0.708 (4)	0.5767 (8)	0.931 (3)	0.019 (7)*	0.194 (14)
H12D	0.748162	0.569631	0.848642	0.028*	0.194 (14)
H12E	0.806985	0.576953	0.998990	0.028*	0.194 (14)
H12F	0.628702	0.545347	0.947110	0.028*	0.194 (14)
C13'	0.556 (4)	0.6455 (14)	1.0569 (19)	0.022 (8)*	0.194 (14)
H13D	0.471188	0.615273	1.068942	0.033*	0.194 (14)
H13E	0.655300	0.641940	1.124171	0.033*	0.194 (14)
H13F	0.504494	0.684557	1.061098	0.033*	0.194 (14)
C2	0.3250 (6)	0.5645 (2)	0.7640 (5)	0.0189 (10)	
H2	0.240842	0.569285	0.684305	0.023*	
C21	0.4409 (7)	0.5136 (2)	0.7369 (6)	0.0305 (13)	
H21A	0.518108	0.503466	0.815075	0.046*	
H21B	0.508807	0.525260	0.670503	0.046*	
H21C	0.370096	0.479360	0.707790	0.046*	
C22	0.2171 (9)	0.5480 (3)	0.8679 (6)	0.0408 (16)	
H22A	0.155513	0.511174	0.844575	0.061*	
H22B	0.134199	0.579387	0.876382	0.061*	
H22C	0.292551	0.542783	0.949374	0.061*	
C3	0.2742 (7)	0.6967 (2)	0.7887 (5)	0.0249 (12)	
C31	0.1155 (13)	0.6835 (5)	0.6871 (9)	0.030 (2)	0.80 (3)
H31A	0.029423	0.714453	0.689240	0.045*	0.80 (3)
H31B	0.066127	0.645410	0.705724	0.045*	0.80 (3)
H31C	0.151004	0.682183	0.602136	0.045*	0.80 (3)
C32	0.2141 (15)	0.7059 (6)	0.9180 (7)	0.039 (3)	0.80 (3)
H32A	0.309512	0.720990	0.979274	0.059*	0.80 (3)
H32B	0.174793	0.668312	0.948854	0.059*	0.80 (3)
H32C	0.119269	0.734253	0.908965	0.059*	0.80 (3)
C33	0.3548 (12)	0.7548 (3)	0.7488 (14)	0.044 (3)	0.80 (3)
H33A	0.267982	0.786020	0.740752	0.066*	0.80 (3)
H33B	0.396150	0.749228	0.666732	0.066*	0.80 (3)
H33C	0.451165	0.765887	0.813872	0.066*	0.80 (3)
C31'	0.167 (4)	0.6839 (16)	0.897 (3)	0.020*	0.20 (3)
H31D	0.110910	0.645445	0.882746	0.030*	0.20 (3)
H31E	0.242663	0.683535	0.979350	0.030*	0.20 (3)
H31F	0.079489	0.714485	0.898118	0.030*	0.20 (3)
C32'	0.363 (4)	0.7560 (10)	0.808 (4)	0.029 (10)*	0.20 (3)
H32D	0.441494	0.755791	0.889056	0.044*	0.20 (3)
H32E	0.428090	0.763569	0.737704	0.044*	0.20 (3)
H32F	0.276691	0.786928	0.810399	0.044*	0.20 (3)
C33'	0.148 (5)	0.693 (2)	0.664 (2)	0.022 (10)*	0.20 (3)
H33D	0.094962	0.654067	0.656033	0.033*	0.20 (3)
H33E	0.059080	0.723190	0.663899	0.033*	0.20 (3)
H33F	0.210480	0.699831	0.591204	0.033*	0.20 (3)

### (Bromosulfanyl)di-tert-butylisopropylphosphanium di-µ-bromido-bis[dibromidopalladium(II)] (7) Atomic displacement parameters (Å^2^)

**Table:**

	*U*^11^	*U*^22^	*U*^33^	*U*^12^	*U*^13^	*U*^23^
Br1	0.0307 (3)	0.0269 (3)	0.0112 (2)	−0.0003 (2)	0.0017 (2)	−0.0001 (2)
P1	0.0203 (6)	0.0153 (6)	0.0102 (6)	−0.0004 (5)	0.0028 (5)	0.0005 (5)
S1	0.0269 (7)	0.0211 (7)	0.0129 (6)	−0.0055 (5)	0.0049 (5)	−0.0007 (5)
Pd1	0.01969 (19)	0.01357 (18)	0.00967 (18)	0.00020 (14)	0.00232 (14)	−0.00032 (14)
Br2	0.0287 (3)	0.0237 (3)	0.0133 (2)	−0.0017 (2)	0.0067 (2)	0.0026 (2)
Br3	0.0251 (3)	0.0171 (2)	0.0154 (2)	0.0045 (2)	0.0088 (2)	0.00110 (19)
Br4	0.0297 (3)	0.0163 (3)	0.0210 (3)	0.0049 (2)	0.0020 (2)	0.0014 (2)
C1	0.026 (3)	0.021 (3)	0.011 (2)	−0.006 (2)	0.001 (2)	−0.0004 (19)
C11	0.029 (4)	0.042 (5)	0.015 (3)	−0.009 (4)	−0.001 (3)	0.003 (3)
C12	0.037 (4)	0.028 (4)	0.025 (4)	−0.010 (3)	0.000 (3)	−0.003 (3)
C13	0.022 (4)	0.044 (5)	0.016 (4)	0.005 (3)	−0.003 (3)	−0.004 (3)
C2	0.022 (3)	0.021 (3)	0.013 (2)	−0.006 (2)	−0.001 (2)	0.000 (2)
C21	0.034 (3)	0.016 (3)	0.039 (4)	0.000 (2)	0.000 (3)	0.002 (2)
C22	0.054 (4)	0.041 (4)	0.031 (3)	−0.024 (3)	0.016 (3)	−0.011 (3)
C3	0.028 (3)	0.024 (3)	0.023 (3)	0.010 (2)	0.004 (2)	−0.001 (2)
C31	0.036 (5)	0.032 (5)	0.021 (4)	0.013 (4)	0.004 (4)	−0.007 (4)
C32	0.045 (6)	0.049 (7)	0.023 (4)	0.019 (5)	0.003 (4)	−0.010 (4)
C33	0.071 (6)	0.017 (4)	0.046 (7)	0.007 (3)	0.014 (5)	−0.003 (4)

### (Bromosulfanyl)di-tert-butylisopropylphosphanium di-µ-bromido-bis[dibromidopalladium(II)] (7) Geometric parameters (Å, º)

**Table:**

Br1—S1	2.2027 (14)	C2—C21	1.525 (7)
Br1—Br2	3.2387 (7)	C2—C22	1.531 (7)
P1—C2	1.840 (5)	C2—H2	1.0000
P1—C1	1.879 (5)	C21—H21A	0.9800
P1—C3	1.881 (5)	C21—H21B	0.9800
P1—S1	2.0941 (18)	C21—H21C	0.9800
Pd1—Br4	2.4131 (6)	C22—H22A	0.9800
Pd1—Br2	2.4199 (6)	C22—H22B	0.9800
Pd1—Br3^i^	2.4447 (6)	C22—H22C	0.9800
Pd1—Br3	2.4514 (6)	C3—C32'	1.518 (14)
C1—C13	1.518 (7)	C3—C32	1.527 (8)
C1—C13'	1.522 (14)	C3—C33'	1.536 (14)
C1—C11'	1.542 (14)	C3—C31'	1.549 (14)
C1—C12	1.544 (7)	C3—C33	1.550 (8)
C1—C11	1.545 (7)	C3—C31	1.554 (8)
C1—C12'	1.562 (13)	C31—H31A	0.9800
C11—H11A	0.9800	C31—H31B	0.9800
C11—H11B	0.9800	C31—H31C	0.9800
C11—H11C	0.9800	C32—H32A	0.9800
C12—H12A	0.9800	C32—H32B	0.9800
C12—H12B	0.9800	C32—H32C	0.9800
C12—H12C	0.9800	C33—H33A	0.9800
C13—H13A	0.9800	C33—H33B	0.9800
C13—H13B	0.9800	C33—H33C	0.9800
C13—H13C	0.9800	C31'—H31D	0.9800
C11'—H11D	0.9800	C31'—H31E	0.9800
C11'—H11E	0.9800	C31'—H31F	0.9800
C11'—H11F	0.9800	C32'—H32D	0.9800
C12'—H12D	0.9800	C32'—H32E	0.9800
C12'—H12E	0.9800	C32'—H32F	0.9800
C12'—H12F	0.9800	C33'—H33D	0.9800
C13'—H13D	0.9800	C33'—H33E	0.9800
C13'—H13E	0.9800	C33'—H33F	0.9800
C13'—H13F	0.9800		
			
S1—Br1—Br2	175.04 (4)	C21—C2—P1	114.2 (4)
C2—P1—C1	114.3 (2)	C22—C2—P1	114.6 (4)
C2—P1—C3	109.9 (2)	C21—C2—H2	105.3
C1—P1—C3	114.4 (2)	C22—C2—H2	105.3
C2—P1—S1	107.50 (17)	P1—C2—H2	105.3
C1—P1—S1	100.83 (16)	C2—C21—H21A	109.5
C3—P1—S1	109.19 (17)	C2—C21—H21B	109.5
P1—S1—Br1	103.52 (7)	H21A—C21—H21B	109.5
Br4—Pd1—Br2	91.64 (2)	C2—C21—H21C	109.5
Br4—Pd1—Br3^i^	92.07 (2)	H21A—C21—H21C	109.5
Br2—Pd1—Br3^i^	176.25 (2)	H21B—C21—H21C	109.5
Br4—Pd1—Br3	177.07 (2)	C2—C22—H22A	109.5
Br2—Pd1—Br3	91.29 (2)	C2—C22—H22B	109.5
Br3^i^—Pd1—Br3	85.00 (2)	H22A—C22—H22B	109.5
Pd1—Br2—Br1	71.340 (18)	C2—C22—H22C	109.5
Pd1^i^—Br3—Pd1	95.00 (2)	H22A—C22—H22C	109.5
C13'—C1—C11'	112.2 (14)	H22B—C22—H22C	109.5
C13—C1—C12	109.0 (5)	C32'—C3—C33'	113.0 (18)
C13—C1—C11	109.1 (5)	C32'—C3—C31'	110.8 (15)
C12—C1—C11	108.1 (5)	C33'—C3—C31'	106.1 (16)
C13'—C1—C12'	106.8 (14)	C32—C3—C33	108.7 (5)
C11'—C1—C12'	105.9 (13)	C32—C3—C31	108.9 (6)
C13—C1—P1	110.9 (4)	C33—C3—C31	107.2 (6)
C13'—C1—P1	114.8 (12)	C32'—C3—P1	110.8 (15)
C11'—C1—P1	109.3 (11)	C32—C3—P1	113.6 (4)
C12—C1—P1	110.4 (4)	C33'—C3—P1	108.1 (19)
C11—C1—P1	109.3 (4)	C31'—C3—P1	107.7 (12)
C12'—C1—P1	107.4 (11)	C33—C3—P1	108.9 (4)
C1—C11—H11A	109.5	C31—C3—P1	109.4 (5)
C1—C11—H11B	109.5	C3—C31—H31A	109.5
H11A—C11—H11B	109.5	C3—C31—H31B	109.5
C1—C11—H11C	109.5	H31A—C31—H31B	109.5
H11A—C11—H11C	109.5	C3—C31—H31C	109.5
H11B—C11—H11C	109.5	H31A—C31—H31C	109.5
C1—C12—H12A	109.5	H31B—C31—H31C	109.5
C1—C12—H12B	109.5	C3—C32—H32A	109.5
H12A—C12—H12B	109.5	C3—C32—H32B	109.5
C1—C12—H12C	109.5	H32A—C32—H32B	109.5
H12A—C12—H12C	109.5	C3—C32—H32C	109.5
H12B—C12—H12C	109.5	H32A—C32—H32C	109.5
C1—C13—H13A	109.5	H32B—C32—H32C	109.5
C1—C13—H13B	109.5	C3—C33—H33A	109.5
H13A—C13—H13B	109.5	C3—C33—H33B	109.5
C1—C13—H13C	109.5	H33A—C33—H33B	109.5
H13A—C13—H13C	109.5	C3—C33—H33C	109.5
H13B—C13—H13C	109.5	H33A—C33—H33C	109.5
C1—C11'—H11D	109.5	H33B—C33—H33C	109.5
C1—C11'—H11E	109.5	C3—C31'—H31D	109.5
H11D—C11'—H11E	109.5	C3—C31'—H31E	109.5
C1—C11'—H11F	109.5	H31D—C31'—H31E	109.5
H11D—C11'—H11F	109.5	C3—C31'—H31F	109.5
H11E—C11'—H11F	109.5	H31D—C31'—H31F	109.5
C1—C12'—H12D	109.5	H31E—C31'—H31F	109.5
C1—C12'—H12E	109.5	C3—C32'—H32D	109.5
H12D—C12'—H12E	109.5	C3—C32'—H32E	109.5
C1—C12'—H12F	109.5	H32D—C32'—H32E	109.5
H12D—C12'—H12F	109.5	C3—C32'—H32F	109.5
H12E—C12'—H12F	109.5	H32D—C32'—H32F	109.5
C1—C13'—H13D	109.5	H32E—C32'—H32F	109.5
C1—C13'—H13E	109.5	C3—C33'—H33D	109.5
H13D—C13'—H13E	109.5	C3—C33'—H33E	109.5
C1—C13'—H13F	109.5	H33D—C33'—H33E	109.5
H13D—C13'—H13F	109.5	C3—C33'—H33F	109.5
H13E—C13'—H13F	109.5	H33D—C33'—H33F	109.5
C21—C2—C22	111.0 (5)	H33E—C33'—H33F	109.5
			
C2—P1—S1—Br1	41.39 (18)	C1—P1—C2—C21	−60.8 (4)
C1—P1—S1—Br1	161.38 (15)	C3—P1—C2—C21	169.0 (4)
C3—P1—S1—Br1	−77.83 (18)	S1—P1—C2—C21	50.2 (4)
Br4—Pd1—Br2—Br1	95.390 (19)	C1—P1—C2—C22	68.8 (5)
Br3—Pd1—Br2—Br1	−84.823 (19)	C3—P1—C2—C22	−61.4 (5)
Br2—Pd1—Br3—Pd1^i^	179.44 (2)	S1—P1—C2—C22	179.8 (4)
Br3^i^—Pd1—Br3—Pd1^i^	0.0	C2—P1—C3—C32'	−179.8 (19)
C2—P1—C1—C13	68.1 (5)	C1—P1—C3—C32'	50.0 (19)
C3—P1—C1—C13	−163.9 (5)	S1—P1—C3—C32'	−62.1 (19)
S1—P1—C1—C13	−46.9 (5)	C2—P1—C3—C32	83.8 (8)
C2—P1—C1—C13'	−80.4 (14)	C1—P1—C3—C32	−46.4 (8)
C3—P1—C1—C13'	47.6 (14)	S1—P1—C3—C32	−158.5 (7)
S1—P1—C1—C13'	164.6 (14)	C2—P1—C3—C33'	−55.5 (17)
C2—P1—C1—C11'	152.6 (14)	C1—P1—C3—C33'	174.4 (17)
C3—P1—C1—C11'	−79.4 (14)	S1—P1—C3—C33'	62.2 (17)
S1—P1—C1—C11'	37.6 (14)	C2—P1—C3—C31'	58.8 (17)
C2—P1—C1—C12	−171.1 (4)	C1—P1—C3—C31'	−71.4 (17)
C3—P1—C1—C12	−43.1 (5)	S1—P1—C3—C31'	176.5 (16)
S1—P1—C1—C12	74.0 (4)	C2—P1—C3—C33	−155.0 (6)
C2—P1—C1—C11	−52.3 (5)	C1—P1—C3—C33	74.8 (7)
C3—P1—C1—C11	75.7 (5)	S1—P1—C3—C33	−37.3 (7)
S1—P1—C1—C11	−167.3 (4)	C2—P1—C3—C31	−38.2 (7)
C2—P1—C1—C12'	38.2 (13)	C1—P1—C3—C31	−168.4 (6)
C3—P1—C1—C12'	166.2 (13)	S1—P1—C3—C31	79.5 (6)
S1—P1—C1—C12'	−76.8 (13)		

Symmetry code: (i) −*x*, −*y*+1, −*z*+1.

### (Bromosulfanyl)di-tert-butylisopropylphosphanium di-µ-bromido-bis[dibromidopalladium(II)] (7) Hydrogen-bond geometry (Å, º)

**Table:**

*D*—H···*A*	*D*—H	H···*A*	*D*···*A*	*D*—H···*A*
C11—H11*C*···Br4^ii^	0.98	3.04	4.005 (7)	167
C12—H12*C*···S1	0.98	3.03	3.547 (7)	114
C12—H12*C*···Br4^iii^	0.98	3.10	3.689 (6)	120
C13—H13*B*···S1	0.98	2.70	3.190 (7)	111
C2—H2···Br1	1.00	2.95	3.468 (5)	113
C2—H2···Br3^i^	1.00	3.12	3.928 (5)	139
C21—H21*B*···Br1	0.98	3.03	3.668 (6)	124
C21—H21*B*···S1	0.98	2.88	3.456 (6)	119
C21—H21*B*···Br3^iv^	0.98	3.10	3.968 (6)	149
C31—H31*C*···Br1	0.98	2.94	3.732 (11)	139
C33—H33*B*···S1	0.98	2.73	3.298 (10)	117

Symmetry codes: (i) −*x*, −*y*+1, −*z*+1; (ii) *x*+1, *y*, *z*+1; (iii) *x*+1, −*y*+3/2, *z*+1/2; (iv) −*x*+1, −*y*+1, −*z*+1.

### (Bromoselanyl)di-tert-butylisopropylphosphanium di-µ-bromido-bis[dibromidopalladium(II)] (8) Crystal data

**Table:**

(C_11_H_25_BrPS_2_)_2_[Pd_2_Br_6_]	*F*(000) = 2608
*M**_r_* = 1386.56	*D*_x_ = 2.433 Mg m^−^^3^
Monoclinic, *C*2/*c*	Mo *K*α radiation, λ = 0.71073 Å
*a* = 19.3550 (6) Å	Cell parameters from 7808 reflections
*b* = 14.8165 (2) Å	θ = 2.6–30.8°
*c* = 16.3047 (5) Å	µ = 11.42 mm^−^^1^
β = 125.957 (5)°	*T* = 100 K
*V* = 3784.8 (3) Å^3^	Prism, red
*Z* = 4	0.2 × 0.06 × 0.02 mm

### (Bromoselanyl)di-tert-butylisopropylphosphanium di-µ-bromido-bis[dibromidopalladium(II)] (8) Data collection

**Table:**

Oxford Diffraction Xcalibur, Eos diffractometer	5622 independent reflections
Radiation source: Enhance (Mo) X-ray Source	4433 reflections with *I* > 2σ(*I*)
Graphite monochromator	*R*_int_ = 0.074
Detector resolution: 16.1419 pixels mm^-1^	θ_max_ = 30.9°, θ_min_ = 2.6°
ω scans	*h* = −26→27
Absorption correction: multi-scan (CrysAlisPro; Rigaku OD, 2012)	*k* = −20→21
*T*_min_ = 0.327, *T*_max_ = 1.000	*l* = −23→23
51432 measured reflections	

### (Bromoselanyl)di-tert-butylisopropylphosphanium di-µ-bromido-bis[dibromidopalladium(II)] (8) Refinement

**Table:**

Refinement on *F*^2^	Primary atom site location: structure-invariant direct methods
Least-squares matrix: full	Secondary atom site location: difference Fourier map
*R*[*F*^2^ > 2σ(*F*^2^)] = 0.031	Hydrogen site location: inferred from neighbouring sites
*wR*(*F*^2^) = 0.057	H-atom parameters constrained
*S* = 1.04	*w* = 1/[σ^2^(*F*_o_^2^) + (0.0187*P*)^2^] where *P* = (*F*_o_^2^ + 2*F*_c_^2^)/3
5622 reflections	(Δ/σ)_max_ = 0.002
172 parameters	Δρ_max_ = 0.97 e Å^−^^3^
0 restraints	Δρ_min_ = −1.02 e Å^−^^3^

### (Bromoselanyl)di-tert-butylisopropylphosphanium di-µ-bromido-bis[dibromidopalladium(II)] (8) Fractional atomic coordinates and isotropic or equivalent isotropic displacement parameters (Å^2^)

**Table:**

	*x*	*y*	*z*	*U*_iso_*/*U*_eq_	
P1	0.22849 (5)	0.77872 (5)	0.24438 (6)	0.00853 (16)	
Se1	0.16673 (2)	0.87965 (2)	0.11698 (2)	0.01074 (7)	
Br1	0.28206 (2)	0.91565 (2)	0.11317 (2)	0.01416 (8)	
C1	0.13164 (18)	0.7218 (2)	0.2202 (2)	0.0114 (6)	
C11	0.1540 (2)	0.6693 (2)	0.3137 (2)	0.0195 (7)	
H11A	0.171302	0.711590	0.368829	0.029*	
H11B	0.201090	0.627609	0.334906	0.029*	
H11C	0.104049	0.635007	0.297163	0.029*	
C12	0.09563 (19)	0.6565 (2)	0.1310 (2)	0.0145 (7)	
H12A	0.043484	0.628429	0.115900	0.022*	
H12B	0.137963	0.609596	0.148605	0.022*	
H12C	0.082397	0.689711	0.071368	0.022*	
C13	0.06286 (19)	0.7918 (2)	0.1920 (2)	0.0154 (7)	
H13A	0.013385	0.761667	0.182044	0.023*	
H13B	0.045404	0.822396	0.129251	0.023*	
H13C	0.085754	0.836281	0.246658	0.023*	
C2	0.29749 (19)	0.7020 (2)	0.2326 (2)	0.0119 (6)	
H2	0.351708	0.735864	0.260464	0.014*	
C21	0.2632 (2)	0.6766 (2)	0.1232 (2)	0.0164 (7)	
H21A	0.308543	0.647598	0.122918	0.025*	
H21B	0.244019	0.731244	0.081387	0.025*	
H21C	0.215056	0.634815	0.095559	0.025*	
C22	0.3232 (2)	0.6161 (2)	0.2967 (2)	0.0180 (7)	
H22A	0.273840	0.575778	0.266646	0.027*	
H22B	0.343204	0.632082	0.365724	0.027*	
H22C	0.369143	0.585433	0.298861	0.027*	
C3	0.29514 (19)	0.8399 (2)	0.3681 (2)	0.0121 (6)	
C31	0.2387 (2)	0.8964 (2)	0.3853 (2)	0.0167 (7)	
H31A	0.205657	0.856415	0.398084	0.025*	
H31B	0.199669	0.933141	0.325067	0.025*	
H31C	0.274628	0.936049	0.443940	0.025*	
C32	0.3559 (2)	0.9036 (2)	0.3639 (2)	0.0160 (7)	
H32A	0.322466	0.947762	0.309403	0.024*	
H32B	0.391368	0.868362	0.350777	0.024*	
H32C	0.392649	0.935251	0.428725	0.024*	
C33	0.3513 (2)	0.7756 (2)	0.4582 (2)	0.0201 (8)	
H33A	0.381125	0.810013	0.521590	0.030*	
H33B	0.393280	0.746527	0.451519	0.030*	
H33C	0.315400	0.729465	0.458823	0.030*	
Pd1	0.500000	0.86021 (2)	0.250000	0.00908 (7)	
Pd2	0.500000	0.61741 (2)	0.250000	0.01122 (8)	
Br2	0.44677 (2)	0.97324 (2)	0.11858 (2)	0.01413 (7)	
Br3	0.45642 (2)	0.73949 (2)	0.12672 (2)	0.01349 (7)	
Br4	0.45006 (2)	0.50442 (2)	0.11976 (3)	0.01982 (8)	

### (Bromoselanyl)di-tert-butylisopropylphosphanium di-µ-bromido-bis[dibromidopalladium(II)] (8) Atomic displacement parameters (Å^2^)

**Table:**

	*U*^11^	*U*^22^	*U*^33^	*U*^12^	*U*^13^	*U*^23^
P1	0.0104 (4)	0.0066 (4)	0.0094 (4)	0.0001 (3)	0.0062 (3)	0.0001 (3)
Se1	0.01218 (15)	0.00876 (16)	0.01024 (15)	−0.00039 (12)	0.00600 (13)	0.00079 (12)
Br1	0.01752 (16)	0.01363 (17)	0.01470 (16)	−0.00296 (13)	0.01135 (14)	−0.00010 (13)
C1	0.0094 (14)	0.0096 (16)	0.0147 (16)	−0.0003 (12)	0.0068 (13)	0.0013 (12)
C11	0.0223 (18)	0.0178 (19)	0.0210 (18)	0.0002 (14)	0.0142 (16)	0.0059 (14)
C12	0.0120 (16)	0.0117 (17)	0.0179 (17)	−0.0019 (12)	0.0077 (14)	−0.0014 (13)
C13	0.0136 (16)	0.0163 (18)	0.0155 (17)	0.0002 (13)	0.0081 (14)	0.0000 (13)
C2	0.0115 (15)	0.0105 (16)	0.0147 (16)	−0.0001 (12)	0.0083 (13)	−0.0029 (12)
C21	0.0205 (17)	0.0141 (18)	0.0214 (18)	0.0018 (13)	0.0161 (15)	−0.0031 (14)
C22	0.0181 (17)	0.0145 (18)	0.0221 (18)	0.0049 (13)	0.0123 (15)	0.0060 (14)
C3	0.0132 (16)	0.0124 (17)	0.0101 (15)	−0.0019 (12)	0.0064 (13)	−0.0018 (12)
C31	0.0175 (17)	0.0179 (19)	0.0161 (17)	−0.0039 (13)	0.0106 (15)	−0.0057 (13)
C32	0.0177 (17)	0.0160 (18)	0.0150 (17)	−0.0059 (13)	0.0101 (14)	−0.0061 (13)
C33	0.0200 (18)	0.022 (2)	0.0098 (16)	0.0001 (14)	0.0042 (14)	0.0014 (14)
Pd1	0.01055 (16)	0.00756 (17)	0.00919 (16)	0.000	0.00583 (14)	0.000
Pd2	0.01087 (16)	0.00836 (17)	0.01348 (17)	0.000	0.00661 (14)	0.000
Br2	0.01631 (16)	0.01205 (17)	0.01485 (16)	0.00145 (12)	0.00962 (13)	0.00405 (12)
Br3	0.01799 (16)	0.01008 (16)	0.01034 (15)	−0.00096 (12)	0.00716 (13)	−0.00139 (12)
Br4	0.02289 (18)	0.01048 (17)	0.01998 (18)	0.00065 (13)	0.00918 (15)	−0.00316 (13)

### (Bromoselanyl)di-tert-butylisopropylphosphanium di-µ-bromido-bis[dibromidopalladium(II)] (8) Geometric parameters (Å, º)

**Table:**

P1—C2	1.848 (3)	C22—H22A	0.9800
P1—C3	1.870 (3)	C22—H22B	0.9800
P1—C1	1.875 (3)	C22—H22C	0.9800
P1—Se1	2.2505 (8)	C3—C31	1.528 (4)
Se1—Br1	2.3310 (4)	C3—C33	1.540 (4)
Br1—Br2	3.2510 (5)	C3—C32	1.542 (4)
C1—C11	1.529 (4)	C31—H31A	0.9800
C1—C13	1.530 (4)	C31—H31B	0.9800
C1—C12	1.532 (4)	C31—H31C	0.9800
C11—H11A	0.9800	C32—H32A	0.9800
C11—H11B	0.9800	C32—H32B	0.9800
C11—H11C	0.9800	C32—H32C	0.9800
C12—H12A	0.9800	C33—H33A	0.9800
C12—H12B	0.9800	C33—H33B	0.9800
C12—H12C	0.9800	C33—H33C	0.9800
C13—H13A	0.9800	Pd1—Br2	2.4218 (4)
C13—H13B	0.9800	Pd1—Br2^i^	2.4218 (4)
C13—H13C	0.9800	Pd1—Br3	2.4413 (4)
C2—C22	1.533 (4)	Pd1—Br3^i^	2.4413 (4)
C2—C21	1.541 (4)	Pd2—Br4^i^	2.4157 (4)
C2—H2	1.0000	Pd2—Br4	2.4157 (4)
C21—H21A	0.9800	Pd2—Br3^i^	2.4562 (4)
C21—H21B	0.9800	Pd2—Br3	2.4562 (4)
C21—H21C	0.9800		
			
C2—P1—C3	109.11 (14)	C2—C22—H22A	109.5
C2—P1—C1	113.31 (14)	C2—C22—H22B	109.5
C3—P1—C1	114.58 (14)	H22A—C22—H22B	109.5
C2—P1—Se1	109.72 (10)	C2—C22—H22C	109.5
C3—P1—Se1	109.24 (10)	H22A—C22—H22C	109.5
C1—P1—Se1	100.47 (10)	H22B—C22—H22C	109.5
P1—Se1—Br1	100.30 (2)	C31—C3—C33	110.1 (3)
Se1—Br1—Br2	176.810 (16)	C31—C3—C32	108.6 (3)
C11—C1—C13	109.4 (3)	C33—C3—C32	106.9 (3)
C11—C1—C12	109.3 (3)	C31—C3—P1	110.4 (2)
C13—C1—C12	108.2 (2)	C33—C3—P1	112.5 (2)
C11—C1—P1	110.9 (2)	C32—C3—P1	108.0 (2)
C13—C1—P1	110.1 (2)	C3—C31—H31A	109.5
C12—C1—P1	108.9 (2)	C3—C31—H31B	109.5
C1—C11—H11A	109.5	H31A—C31—H31B	109.5
C1—C11—H11B	109.5	C3—C31—H31C	109.5
H11A—C11—H11B	109.5	H31A—C31—H31C	109.5
C1—C11—H11C	109.5	H31B—C31—H31C	109.5
H11A—C11—H11C	109.5	C3—C32—H32A	109.5
H11B—C11—H11C	109.5	C3—C32—H32B	109.5
C1—C12—H12A	109.5	H32A—C32—H32B	109.5
C1—C12—H12B	109.5	C3—C32—H32C	109.5
H12A—C12—H12B	109.5	H32A—C32—H32C	109.5
C1—C12—H12C	109.5	H32B—C32—H32C	109.5
H12A—C12—H12C	109.5	C3—C33—H33A	109.5
H12B—C12—H12C	109.5	C3—C33—H33B	109.5
C1—C13—H13A	109.5	H33A—C33—H33B	109.5
C1—C13—H13B	109.5	C3—C33—H33C	109.5
H13A—C13—H13B	109.5	H33A—C33—H33C	109.5
C1—C13—H13C	109.5	H33B—C33—H33C	109.5
H13A—C13—H13C	109.5	Br2—Pd1—Br2^i^	92.495 (19)
H13B—C13—H13C	109.5	Br2—Pd1—Br3	90.936 (11)
C22—C2—C21	109.5 (3)	Br2^i^—Pd1—Br3	175.531 (13)
C22—C2—P1	113.9 (2)	Br2—Pd1—Br3^i^	175.531 (13)
C21—C2—P1	115.1 (2)	Br2^i^—Pd1—Br3^i^	90.936 (11)
C22—C2—H2	105.8	Br3—Pd1—Br3^i^	85.785 (18)
C21—C2—H2	105.8	Br4^i^—Pd2—Br4	92.26 (2)
P1—C2—H2	105.8	Br4^i^—Pd2—Br3^i^	91.326 (11)
C2—C21—H21A	109.5	Br4—Pd2—Br3^i^	176.053 (14)
C2—C21—H21B	109.5	Br4^i^—Pd2—Br3	176.054 (14)
H21A—C21—H21B	109.5	Br4—Pd2—Br3	91.324 (11)
C2—C21—H21C	109.5	Br3^i^—Pd2—Br3	85.139 (18)
H21A—C21—H21C	109.5	Pd1—Br2—Br1	75.133 (10)
H21B—C21—H21C	109.5	Pd1—Br3—Pd2	94.538 (13)
			
C2—P1—Se1—Br1	−42.39 (11)	Se1—P1—C2—C22	−164.30 (19)
C3—P1—Se1—Br1	77.19 (11)	C3—P1—C2—C21	−156.3 (2)
C1—P1—Se1—Br1	−161.98 (10)	C1—P1—C2—C21	74.7 (3)
C2—P1—C1—C11	80.0 (2)	Se1—P1—C2—C21	−36.6 (2)
C3—P1—C1—C11	−46.1 (3)	C2—P1—C3—C31	−174.0 (2)
Se1—P1—C1—C11	−163.1 (2)	C1—P1—C3—C31	−45.8 (3)
C2—P1—C1—C13	−158.8 (2)	Se1—P1—C3—C31	66.0 (2)
C3—P1—C1—C13	75.1 (2)	C2—P1—C3—C33	−50.5 (3)
Se1—P1—C1—C13	−41.9 (2)	C1—P1—C3—C33	77.8 (3)
C2—P1—C1—C12	−40.3 (2)	Se1—P1—C3—C33	−170.4 (2)
C3—P1—C1—C12	−166.4 (2)	C2—P1—C3—C32	67.3 (2)
Se1—P1—C1—C12	76.6 (2)	C1—P1—C3—C32	−164.4 (2)
C3—P1—C2—C22	76.0 (3)	Se1—P1—C3—C32	−52.6 (2)
C1—P1—C2—C22	−52.9 (3)		

Symmetry code: (i) −*x*+1, *y*, −*z*+1/2.

### (Bromoselanyl)di-tert-butylisopropylphosphanium di-µ-bromido-bis[dibromidopalladium(II)] (8) Hydrogen-bond geometry (Å, º)

**Table:**

*D*—H···*A*	*D*—H	H···*A*	*D*···*A*	*D*—H···*A*
C12—H12*C*···Se1	0.98	3.12	3.640 (3)	115
C13—H13*B*···Se1	0.98	2.61	3.181 (3)	117
C21—H21*A*···Br3	0.98	3.14	3.822 (3)	128
C21—H21*B*···Se1	0.98	2.90	3.511 (3)	121
C21—H21*B*···Br1	0.98	2.80	3.574 (3)	137
C31—H31*B*···Se1	0.98	3.16	3.740 (3)	119
C32—H32*A*···Se1	0.98	2.97	3.526 (3)	117
C32—H32*A*···Br1	0.98	2.85	3.452 (3)	120
C12—H12*A*···Br2^ii^	0.98	2.98	3.877 (3)	152
C12—H12*C*···Br3^iii^	0.98	3.04	4.009 (3)	170
C2—H2···Br3^i^	1.00	3.03	3.928 (3)	151
C32—H32*C*···Br2^iv^	0.98	2.95	3.885 (3)	160

Symmetry codes: (i) −*x*+1, *y*, −*z*+1/2; (ii) *x*−1/2, *y*−1/2, *z*; (iii) −*x*+1/2, −*y*+3/2, −*z*; (iv) *x*, −*y*+2, *z*+1/2.

### Bis[dimethyl(sulfanylidene)phosphinito-κSe]\ bis(hydroxydiisopropylphosphine selenide-κSe)palladium(II) (9) Crystal data

**Table:**

[Pd(C_6_H_14_OP)_2_(C_6_H_15_OP)_2_]	*F*(000) = 952
*M**_r_* = 956.82	*D*_x_ = 1.726 Mg m^−^^3^
Monoclinic, *P*2_1_/*n*	Mo *K*α radiation, λ = 0.71073 Å
*a* = 7.56435 (6) Å	Cell parameters from 28620 reflections
*b* = 10.09140 (9) Å	θ = 2.2–30.8°
*c* = 24.13960 (19) Å	µ = 4.66 mm^−^^1^
β = 92.7641 (8)°	*T* = 100 K
*V* = 1840.55 (3) Å^3^	Plate, dichroic orange yellow
*Z* = 2	0.10 × 0.08 × 0.04 mm

### Bis[dimethyl(sulfanylidene)phosphinito-κSe]\ bis(hydroxydiisopropylphosphine selenide-κSe)palladium(II) (9) Data collection

**Table:**

Oxford Diffraction Xcalibur, Eos diffractometer	5558 independent reflections
Radiation source: Enhance (Mo) X-ray Source	4939 reflections with *I* > 2σ(*I*)
Graphite monochromator	*R*_int_ = 0.047
Detector resolution: 16.1419 pixels mm^-1^	θ_max_ = 30.9°, θ_min_ = 2.2°
ω scans	*h* = −10→10
Absorption correction: multi-scan (CrysAlisPro; Rigaku OD, 2012)	*k* = −14→14
*T*_min_ = 0.650, *T*_max_ = 1.000	*l* = −34→34
86102 measured reflections	

### Bis[dimethyl(sulfanylidene)phosphinito-κSe]\ bis(hydroxydiisopropylphosphine selenide-κSe)palladium(II) (9) Refinement

**Table:**

Refinement on *F*^2^	Primary atom site location: structure-invariant direct methods
Least-squares matrix: full	Secondary atom site location: difference Fourier map
*R*[*F*^2^ > 2σ(*F*^2^)] = 0.022	Hydrogen site location: mixed
*wR*(*F*^2^) = 0.041	H atoms treated by a mixture of independent and constrained refinement
*S* = 1.07	*w* = 1/[σ^2^(*F*_o_^2^) + (0.0132*P*)^2^ + 1.1714*P*] where *P* = (*F*_o_^2^ + 2*F*_c_^2^)/3
5558 reflections	(Δ/σ)_max_ = 0.001
185 parameters	Δρ_max_ = 0.47 e Å^−^^3^
2 restraints	Δρ_min_ = −0.54 e Å^−^^3^

### Bis[dimethyl(sulfanylidene)phosphinito-κSe]\ bis(hydroxydiisopropylphosphine selenide-κSe)palladium(II) (9) Fractional atomic coordinates and isotropic or equivalent isotropic displacement parameters (Å^2^)

**Table:**

	*x*	*y*	*z*	*U*_iso_*/*U*_eq_	Occ. (<1)
Pd1	0.500000	0.500000	0.000000	0.01153 (4)	
Se1	0.57000 (2)	0.38861 (2)	0.08922 (2)	0.01535 (4)	
Se2	0.41580 (2)	0.71744 (2)	0.03720 (2)	0.01366 (4)	
P1	0.43798 (6)	0.47524 (4)	0.15938 (2)	0.01456 (8)	
P2	0.65706 (5)	0.79940 (4)	0.07727 (2)	0.01312 (8)	
O1	0.49525 (18)	0.61413 (12)	0.17883 (5)	0.0217 (3)	
H01	0.569 (4)	0.653 (3)	0.1640 (15)	0.012 (10)*	0.5
O2	0.72157 (16)	0.73009 (12)	0.13081 (5)	0.0173 (2)	
H02	0.651 (5)	0.686 (4)	0.1445 (17)	0.026 (12)*	0.5
C1	0.4898 (2)	0.36446 (17)	0.21777 (7)	0.0185 (3)	
H1	0.444759	0.273758	0.208155	0.022*	
C2	0.1998 (2)	0.47669 (19)	0.14275 (8)	0.0233 (4)	
H2	0.184451	0.507699	0.103463	0.028*	
C3	0.8443 (2)	0.80181 (17)	0.03261 (7)	0.0183 (3)	
H3	0.944534	0.845492	0.054113	0.022*	
C4	0.6011 (2)	0.97035 (16)	0.09352 (7)	0.0165 (3)	
H4	0.560610	1.016268	0.058455	0.020*	
C11	0.6902 (2)	0.3574 (2)	0.22936 (8)	0.0276 (4)	
H11A	0.736533	0.446633	0.236850	0.041*	
H11B	0.746032	0.320366	0.196994	0.041*	
H11C	0.716396	0.300656	0.261670	0.041*	
C12	0.3978 (3)	0.4140 (2)	0.26924 (7)	0.0251 (4)	
H12A	0.444268	0.365769	0.302042	0.038*	
H12B	0.270059	0.398763	0.264315	0.038*	
H12C	0.420437	0.508956	0.274180	0.038*	
C21	0.1008 (3)	0.5759 (2)	0.17752 (10)	0.0403 (5)	
H21A	−0.020928	0.585362	0.162203	0.061*	
H21B	0.160572	0.661983	0.176856	0.061*	
H21C	0.099058	0.544047	0.215846	0.061*	
C22	0.1194 (2)	0.3383 (2)	0.14410 (8)	0.0287 (4)	
H22A	0.116893	0.307857	0.182601	0.043*	
H22B	0.191026	0.277268	0.122951	0.043*	
H22C	−0.001539	0.340790	0.127601	0.043*	
C31	0.8062 (2)	0.88557 (19)	−0.01933 (7)	0.0233 (4)	
H31A	0.911658	0.888141	−0.041347	0.035*	
H31B	0.774974	0.975846	−0.008477	0.035*	
H31C	0.707527	0.846427	−0.041469	0.035*	
C32	0.9062 (2)	0.66192 (19)	0.01842 (8)	0.0224 (4)	
H32A	0.817095	0.619247	−0.006420	0.034*	
H32B	0.922430	0.609846	0.052553	0.034*	
H32C	1.018726	0.667126	0.000034	0.034*	
C41	0.4514 (2)	0.97368 (18)	0.13373 (8)	0.0231 (4)	
H41A	0.488956	0.927620	0.168046	0.035*	
H41B	0.346770	0.929646	0.116767	0.035*	
H41C	0.422435	1.065926	0.142169	0.035*	
C42	0.7641 (2)	1.04303 (18)	0.11869 (8)	0.0248 (4)	
H42A	0.731021	1.133533	0.128663	0.037*	
H42B	0.856172	1.046052	0.091527	0.037*	
H42C	0.809062	0.995924	0.151958	0.037*	

### Bis[dimethyl(sulfanylidene)phosphinito-κSe]\ bis(hydroxydiisopropylphosphine selenide-κSe)palladium(II) (9) Atomic displacement parameters (Å^2^)

**Table:**

	*U*^11^	*U*^22^	*U*^33^	*U*^12^	*U*^13^	*U*^23^
Pd1	0.01300 (8)	0.01194 (8)	0.00948 (8)	−0.00030 (6)	−0.00123 (6)	0.00009 (6)
Se1	0.01935 (8)	0.01597 (8)	0.01067 (8)	0.00335 (6)	0.00021 (6)	0.00170 (6)
Se2	0.01356 (7)	0.01384 (8)	0.01338 (8)	0.00039 (6)	−0.00162 (6)	−0.00140 (6)
P1	0.01670 (19)	0.0150 (2)	0.01200 (19)	0.00073 (16)	0.00040 (15)	0.00122 (16)
P2	0.01415 (19)	0.01285 (19)	0.01223 (19)	−0.00036 (15)	−0.00065 (15)	−0.00012 (15)
O1	0.0318 (7)	0.0181 (6)	0.0155 (6)	−0.0043 (5)	0.0057 (5)	−0.0010 (5)
O2	0.0192 (6)	0.0159 (6)	0.0164 (6)	−0.0012 (5)	−0.0035 (5)	0.0032 (5)
C1	0.0223 (8)	0.0198 (8)	0.0134 (8)	0.0003 (7)	0.0018 (6)	0.0035 (6)
C2	0.0184 (8)	0.0296 (10)	0.0216 (9)	0.0059 (7)	−0.0011 (7)	−0.0001 (8)
C3	0.0144 (7)	0.0222 (9)	0.0183 (8)	−0.0026 (6)	−0.0003 (6)	−0.0024 (7)
C4	0.0209 (8)	0.0127 (8)	0.0157 (8)	0.0006 (6)	0.0002 (6)	0.0006 (6)
C11	0.0257 (9)	0.0415 (12)	0.0155 (9)	0.0067 (8)	−0.0017 (7)	0.0058 (8)
C12	0.0282 (9)	0.0324 (11)	0.0149 (8)	−0.0012 (8)	0.0048 (7)	0.0030 (8)
C21	0.0283 (11)	0.0401 (13)	0.0526 (14)	0.0160 (10)	0.0014 (10)	−0.0102 (11)
C22	0.0185 (9)	0.0387 (11)	0.0290 (10)	−0.0051 (8)	0.0022 (7)	−0.0067 (9)
C31	0.0249 (9)	0.0251 (9)	0.0203 (9)	−0.0038 (7)	0.0058 (7)	0.0011 (7)
C32	0.0153 (8)	0.0265 (9)	0.0255 (9)	0.0027 (7)	0.0005 (7)	−0.0061 (8)
C41	0.0270 (9)	0.0212 (9)	0.0215 (9)	0.0032 (7)	0.0055 (7)	−0.0036 (7)
C42	0.0278 (9)	0.0164 (9)	0.0299 (10)	−0.0041 (7)	−0.0030 (8)	−0.0042 (7)

### Bis[dimethyl(sulfanylidene)phosphinito-κSe]\ bis(hydroxydiisopropylphosphine selenide-κSe)palladium(II) (9) Geometric parameters (Å, º)

**Table:**

Pd1—Se1	2.4642 (2)	C4—H4	1.0000
Pd1—Se1^i^	2.4642 (2)	C11—H11A	0.9800
Pd1—Se2^i^	2.4662 (2)	C11—H11B	0.9800
Pd1—Se2	2.4662 (2)	C11—H11C	0.9800
Se1—P1	2.1894 (5)	C12—H12A	0.9800
Se2—P2	2.1863 (4)	C12—H12B	0.9800
P1—O1	1.5340 (13)	C12—H12C	0.9800
P1—C1	1.8267 (17)	C21—H21A	0.9800
P1—C2	1.8272 (18)	C21—H21B	0.9800
P2—O2	1.5287 (12)	C21—H21C	0.9800
P2—C3	1.8213 (17)	C22—H22A	0.9800
P2—C4	1.8234 (17)	C22—H22B	0.9800
O1—H01	0.782 (18)	C22—H22C	0.9800
O2—H02	0.778 (19)	C31—H31A	0.9800
C1—C11	1.530 (2)	C31—H31B	0.9800
C1—C12	1.536 (2)	C31—H31C	0.9800
C1—H1	1.0000	C32—H32A	0.9800
C2—C22	1.524 (3)	C32—H32B	0.9800
C2—C21	1.526 (3)	C32—H32C	0.9800
C2—H2	1.0000	C41—H41A	0.9800
C3—C31	1.528 (2)	C41—H41B	0.9800
C3—C32	1.531 (2)	C41—H41C	0.9800
C3—H3	1.0000	C42—H42A	0.9800
C4—C41	1.527 (2)	C42—H42B	0.9800
C4—C42	1.534 (2)	C42—H42C	0.9800
			
Se1—Pd1—Se1^i^	180.0	C1—C11—H11B	109.5
Se1—Pd1—Se2^i^	82.184 (5)	H11A—C11—H11B	109.5
Se1^i^—Pd1—Se2^i^	97.816 (5)	C1—C11—H11C	109.5
Se1—Pd1—Se2	97.817 (5)	H11A—C11—H11C	109.5
Se1^i^—Pd1—Se2	82.183 (5)	H11B—C11—H11C	109.5
Se2^i^—Pd1—Se2	180.0	C1—C12—H12A	109.5
P1—Se1—Pd1	114.028 (13)	C1—C12—H12B	109.5
P2—Se2—Pd1	105.840 (13)	H12A—C12—H12B	109.5
O1—P1—C1	106.03 (8)	C1—C12—H12C	109.5
O1—P1—C2	108.72 (8)	H12A—C12—H12C	109.5
C1—P1—C2	110.31 (8)	H12B—C12—H12C	109.5
O1—P1—Se1	117.97 (5)	C2—C21—H21A	109.5
C1—P1—Se1	105.29 (6)	C2—C21—H21B	109.5
C2—P1—Se1	108.36 (6)	H21A—C21—H21B	109.5
O2—P2—C3	106.31 (7)	C2—C21—H21C	109.5
O2—P2—C4	108.60 (7)	H21A—C21—H21C	109.5
C3—P2—C4	108.05 (8)	H21B—C21—H21C	109.5
O2—P2—Se2	115.25 (5)	C2—C22—H22A	109.5
C3—P2—Se2	113.56 (6)	C2—C22—H22B	109.5
C4—P2—Se2	104.81 (6)	H22A—C22—H22B	109.5
P1—O1—H01	121 (3)	C2—C22—H22C	109.5
P2—O2—H02	115 (3)	H22A—C22—H22C	109.5
C11—C1—C12	110.52 (15)	H22B—C22—H22C	109.5
C11—C1—P1	110.08 (12)	C3—C31—H31A	109.5
C12—C1—P1	109.65 (12)	C3—C31—H31B	109.5
C11—C1—H1	108.8	H31A—C31—H31B	109.5
C12—C1—H1	108.8	C3—C31—H31C	109.5
P1—C1—H1	108.8	H31A—C31—H31C	109.5
C22—C2—C21	112.48 (17)	H31B—C31—H31C	109.5
C22—C2—P1	112.23 (13)	C3—C32—H32A	109.5
C21—C2—P1	112.91 (14)	C3—C32—H32B	109.5
C22—C2—H2	106.2	H32A—C32—H32B	109.5
C21—C2—H2	106.2	C3—C32—H32C	109.5
P1—C2—H2	106.2	H32A—C32—H32C	109.5
C31—C3—C32	111.96 (15)	H32B—C32—H32C	109.5
C31—C3—P2	111.82 (12)	C4—C41—H41A	109.5
C32—C3—P2	112.02 (12)	C4—C41—H41B	109.5
C31—C3—H3	106.9	H41A—C41—H41B	109.5
C32—C3—H3	106.9	C4—C41—H41C	109.5
P2—C3—H3	106.9	H41A—C41—H41C	109.5
C41—C4—C42	110.11 (15)	H41B—C41—H41C	109.5
C41—C4—P2	110.15 (12)	C4—C42—H42A	109.5
C42—C4—P2	110.32 (12)	C4—C42—H42B	109.5
C41—C4—H4	108.7	H42A—C42—H42B	109.5
C42—C4—H4	108.7	C4—C42—H42C	109.5
P2—C4—H4	108.7	H42A—C42—H42C	109.5
C1—C11—H11A	109.5	H42B—C42—H42C	109.5
			
Se2^i^—Pd1—Se1—P1	−156.179 (15)	C1—P1—C2—C22	−42.75 (16)
Se2—Pd1—Se1—P1	23.820 (15)	Se1—P1—C2—C22	72.03 (14)
Se1—Pd1—Se2—P2	65.789 (13)	O1—P1—C2—C21	−30.21 (17)
Se1^i^—Pd1—Se2—P2	−114.210 (13)	C1—P1—C2—C21	85.66 (17)
Pd1—Se1—P1—O1	−68.57 (6)	Se1—P1—C2—C21	−159.57 (14)
Pd1—Se1—P1—C1	173.44 (6)	O2—P2—C3—C31	−172.02 (12)
Pd1—Se1—P1—C2	55.41 (7)	C4—P2—C3—C31	−55.61 (14)
Pd1—Se2—P2—O2	−70.31 (6)	Se2—P2—C3—C31	60.20 (13)
Pd1—Se2—P2—C3	52.68 (6)	O2—P2—C3—C32	61.36 (13)
Pd1—Se2—P2—C4	170.38 (6)	C4—P2—C3—C32	177.78 (12)
O1—P1—C1—C11	−65.74 (14)	Se2—P2—C3—C32	−66.41 (13)
C2—P1—C1—C11	176.72 (13)	O2—P2—C4—C41	−61.77 (13)
Se1—P1—C1—C11	60.02 (13)	C3—P2—C4—C41	−176.69 (12)
O1—P1—C1—C12	56.06 (14)	Se2—P2—C4—C41	61.91 (12)
C2—P1—C1—C12	−61.48 (15)	O2—P2—C4—C42	60.00 (14)
Se1—P1—C1—C12	−178.17 (11)	C3—P2—C4—C42	−54.92 (14)
O1—P1—C2—C22	−158.62 (13)	Se2—P2—C4—C42	−176.32 (11)

Symmetry code: (i) −*x*+1, −*y*+1, −*z*.

### Bis[dimethyl(sulfanylidene)phosphinito-κSe]\ bis(hydroxydiisopropylphosphine selenide-κSe)palladium(II) (9) Hydrogen-bond geometry (Å, º)

**Table:**

*D*—H···*A*	*D*—H	H···*A*	*D*···*A*	*D*—H···*A*
O1—H01···O2	0.78 (2)	1.63 (2)	2.4156 (17)	178 (4)
O2—H02···O1	0.78 (2)	1.64 (2)	2.4156 (17)	172 (5)
C11—H11*B*···Se1	0.98	2.95	3.4748 (18)	115
C21—H21*A*···O2^ii^	0.98	2.52	3.407 (2)	150
C32—H32*C*···Se2^iii^	0.98	3.13	3.8999 (17)	136
C41—H41*B*···Se2	0.98	2.94	3.4825 (18)	116
C42—H42*A*···Se1^iv^	0.98	2.98	3.8370 (19)	146
C32—H32*A*···Pd1	0.98	2.69	3.4897 (18)	139

Symmetry codes: (ii) *x*−1, *y*, *z*; (iii) *x*+1, *y*, *z*; (iv) *x*, *y*+1, *z*.
